# Supplementary material for: Verubulin (Azixa) Analogues with Increased Saturation: Synthesis, SAR and Encapsulation in Biocompatible Nanocontainers Based on Ca2+ or Mg2+ Cross-Linked Alginate
Source: Pharmaceuticals (Basel). 2023 Oct 21;16(10):1499. doi: 10.3390/ph16101499 (PMC10610134; doi:10.3390/ph16101499)

Verubulin (Azixa) Analogues with Increased Saturation: Synthesis, SAR and Encapsulation in Biocompatible Nanocontainers Based on Ca^{2+} or Mg^{2+} Cross-Linked Alginate

Kseniya N. Sedenkova [1], Denis N. Leschukov [1], Yuri K. Grishin [1], Nikolay A. Zefirov [1], Yulia A. Gracheva [1], Dmitry A. Skvortsov [1], Yanislav S. Hrytseniuk [1], Lilja A. Vasilyeva [2], Elena A. Spirkova [3], Pavel N. Shevtsov [3], Elena F. Shevtsova [3], Alina R. Lukmanova [1], Vasily V. Spiridonov [1], Alina A. Markova [4], Minh T. Nguyen [4], Alexander A. Shtil [1,5], Olga N. Zefirova [1], Alexander A. Yaroslavov [1], Elena R. Milaeva [1] and Elena B. Averina [1,*]

[1] Department of Chemistry, Lomonosov Moscow State University, 119991 Moscow, Russia; sedenkova@med.chem.msu.ru (K.N.S.), denis.leshchukov@chemistry.msu.ru (D.N.L.), grishin@nmr.chem.msu.ru (Y.K.G.), kolaz92@gmail.com (N.A.Z.), jullina74@mail.ru (Y.A.G.), skvorratd@mail.ru (D.A.S.), gritseniuk2000@yandex.ru (Y.S.H.), lukmanovaalina@mail.ru (A.R.L.), vasya_spiridonov@mail.ru (V.V.S.), olgaz_13@mail.ru (O.N.S.), yaroslav@belozersky.msu.ru (A.A.Y.), milaeva@med.chem.msu.ru (E.R.M.), elaver@med.chem.msu.ru (E.B.A.)

[2] Faculty of Bioengineering and Bioinformatics, Lomonosov Moscow State University, 119991 Moscow, Russia; liljavasilyeva@gmail.com

[3] Institute of Physiologically Active Compounds at Federal Research Center of Problems of Chemical Physics and Medicinal Chemistry, Russian Academy of Sciences (IPAC RAS), 142432 Chernogolovka, Russia; kustova.ea@mail.ru (E.A.S.), pnshevtsov@gmail.com (P.N.S.), shevtsova@ipac.ac.ru (E.F.S.),

[4] Emanuel Institute of Biochemical Physics, Russian Academy of Sciences, 119334 Moscow, Russia; alenmark25@gmail.com (A.A.M.), tuantonyx@yahoo.com (M.T.N.)

[5] Institute of Cyber Intelligence Systems, National Research Nuclear University MEPhI, 115409 Moscow, Russia; shtilaa@yahoo.com

* Correspondence: elaver@med.chem.msu.ru

Supplementary materials

1. Dose-response dependencies of cytotoxicity of Verubulin (**1**) and compounds **2a-r**……..…..2

2. Cytotoxicity towards HCT116 cell line for selected compounds……………………...………..3

3. Dose-response dependencies for tubulin polymerization inhibition by compounds **1,2c,e**…...4

4. Preparation and characterization of nanocontainers filled with compound **2c**………………….5

5. Cells survival curves for nanocontainers filled with compound **2c**………………………………8

6. Copies of NMR spectra……………………………………………………………………………..9

1. Dose-response dependencies of cytotoxicity of verubulin (1) and compounds 2a-r

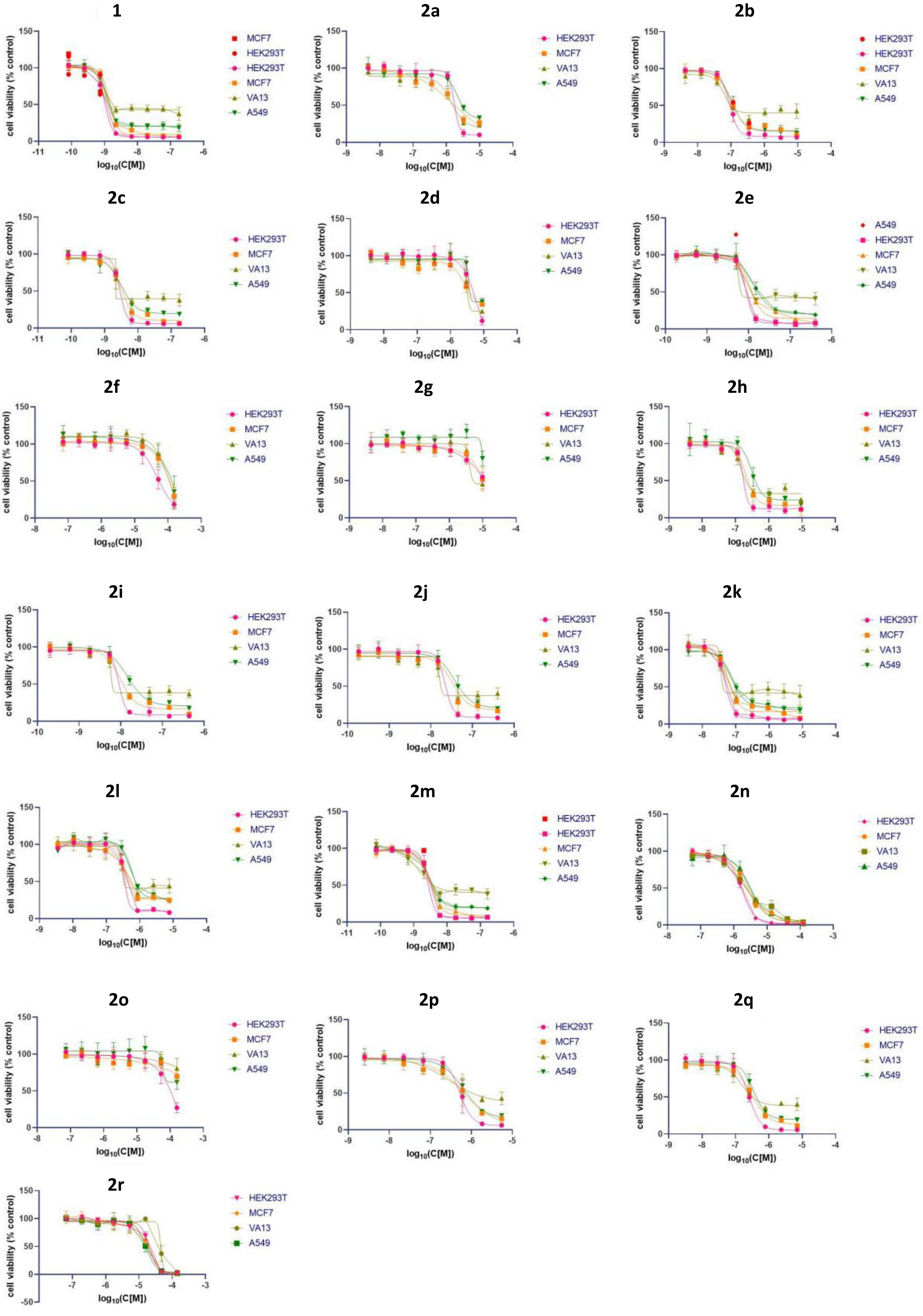

Figure S1. Dose-response dependencies of compounds’ cytotoxicity for different cell lines.

2. Cytotoxicity towards HCT116 cell line for selected compounds

Table S1. Cytotoxicity effects towards HCT116[1] cell line

**Compound**		**IC$_{50}$, nM**
**1**	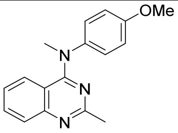	3.2 ± 0.7
**2c**	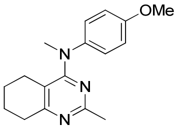	0.2 ± 0.01
**2e**	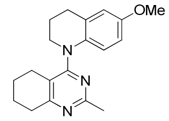	22 ± 5
**2i**	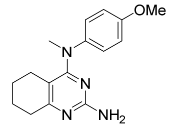	40 ±10
**2j**	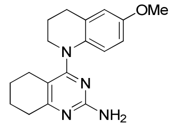	160 ± 30
**2m**	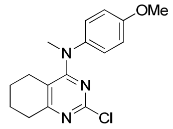	1.8 ± 0.1

[1] human colon carcinoma

3. Dose-response dependencies for tubulin polymerization inhibition by compounds 1,2c,e

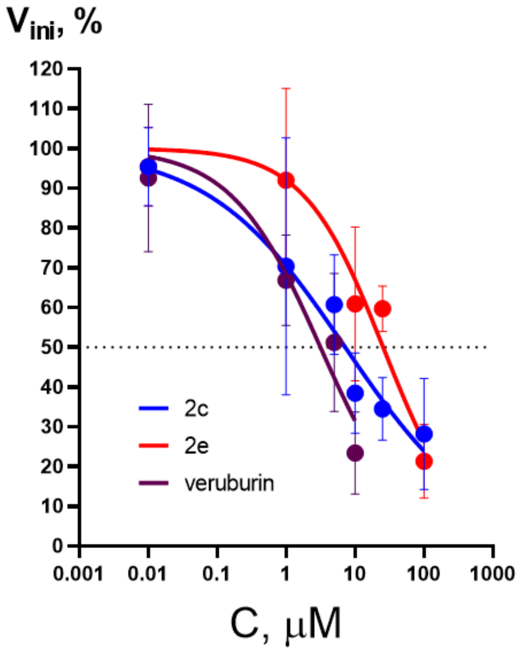

Figure S2. Dose-dependence of initial rates of the linear time-change in optical density at 355 nm (V_{ini}), normalized to V_{ini} of control probe in each experiment.

4. Preparation and characterization of nanocontainers filled with compound 2c

In order to immobilize aminopyrimidine **2c** with nanocontainers, it was preliminarily transformed into a protonated form **2c**·HCl (Scheme S1). To confirm the preservation of the aminopyrimidine ring in acidic conditions, a control experiment was carried out, consisting in the treatment of compound **2c** with HCl, subsequent neutralization of the reaction mixture, isolation of heterocycle, and ^{1}H NMR method controlling (Figure S3).

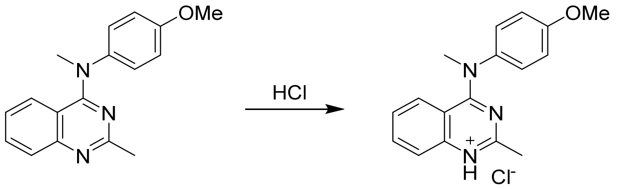

Scheme S1. Protonation of compound **2c**.

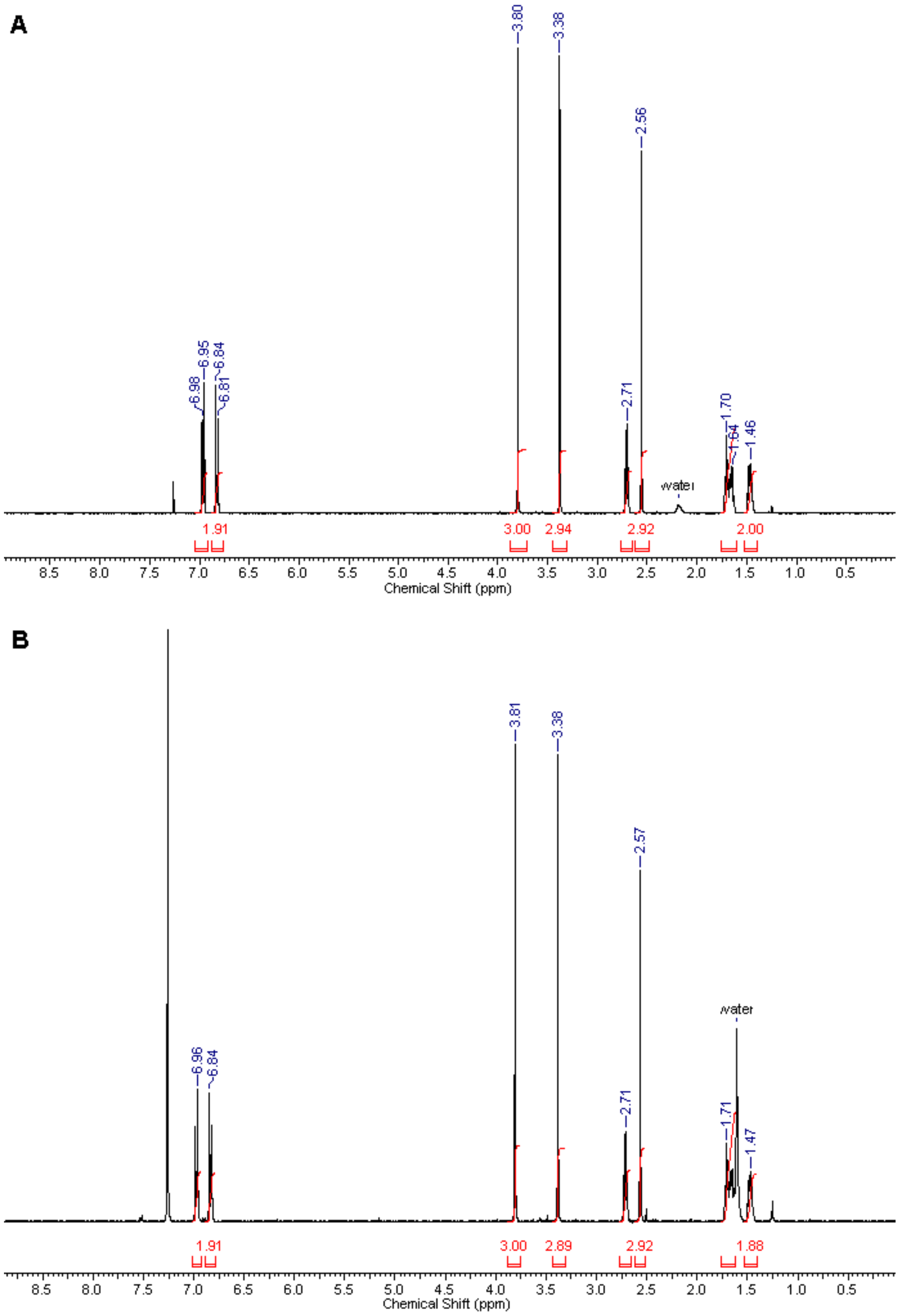

Figure S3. ^{1}H NMR spectra of compound **2c**: **A –** before treatment with HCl; **B** – after treatment with HCl and subsequent neutralization.

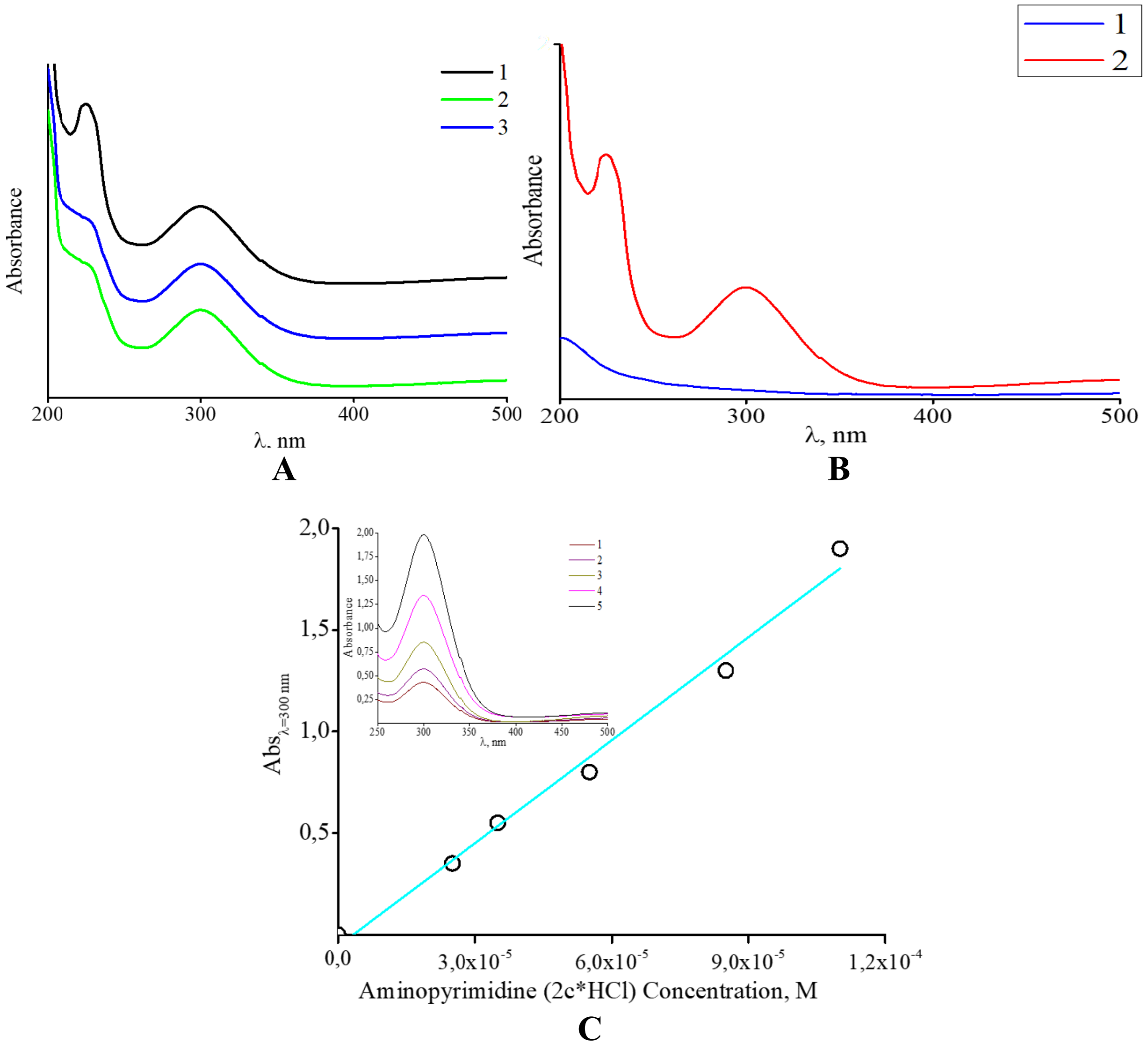

Figure S4. A) UV/vis-spectra: 1 – Aminopyrimidine **2c**·HCl, 2 – Aminopyrimidine **2c**·HCl in a presence of nanocontainer Ca^{2+}-Alg, 3 – Aminopyrimidine **2c**·HCl in a presence of nanocontainer Mg^{2+}-Alg. **B)** UV/vis-spectra: 1– External dialysis solution after the purification of nanocontainer Ca^{2+}-Alg filled with Aminopyrimidine **2c**·HCl; 2 – Aminopyrimidine **2c**·HCl. **C)** Calibration plot Absorbance (λ=300 nm) *vs* C (Concentration of Aminopyrimidine **2c**·HCl): In a top left UV/vis-spectra: 1 – C(**2c**·HCl) = 2.5×10^{-5} M; 2 – C(**2c**·HCl) = 3.5×10^{-5} M; 3 – C(**2c**·HCl) = 5.5×10^{-5} M; 4– C(**2c**·HCl) = 8.5×10-5 M, 5 – C(**2c**·HCl) = 1.1×10^{-4} M.

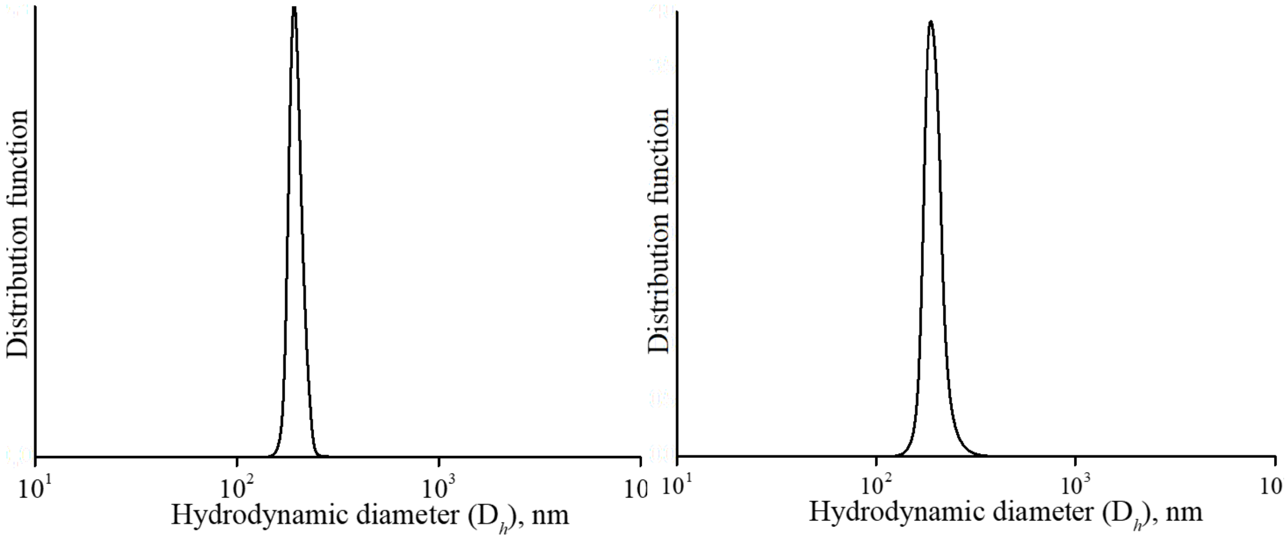

a) **b)**

Figure S5. a) Distribution function of nanocontainer Ca^{2+}-Alg particles in an aqueous media; b) Distribution function of nanocontainer Mg^{2+}-Alg particles in an aqueous media.

5. Cells survival curves for nanocontainers filled with compound 2c

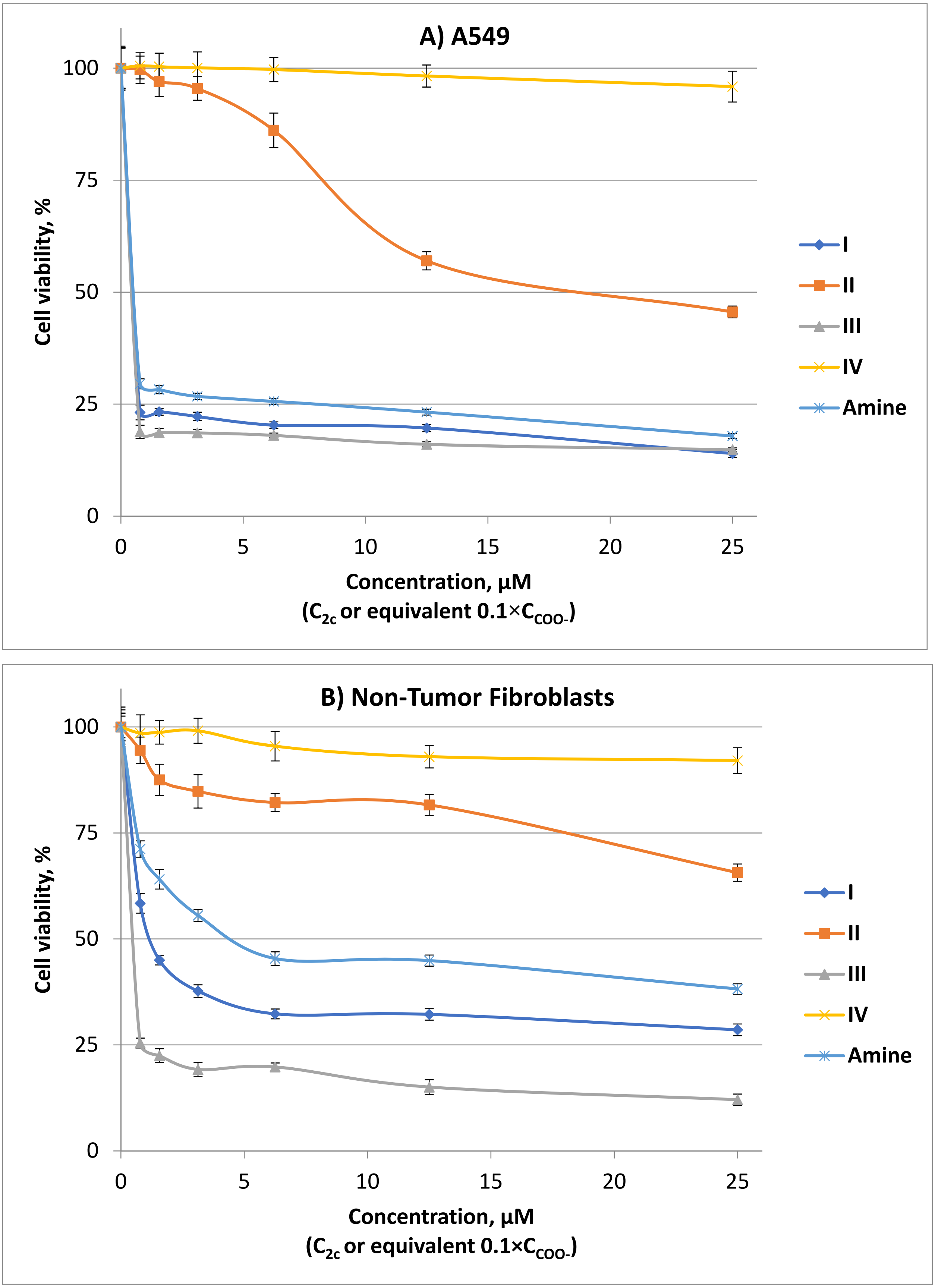

Figure S6. Survival curves of cells after 48 h of drug exposure. MTT-assay, mean normalized % viability values ± SD (n=5). Compounds: **I** – **2c**·HCl–(Alg)–Ca^{2+}; **II** – (Alg)–Ca^{2+}; **III** – **2c**·HCl–(Alg)–Mg^{2+}; **IV** – (Alg)–Mg^{2+}; **Amine** – **2c**. Cell lines: **A** – lung adenocarcinoma A549; **B** – non-tumor fibroblasts.

^{1}H NMR ($CDCl_3$) spectrum of compound **2a**

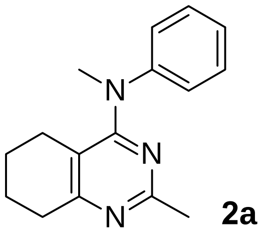

2a

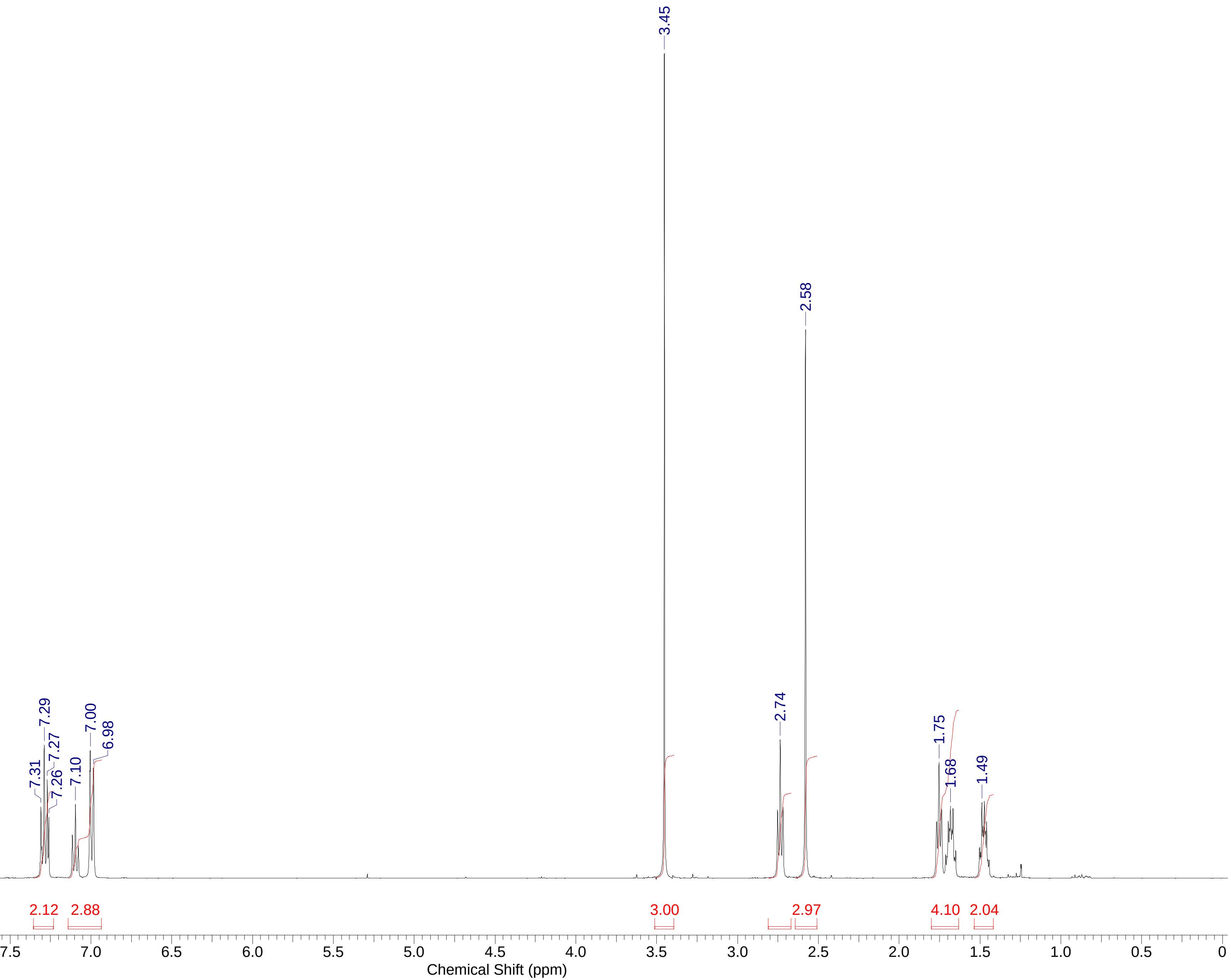

^{13}C NMR ($CDCl_3$) spectrum of compound **2a**

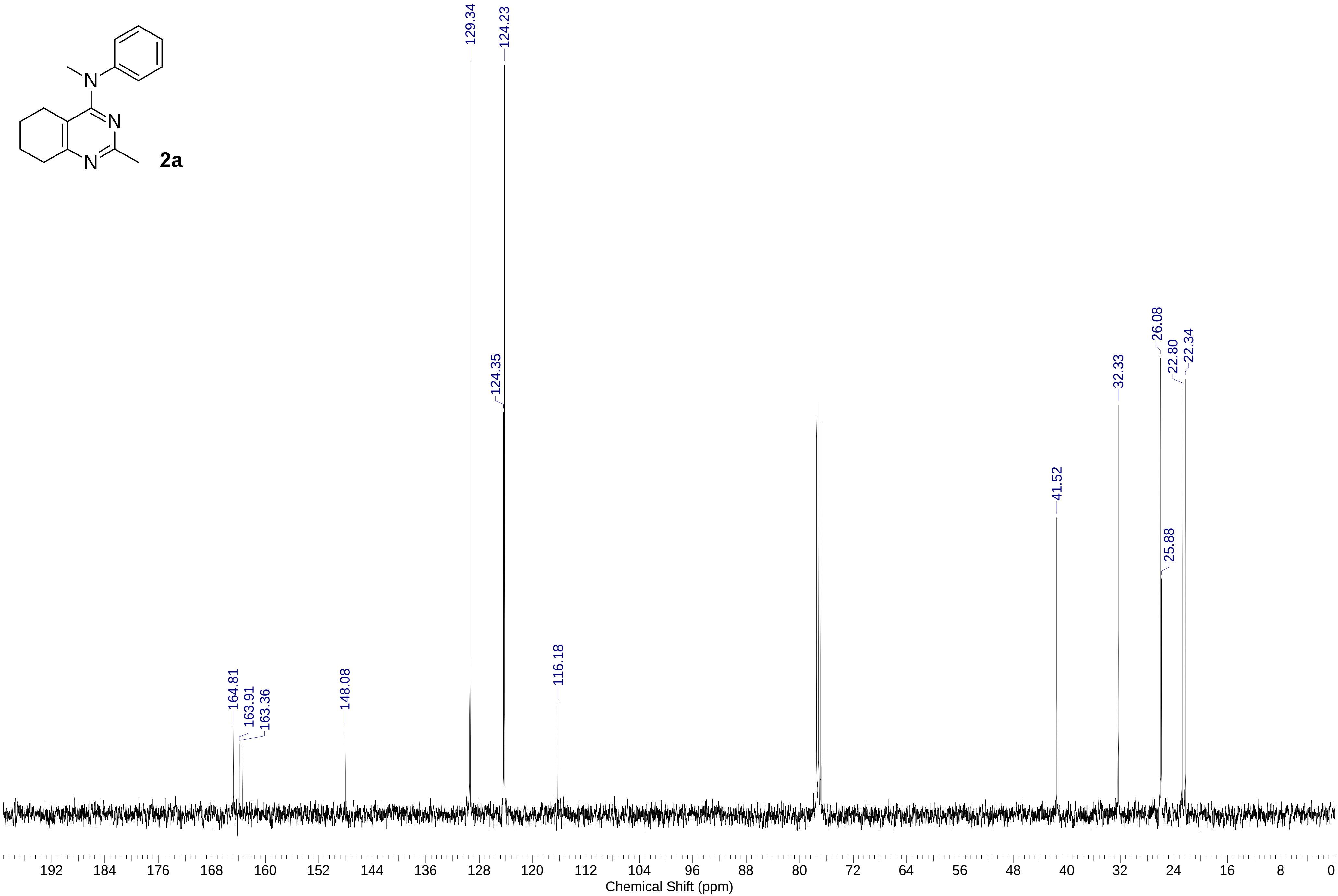

HSQC NMR ($CDCl_3$) spectrum of compound **2a**

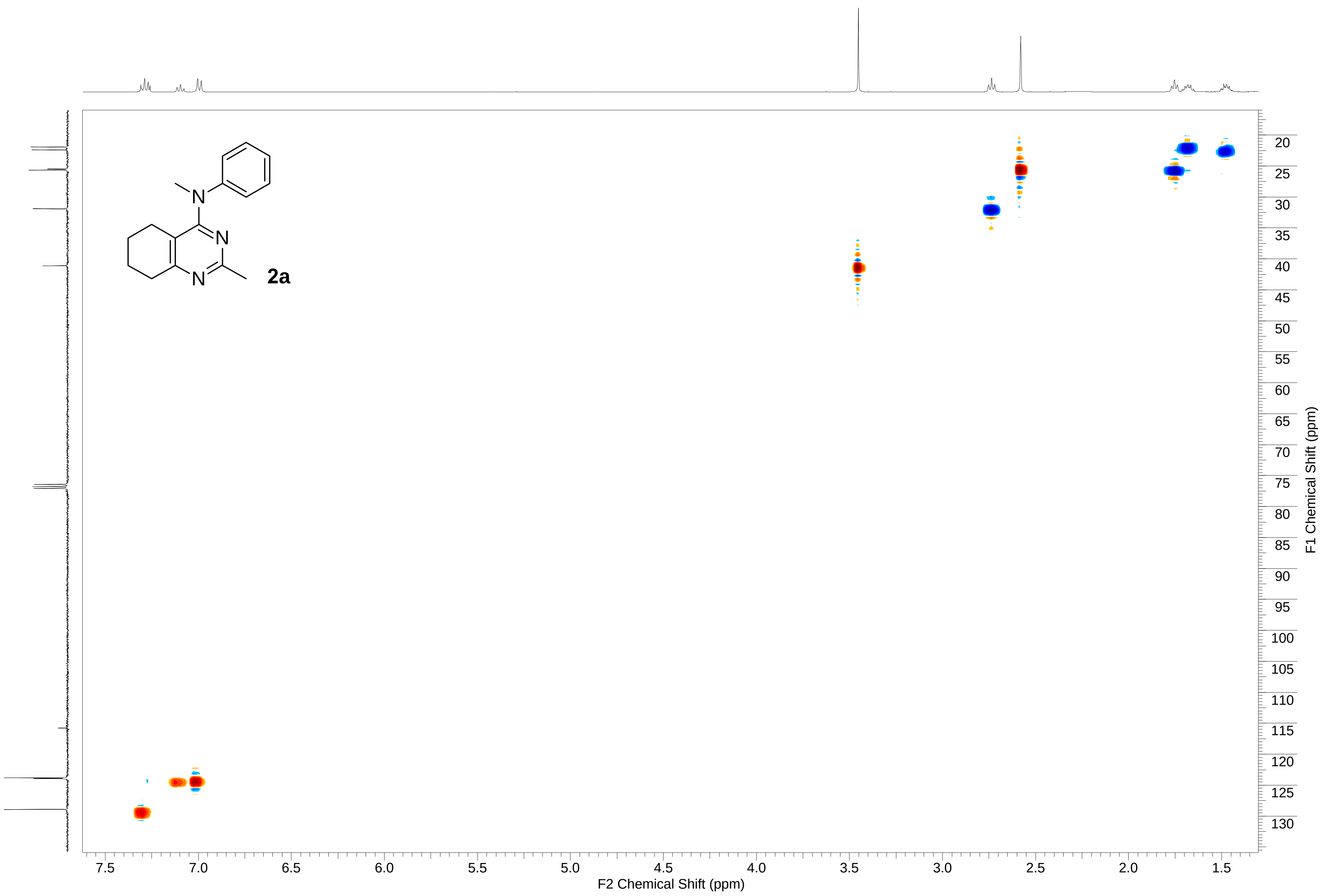

HMBC NMR ($CDCl_3$) spectrum of compound **2a**

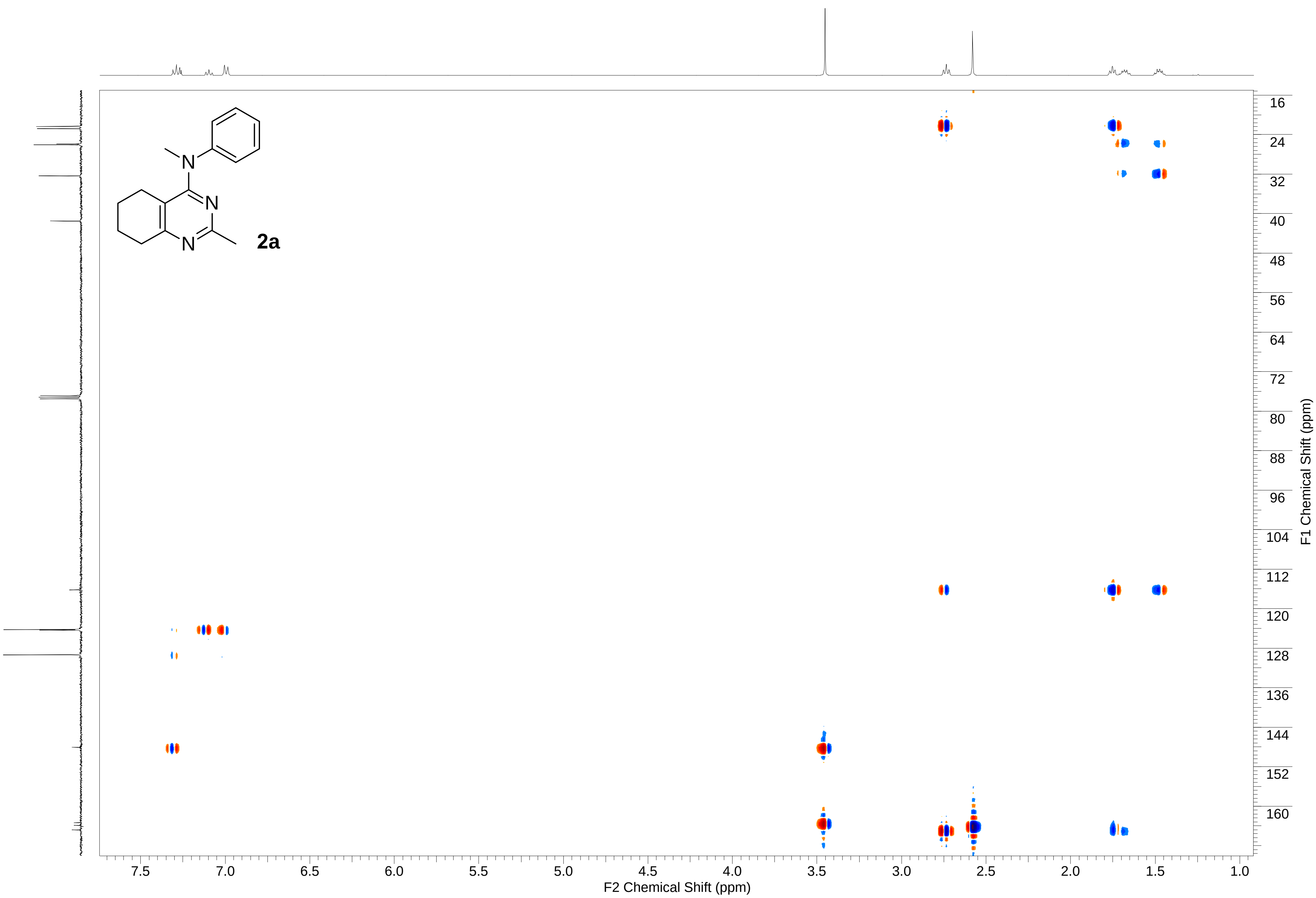

^1H NMR ($CDCl_3$) spectrum of compound **2b**

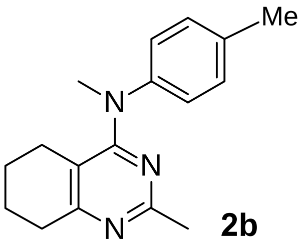

2b

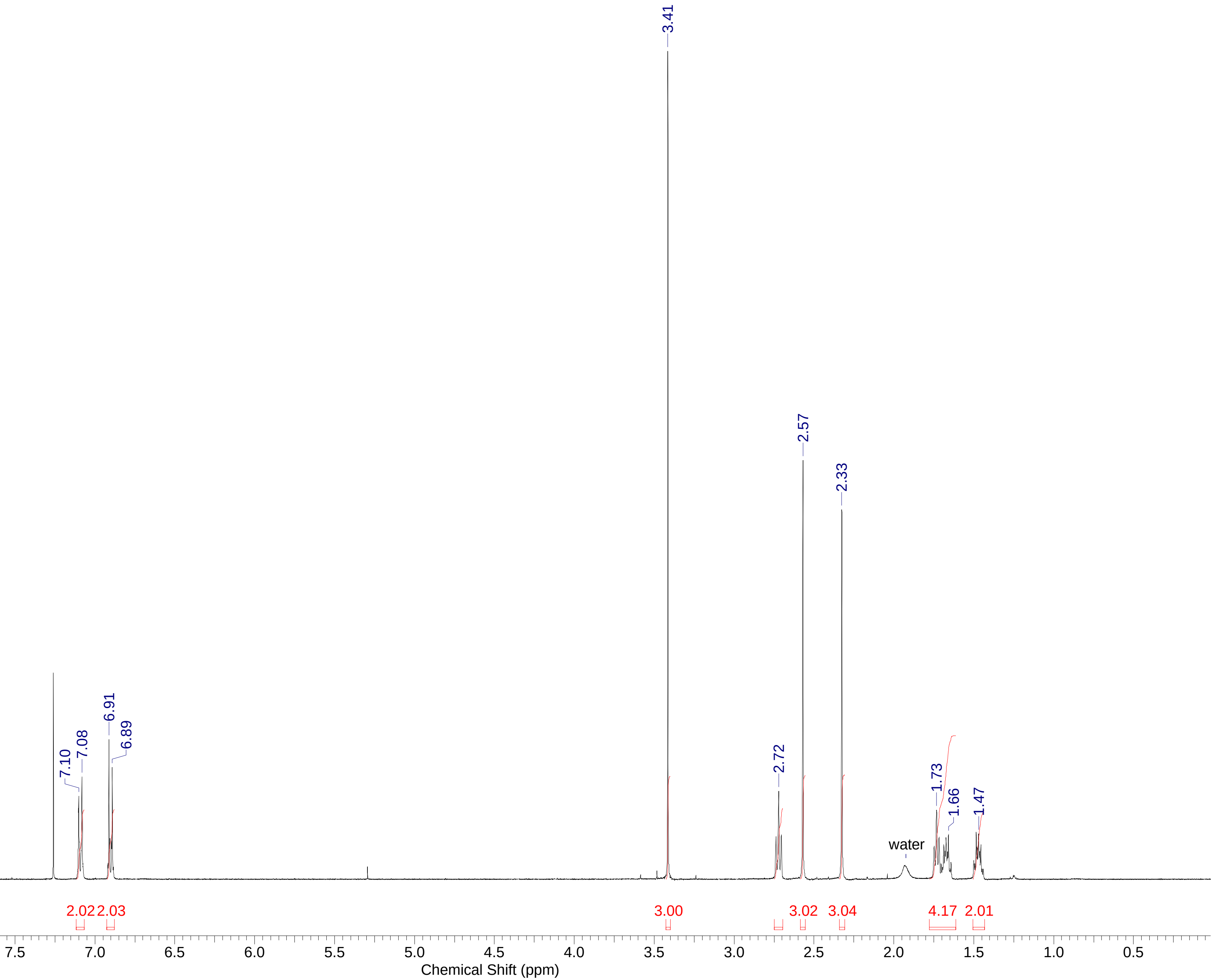

^{13}C NMR (CDCl$_3$) spectrum of compound **2b**

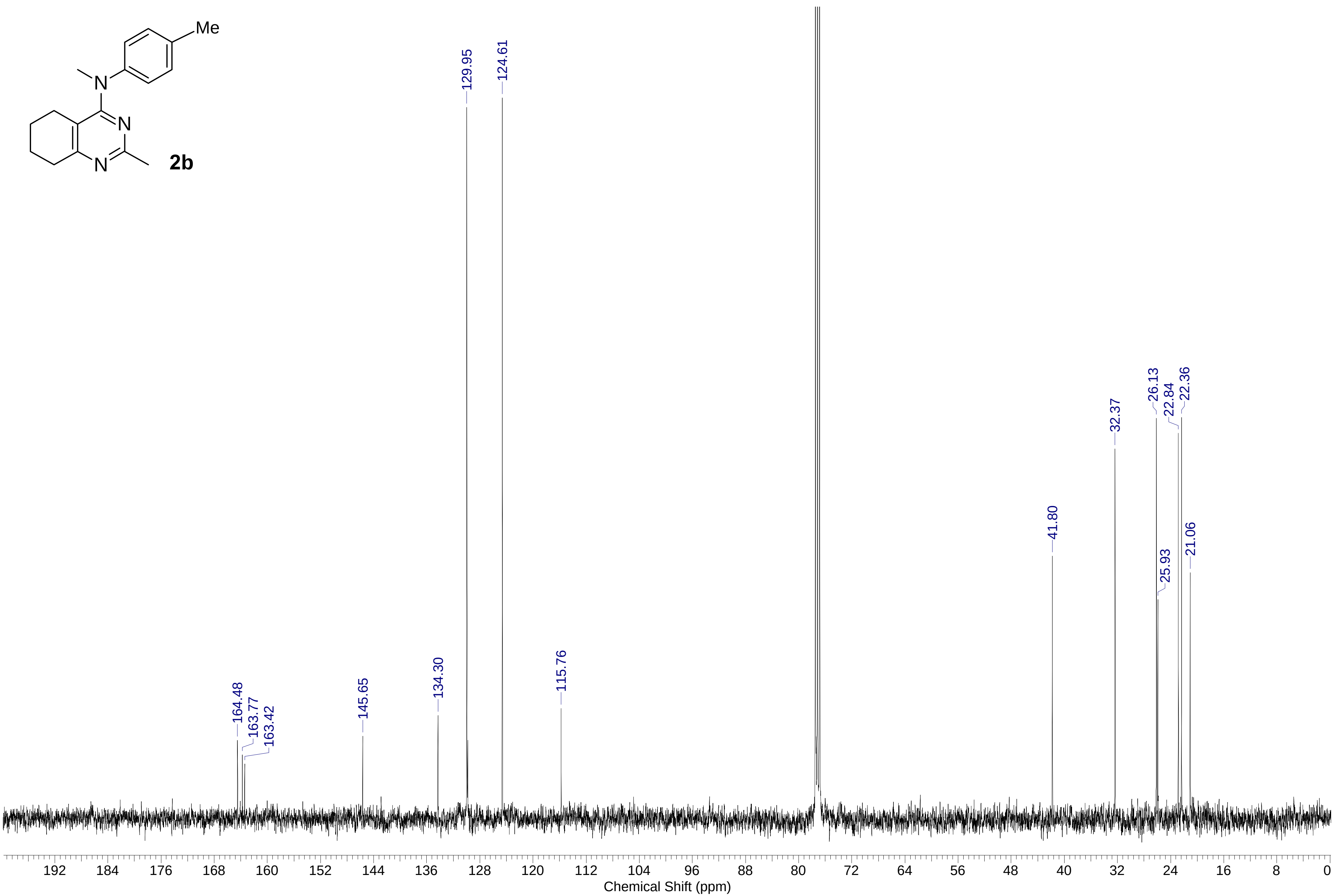

HSQC NMR ($CDCl_3$) spectrum of compound **2b**

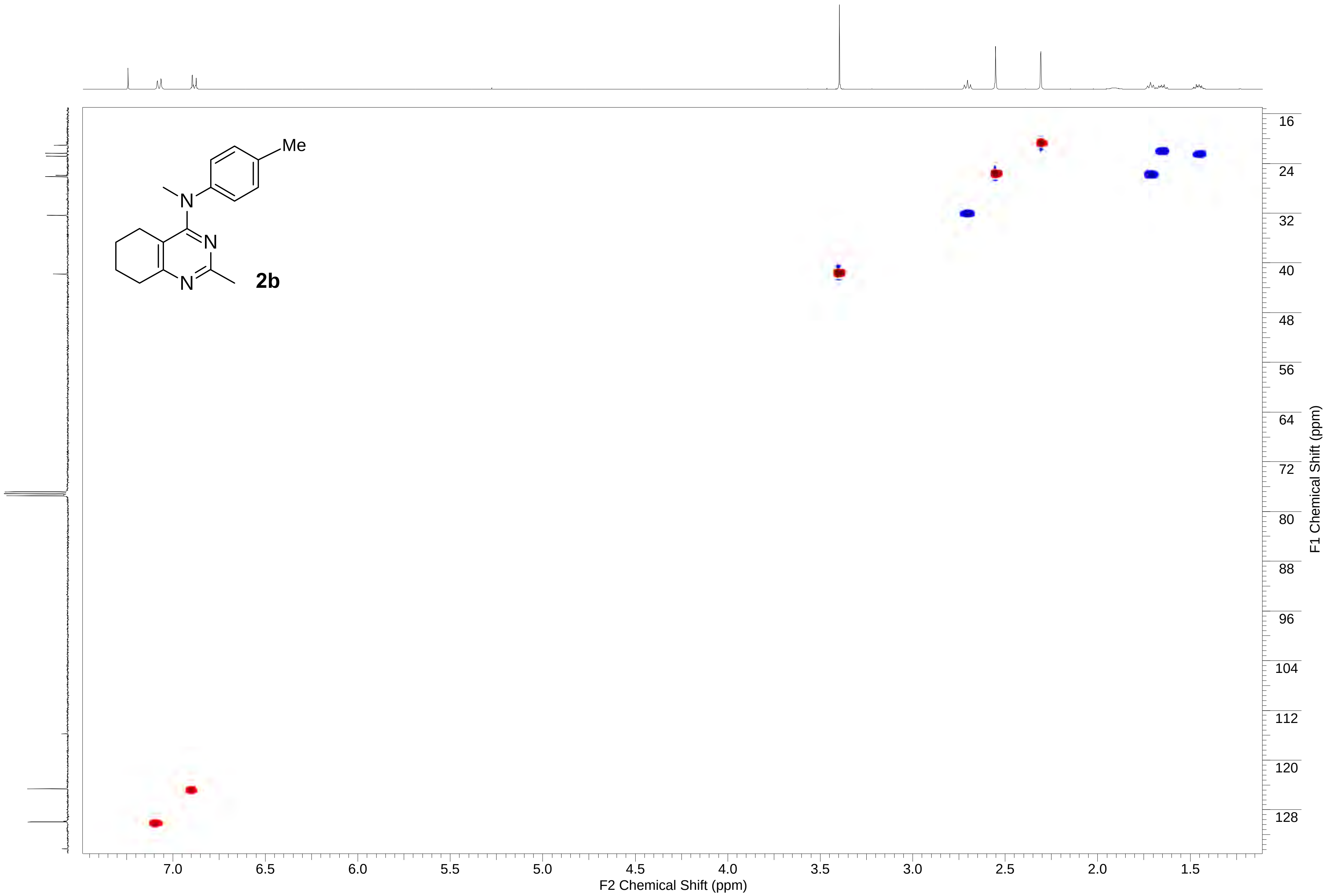

HMBC NMR ($CDCl_3$) spectrum of compound **2b**

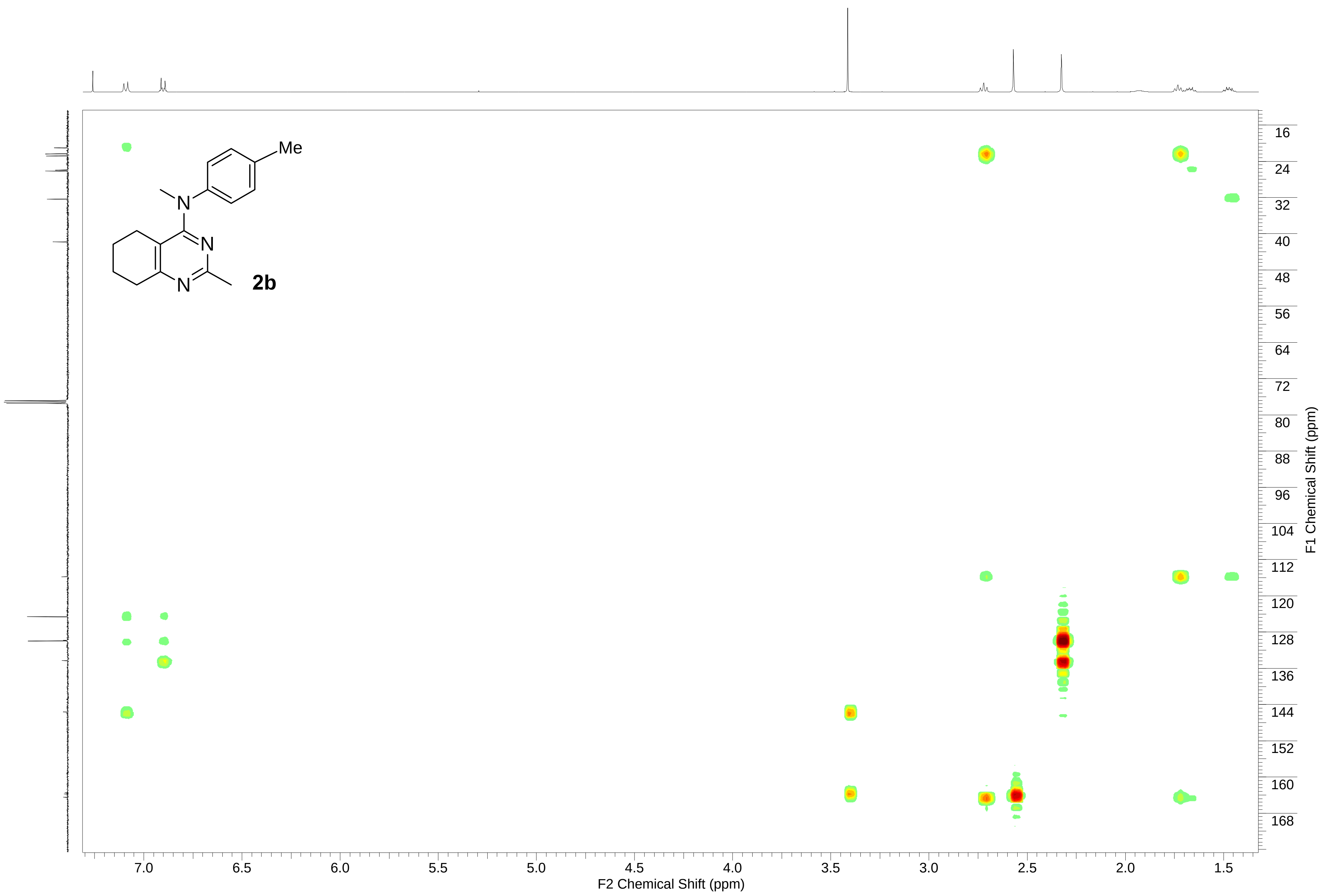

^{1}H NMR ($CDCl_3$) spectrum of compound **2c**

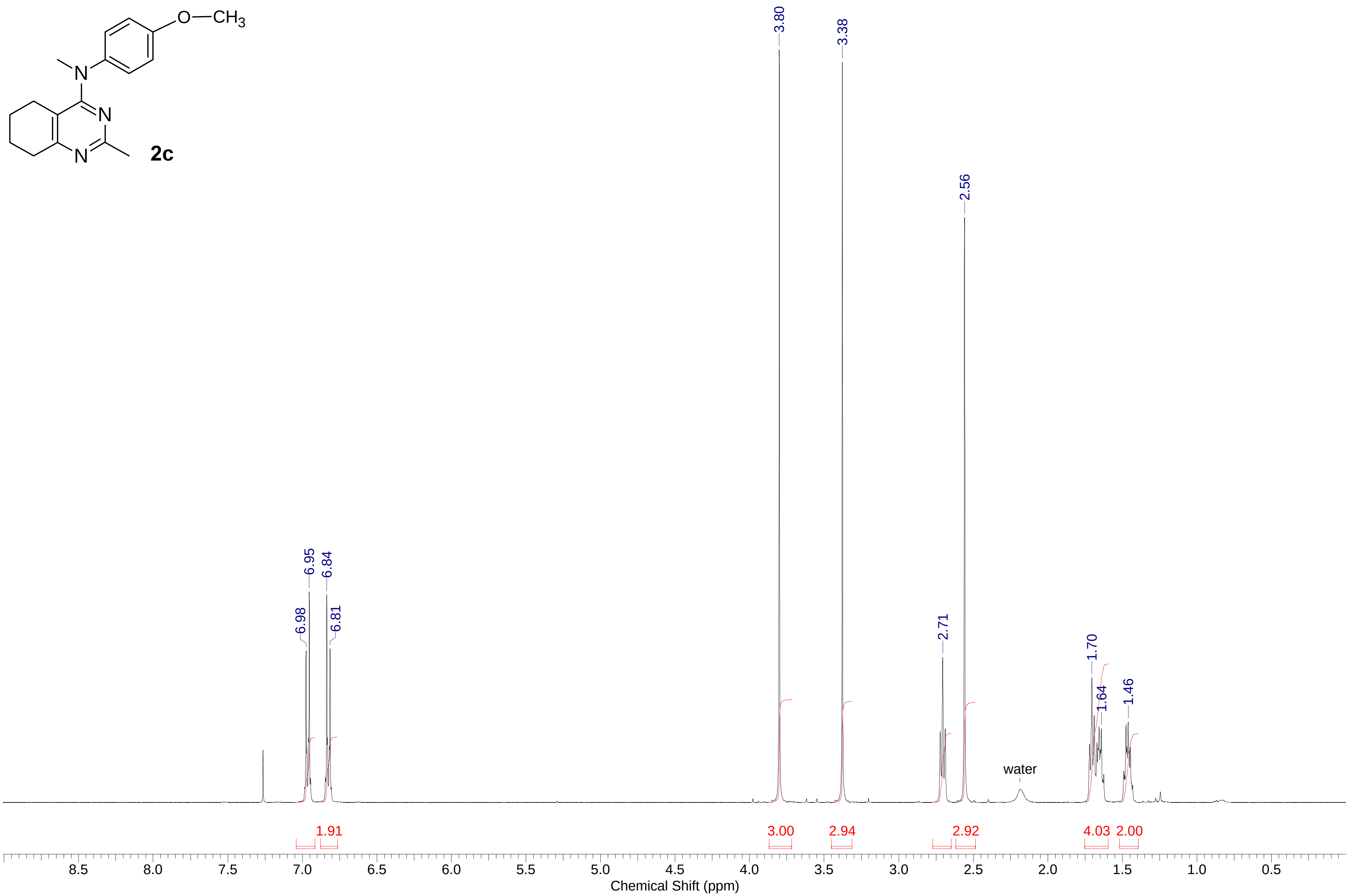

^{13}C NMR ($CDCl_3$) spectrum of compound **2c**

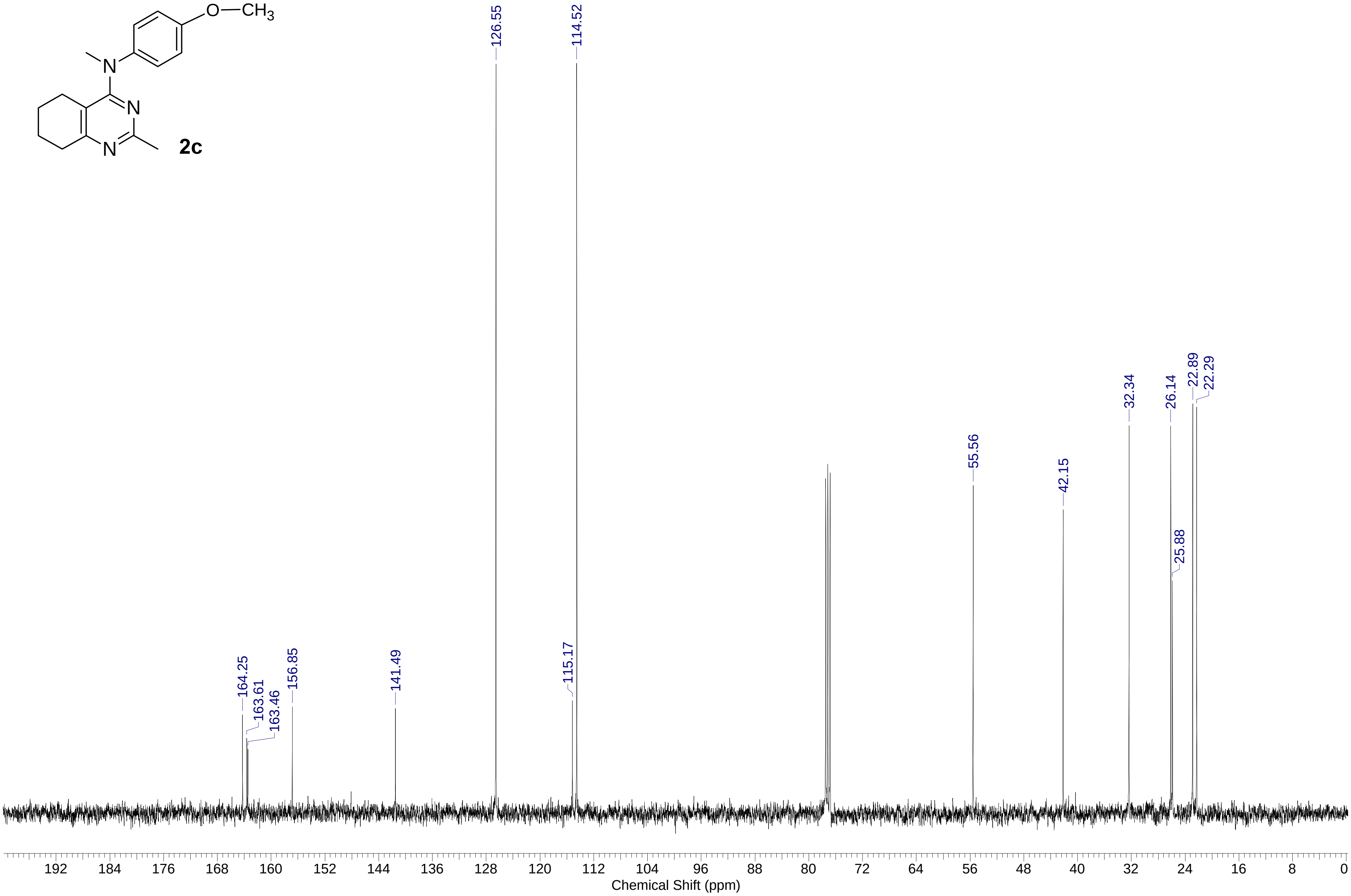

HSQC NMR ($CDCl_3$) spectrum of compound **2c**

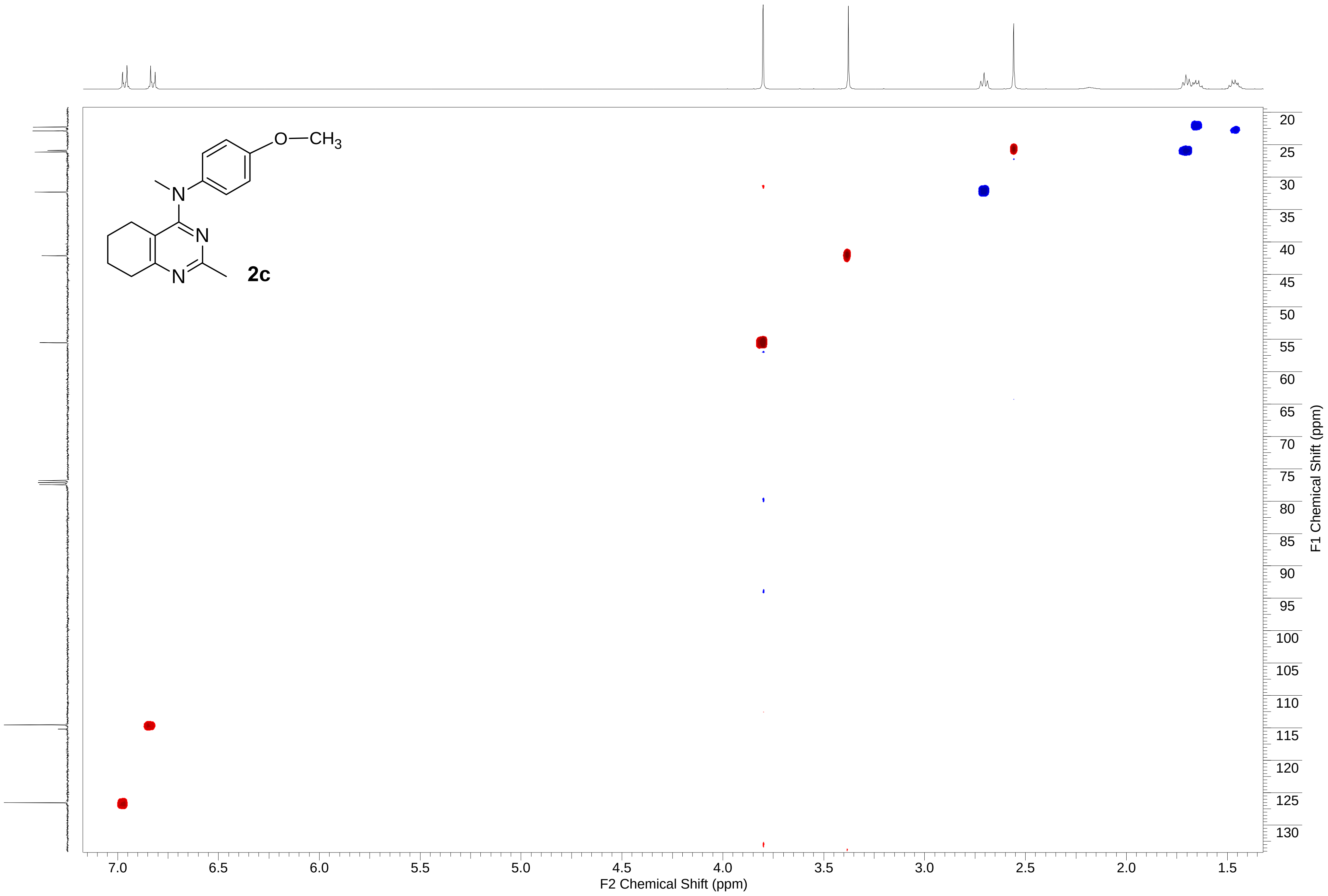

HMBC NMR ($CDCl_3$) spectrum of compound **2c**

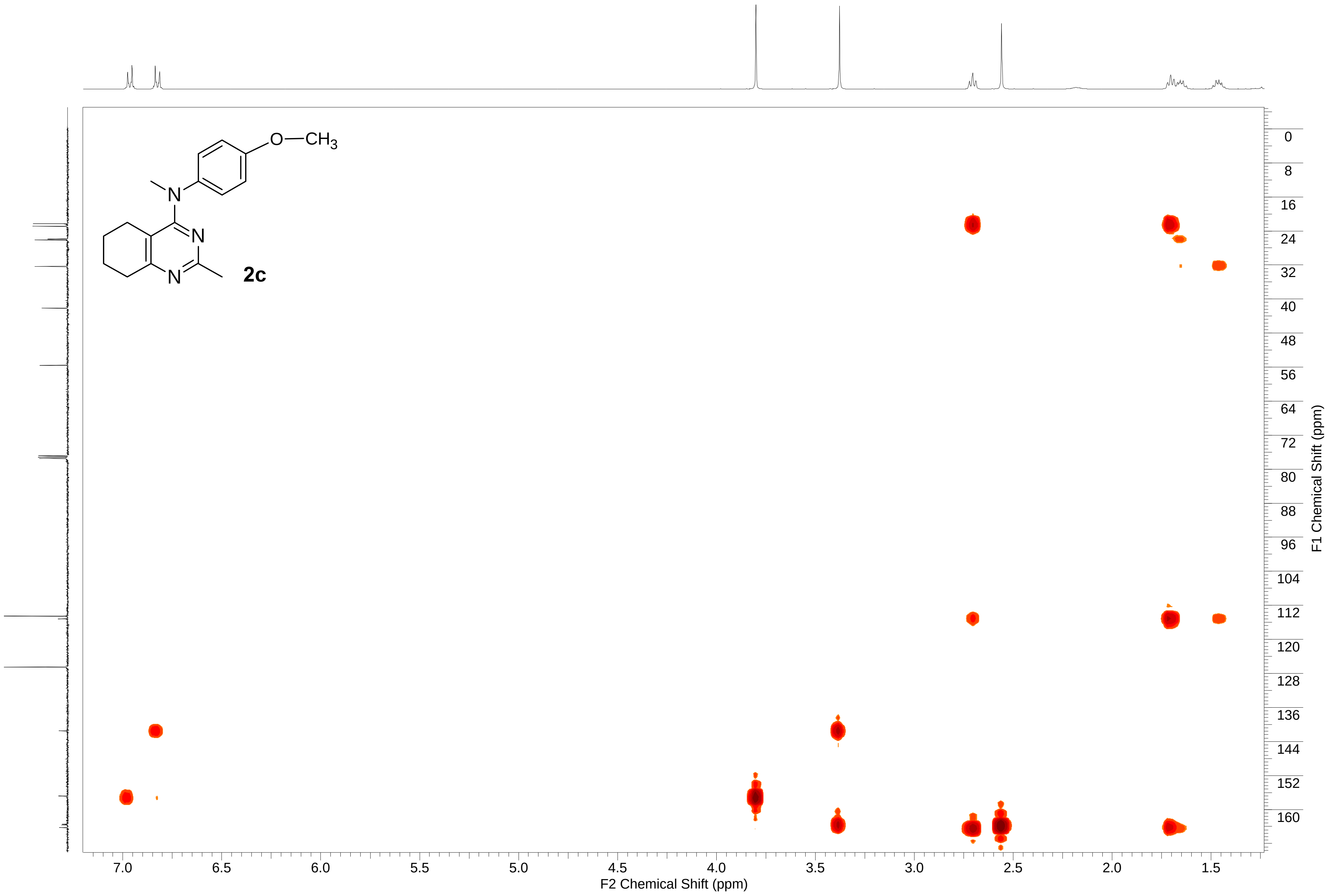

^{1}H NMR ($CDCl_3$) spectrum of compound **2d**

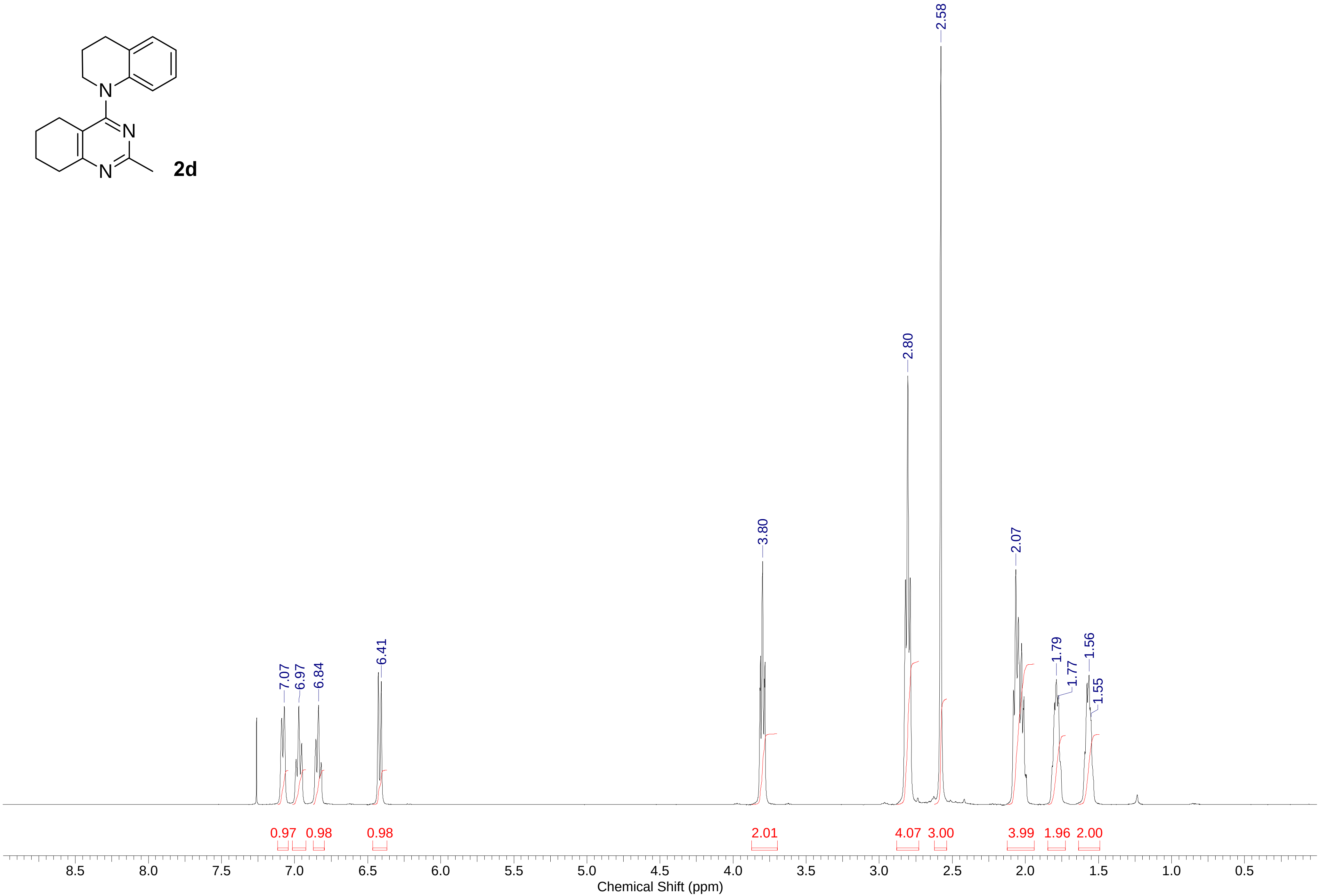

^{13}C NMR ($CDCl_3$) spectrum of compound **2d**

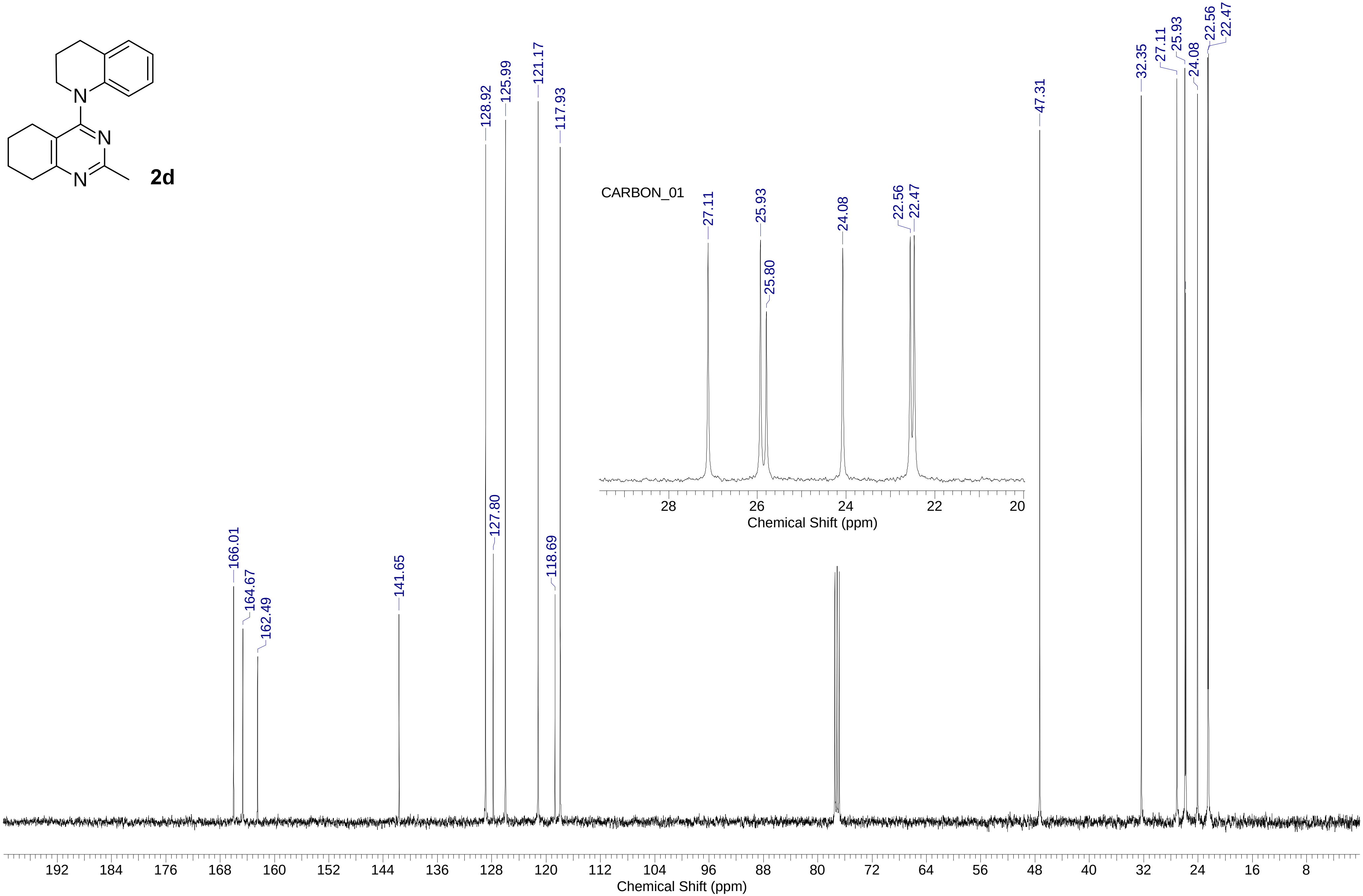

HSQC NMR ($CDCl_3$) spectrum of compound **2d**

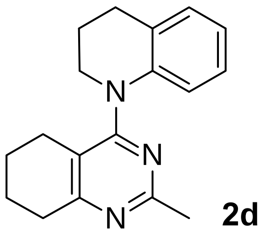

2d

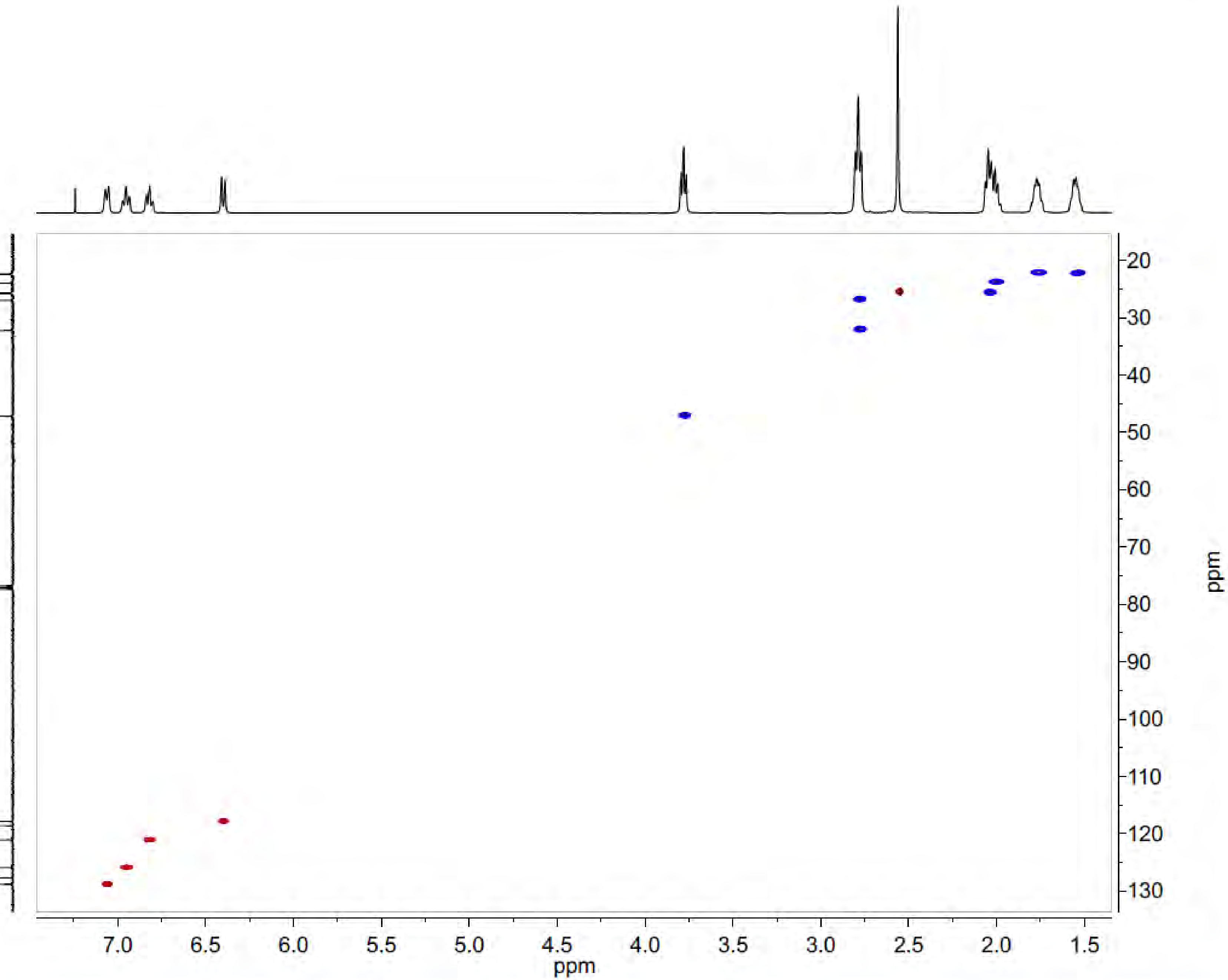

HMBC NMR ($CDCl_3$) spectrum of compound **2d**

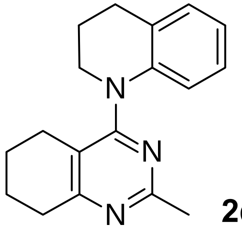

2d

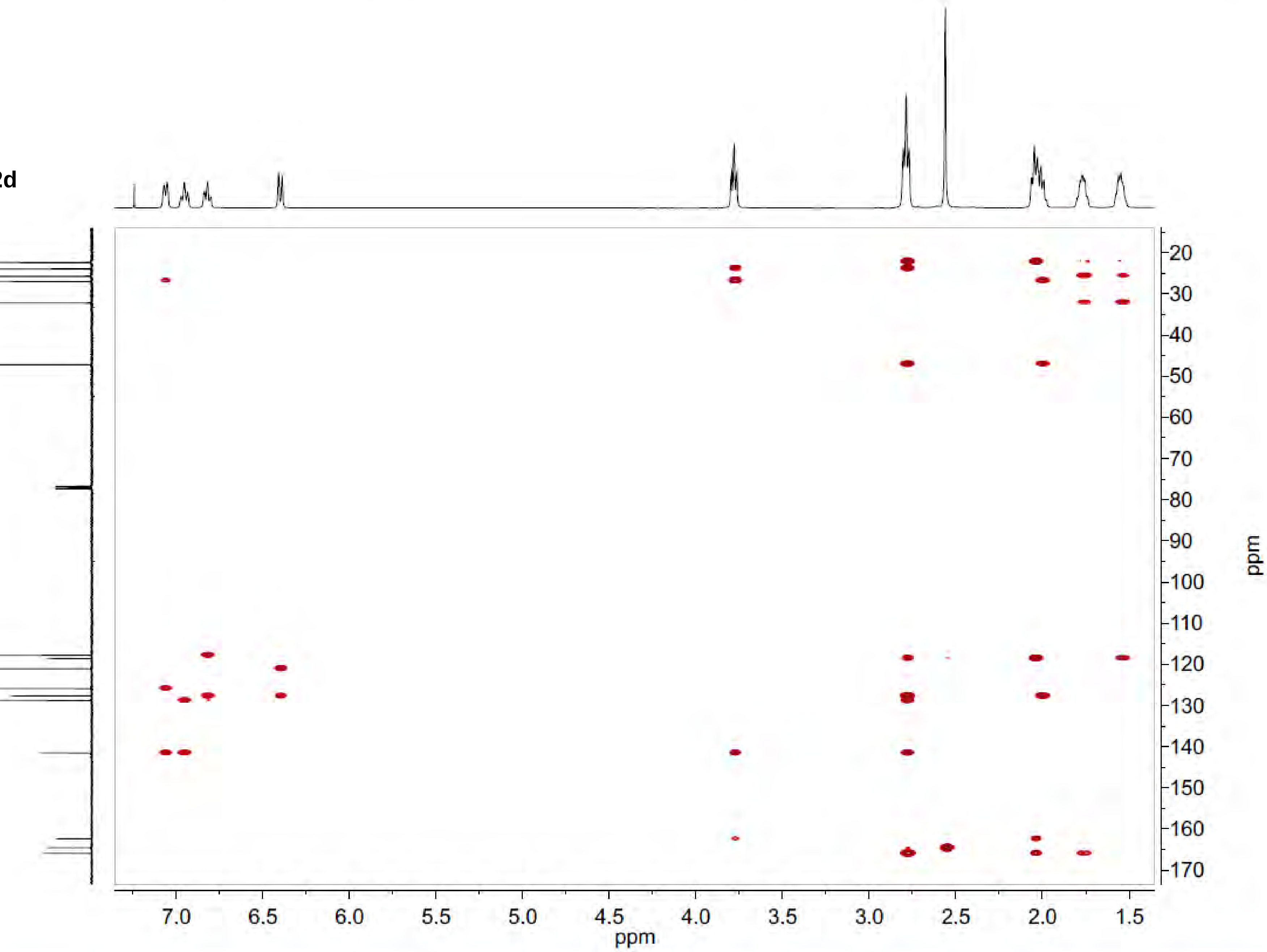

^{1}H NMR ($CDCl_3$) spectrum of compound **2e**

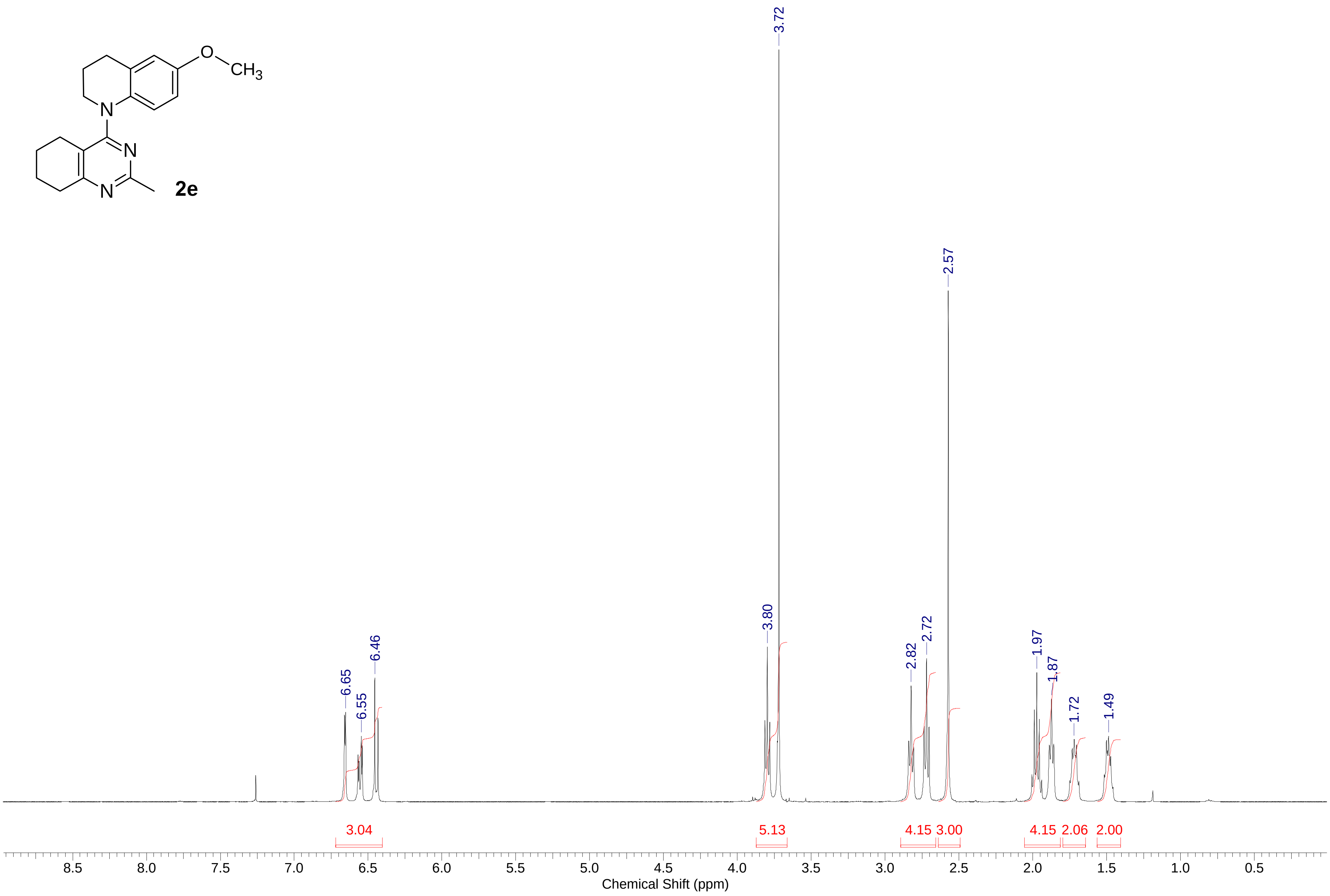

^{13}C NMR ($CDCl_3$) spectrum of compound **2e**

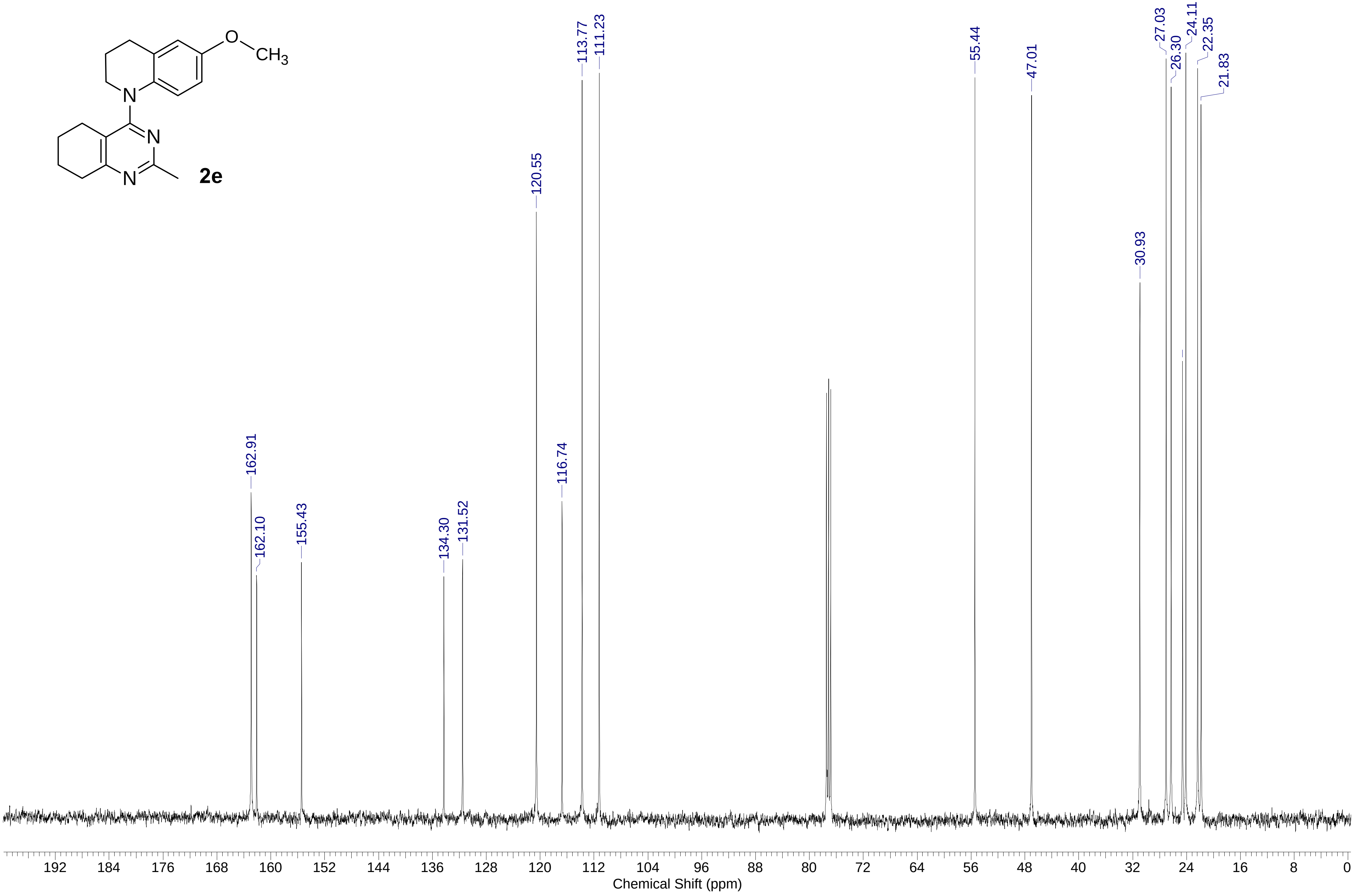

HSQC NMR ($CDCl_3$) spectrum of compound **2e**

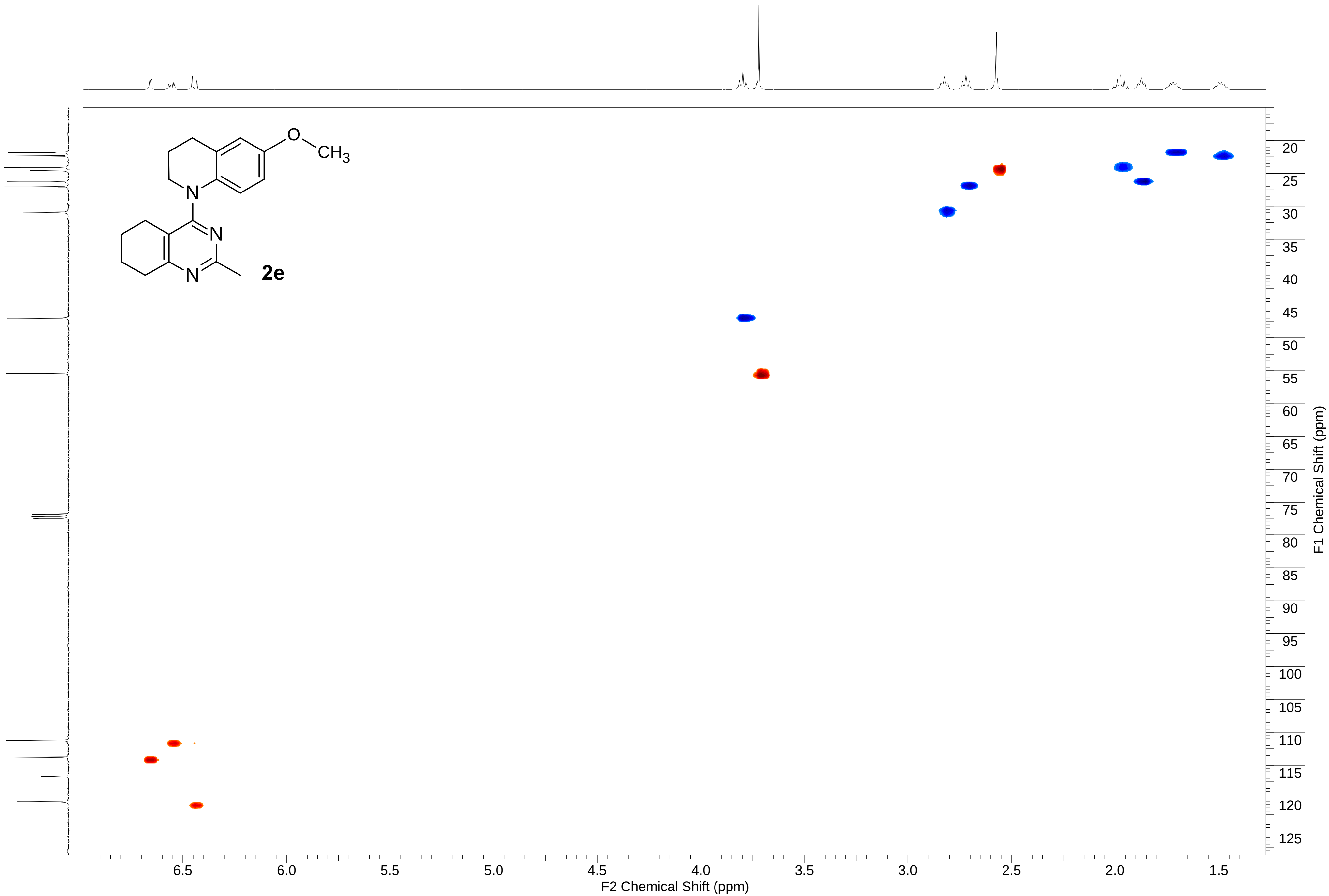

HMBC NMR ($CDCl_3$) spectrum of compound **2e**

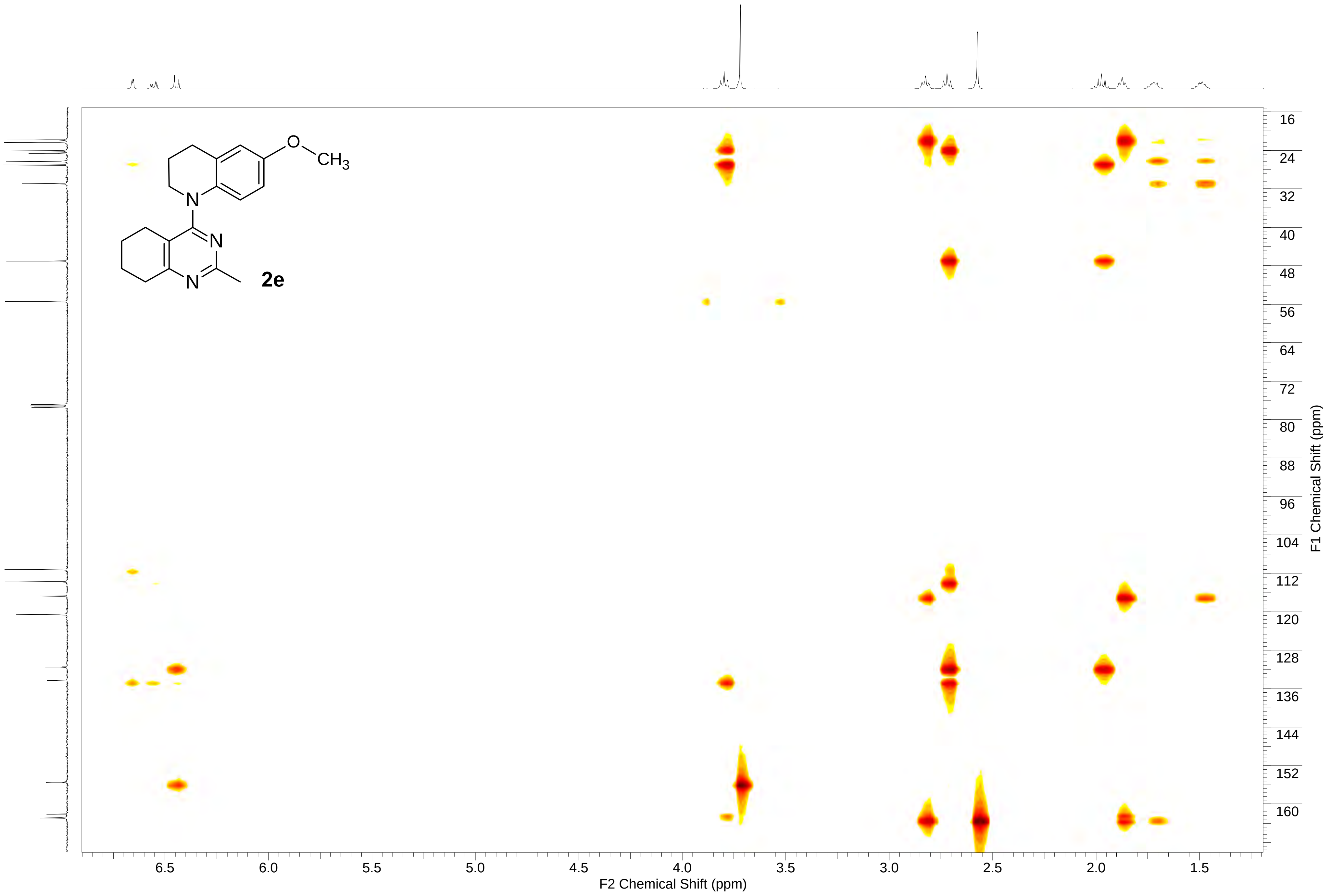

^1H NMR ($CDCl_3$) spectrum of compound **2f**

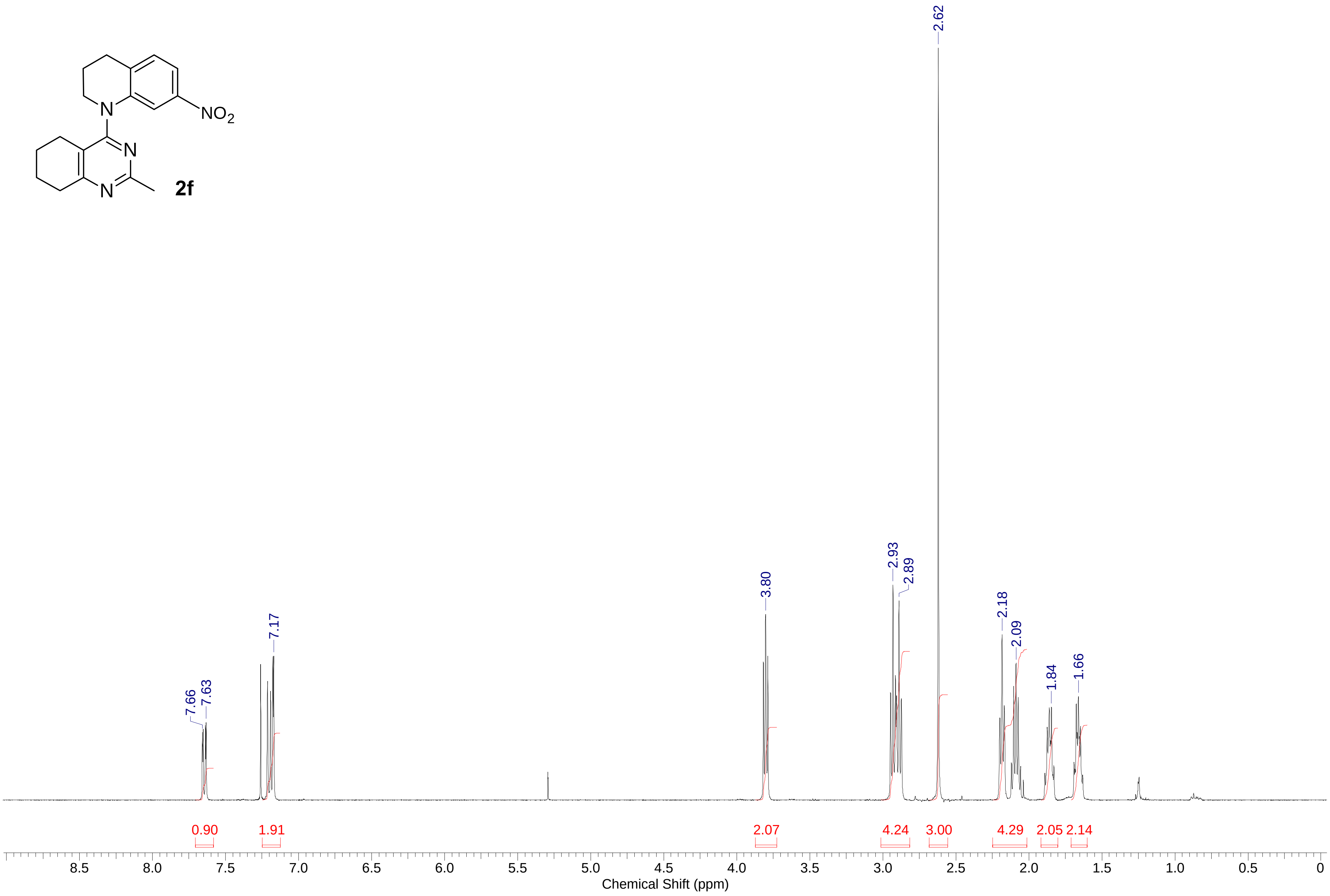

^{13}C NMR ($CDCl_3$) spectrum of compound **2f**

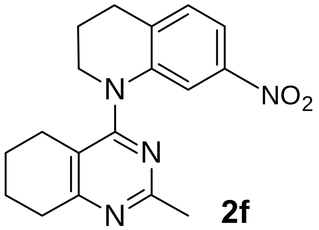

2f

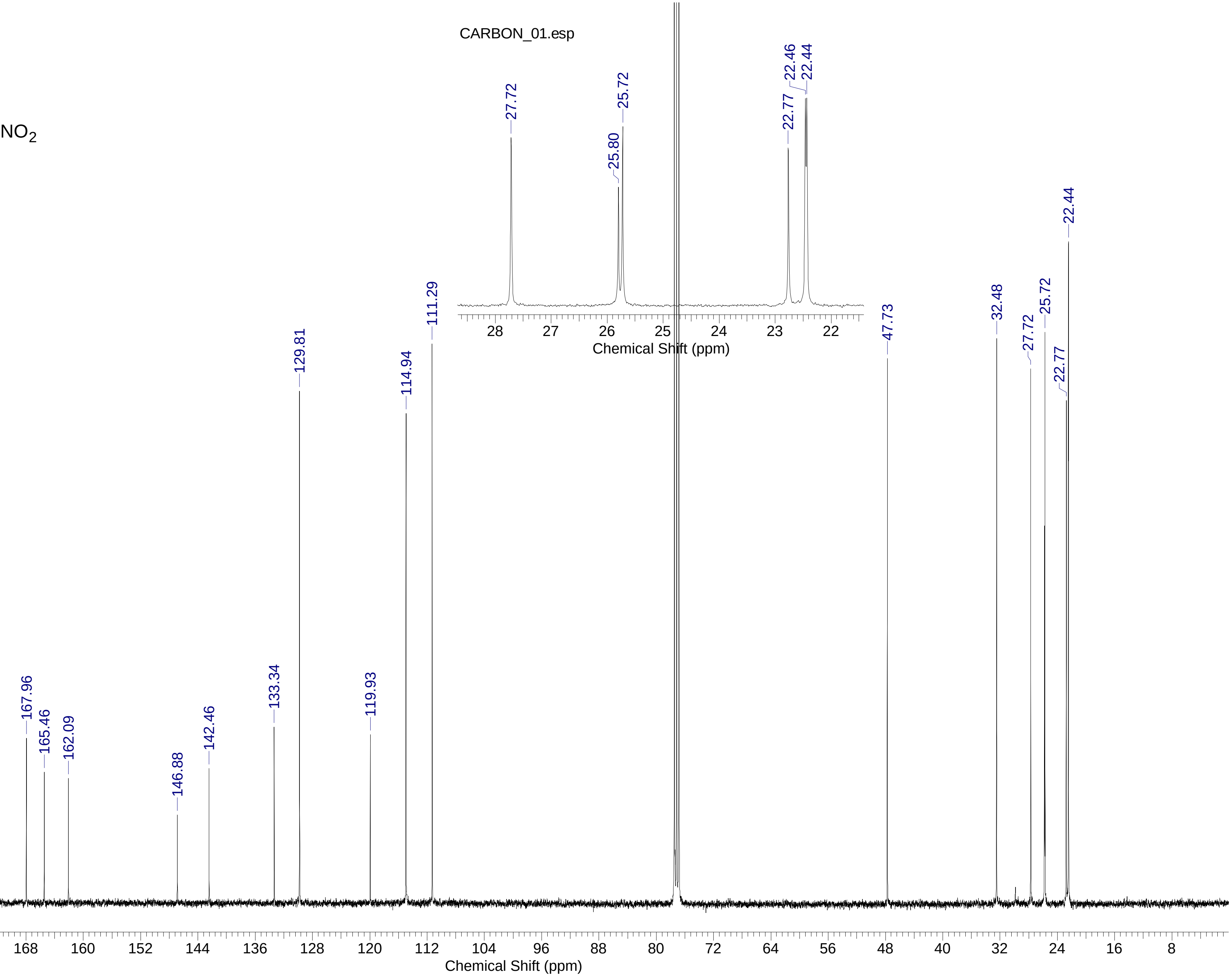

HSQC NMR ($CDCl_3$) spectrum of compound **2f**

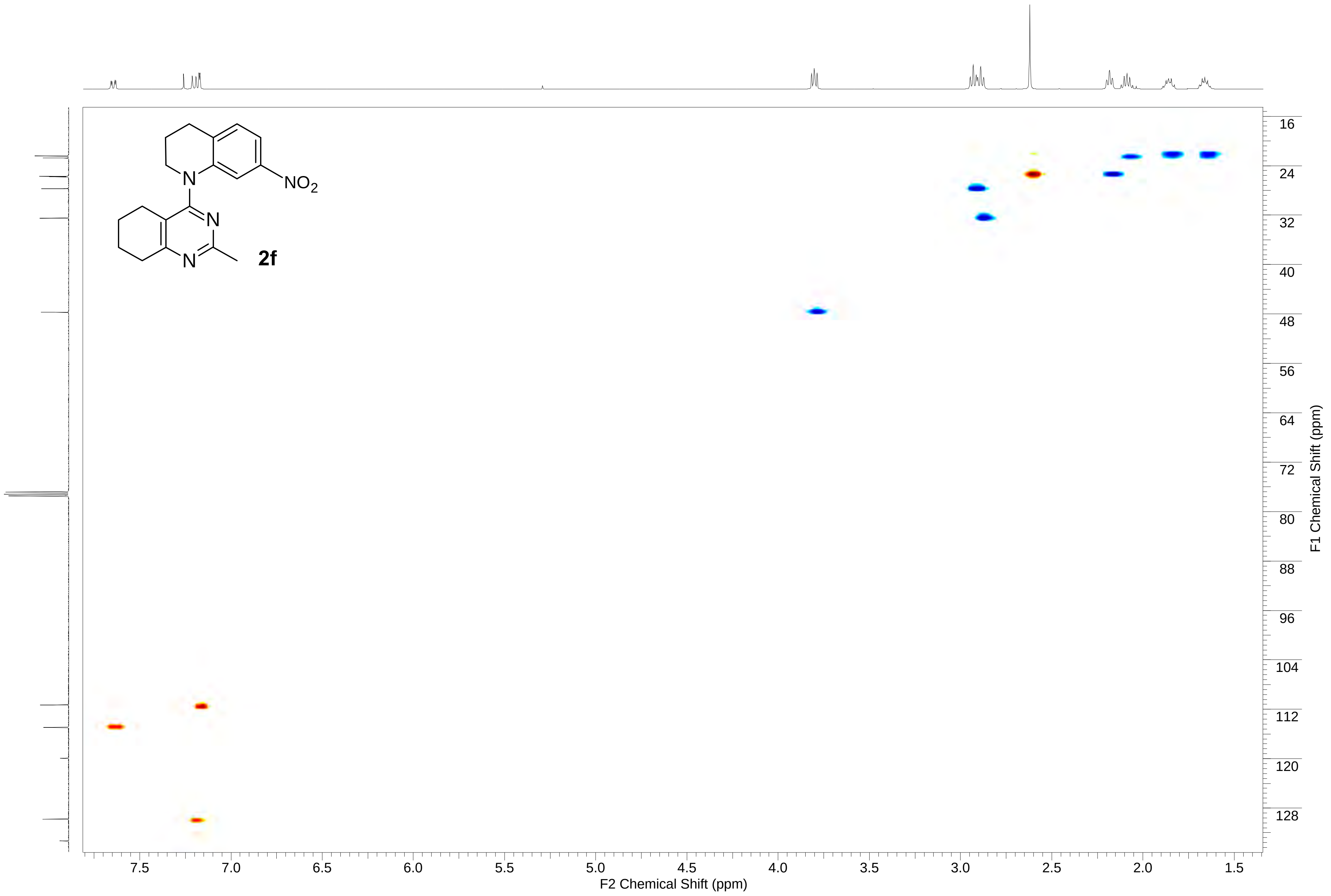

HMBC NMR ($CDCl_3$) spectrum of compound **2f**

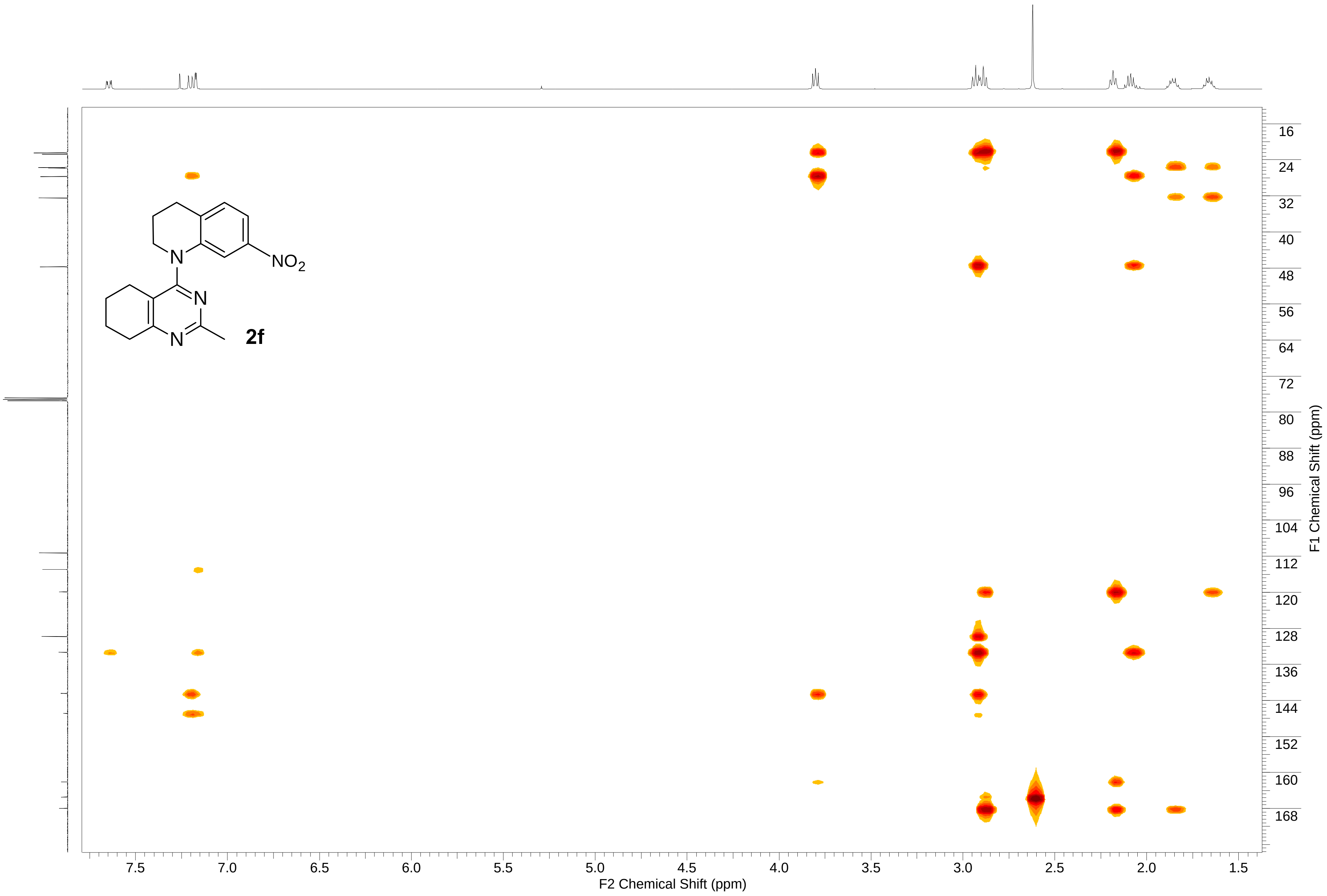

^{1}H NMR ($CDCl_3$) spectrum of compound **2g**

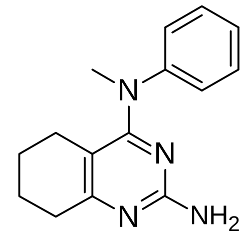

2g

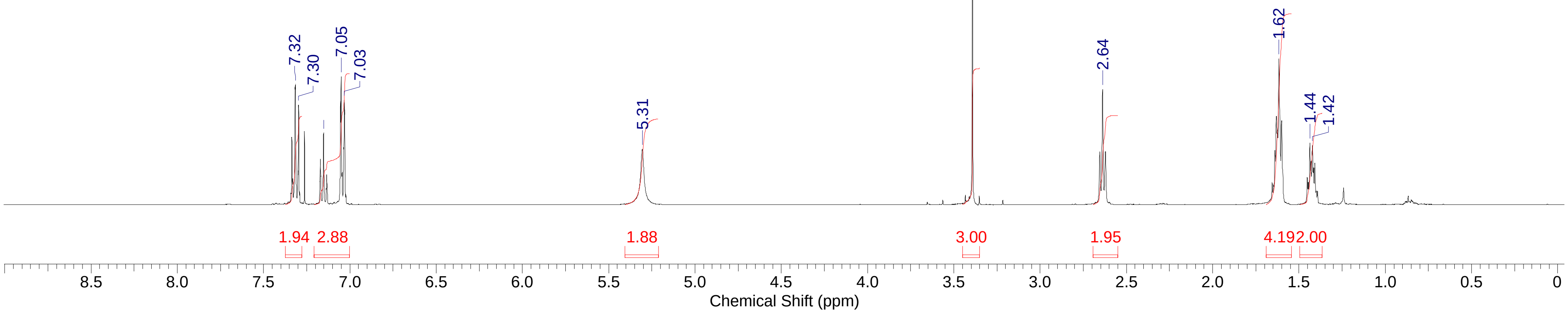

^{13}C NMR ($CDCl_3$) spectrum of compound **2g**

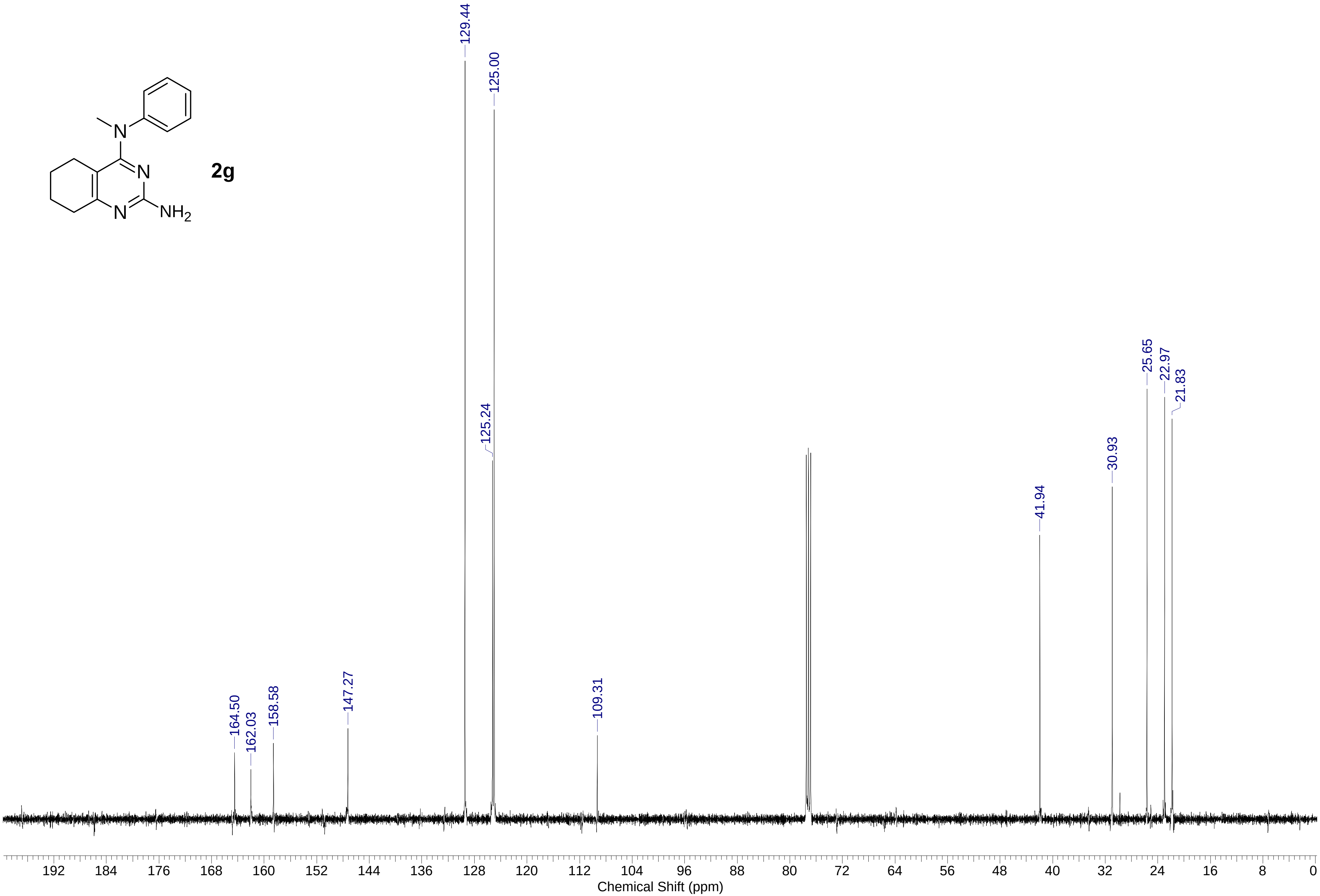

HSQC NMR ($CDCl_3$) spectrum of compound **2g**

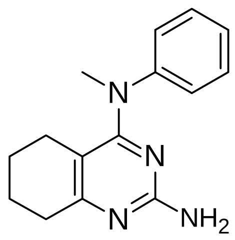

2g

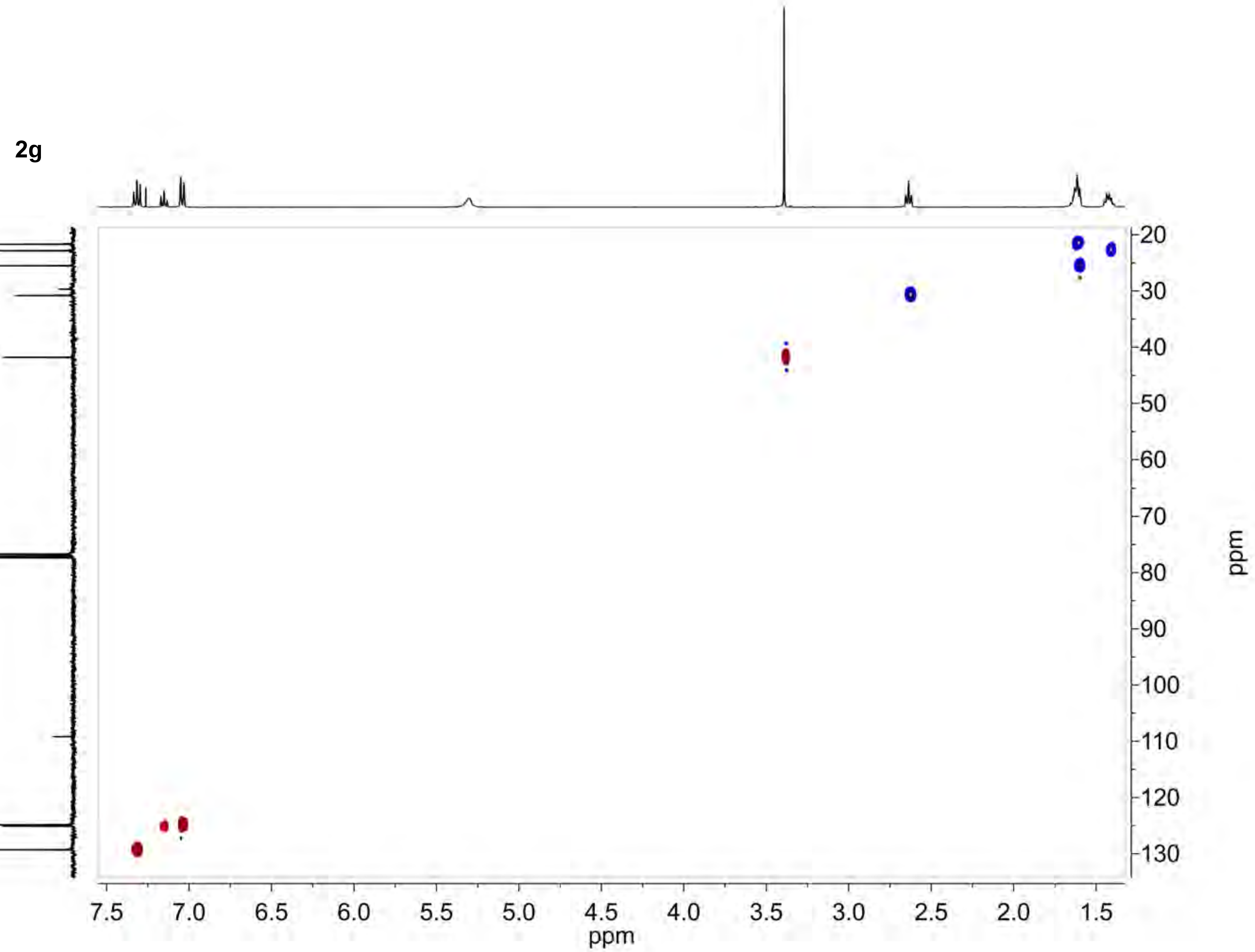

HMBC NMR ($CDCl_3$) spectrum of compound **2g**

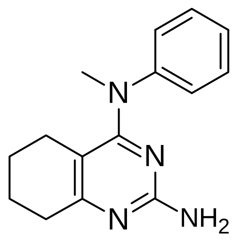

2g

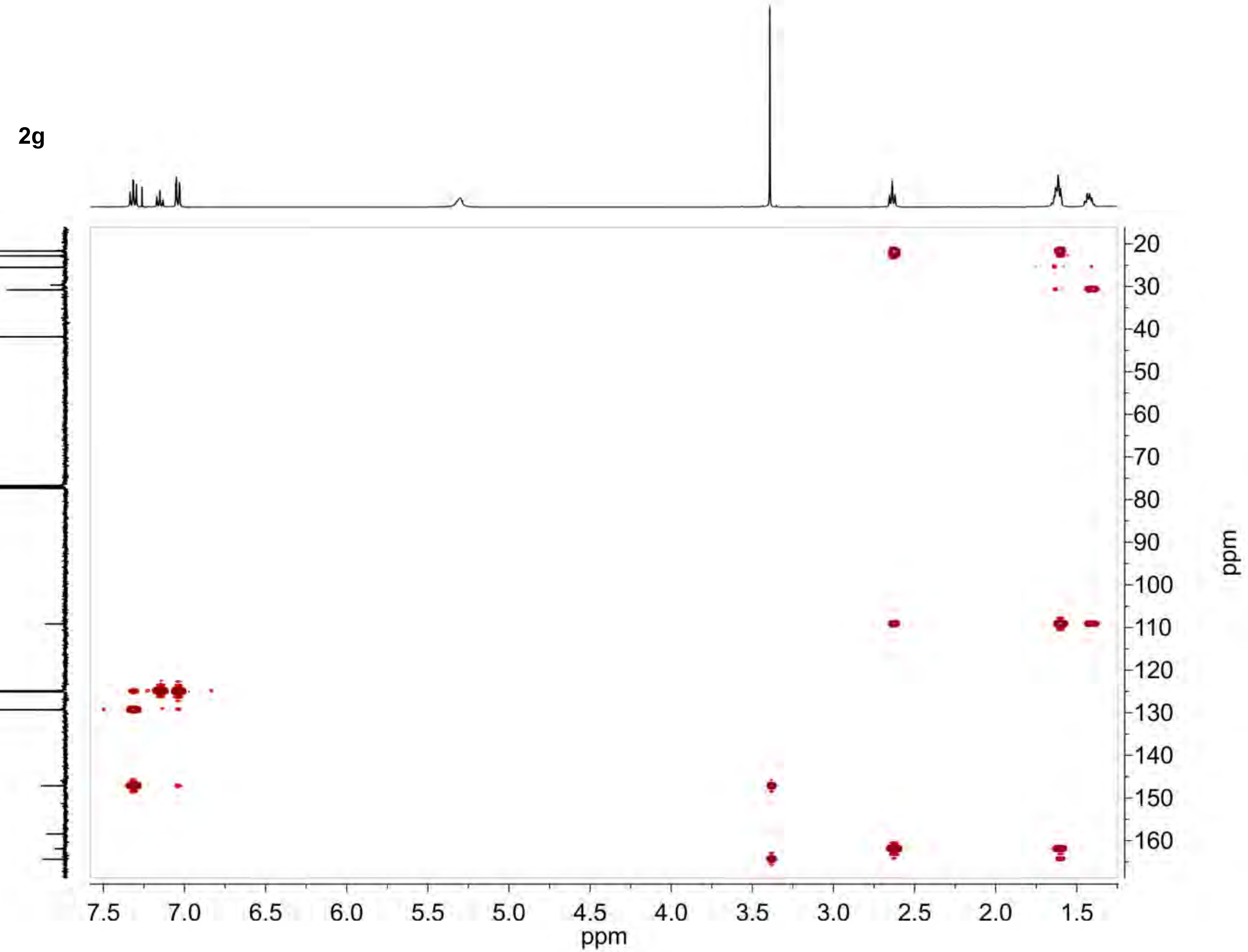

^{1}H NMR ($CDCl_3$) spectrum of compound **2h**

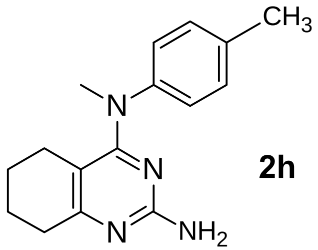

2h

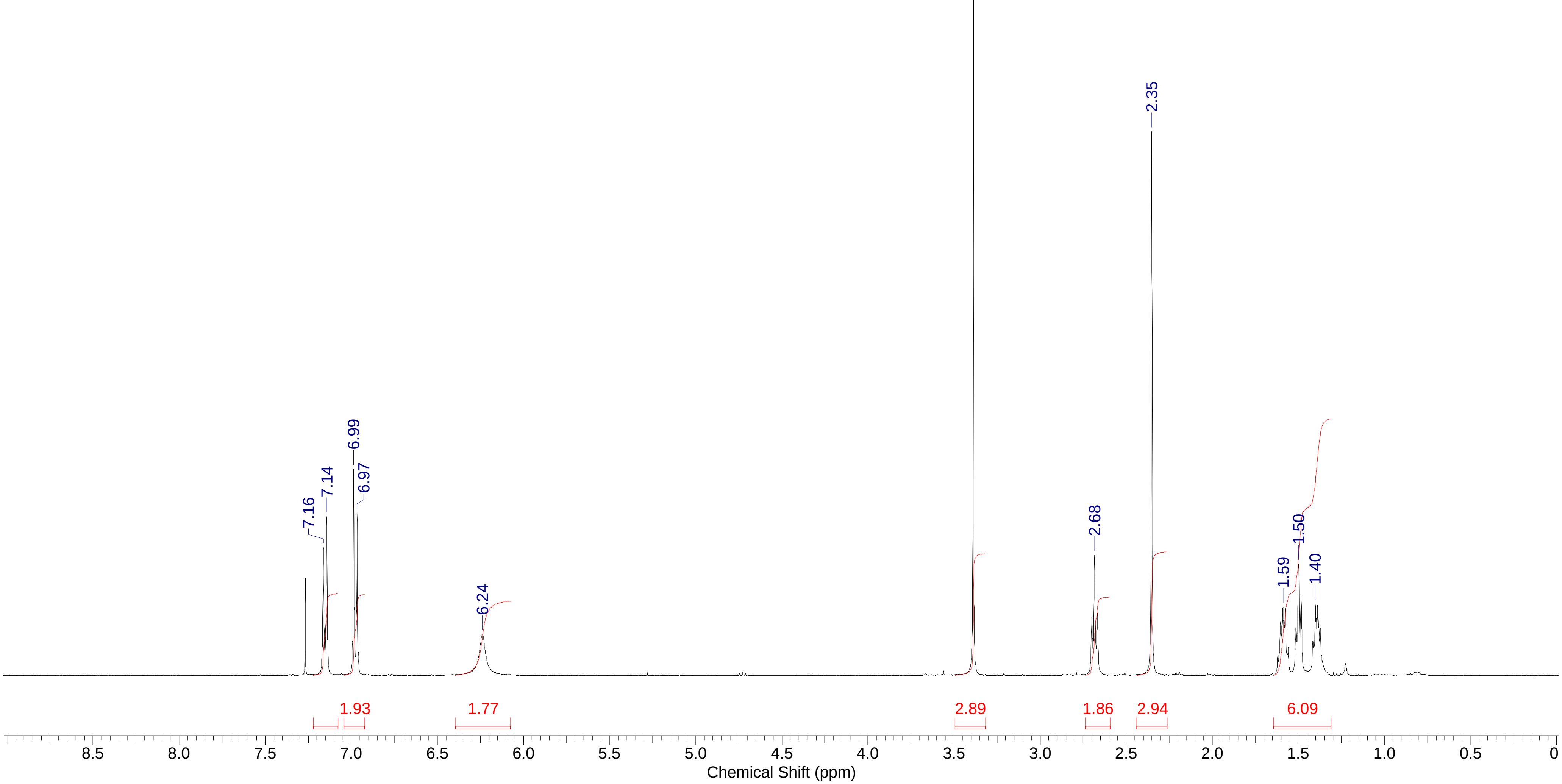

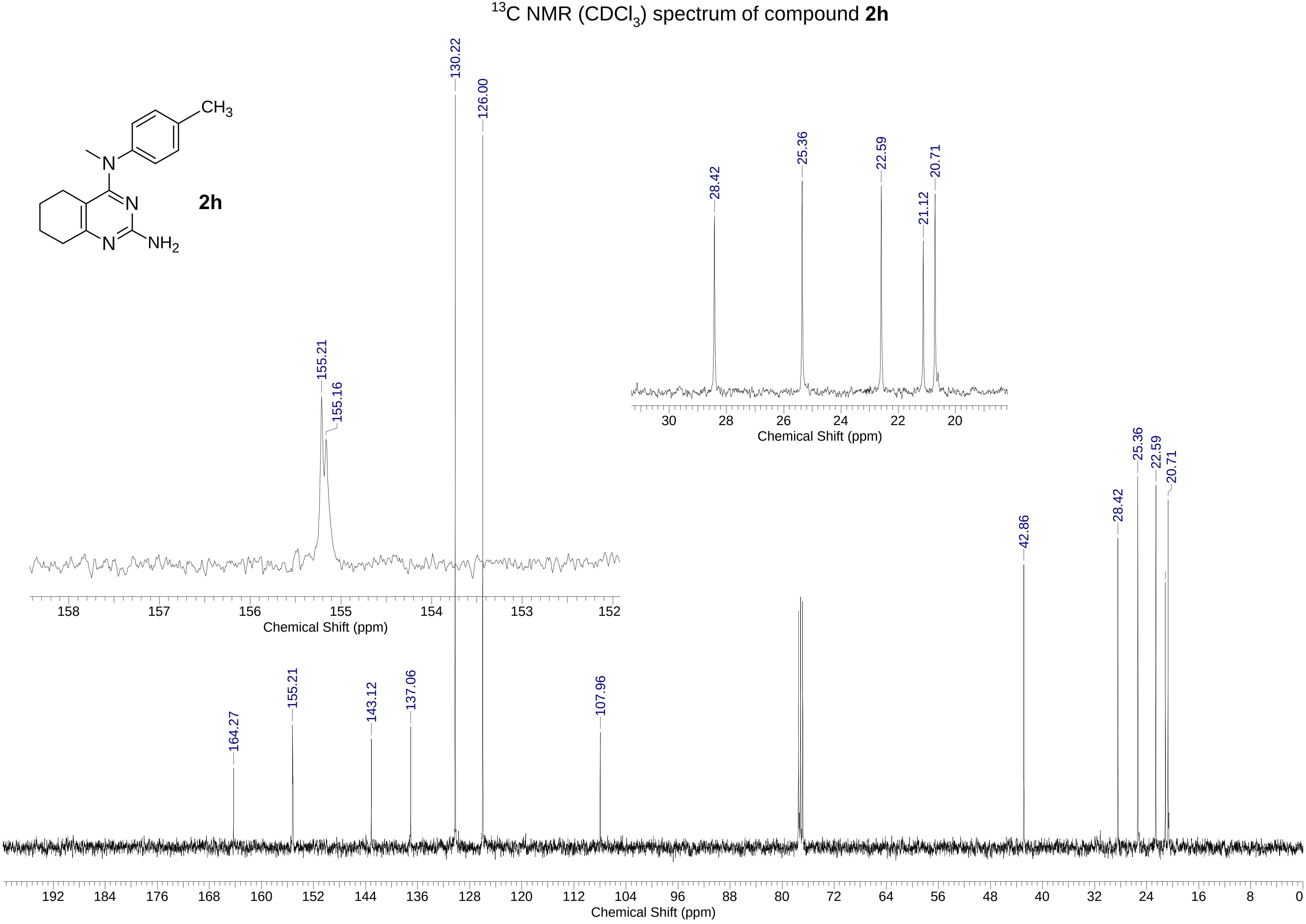

13C NMR (CDCl3) spectrum of compound 2h
CH3
N
N
N
NH2
2h
164.27
155.21
143.12
137.06
130.22
126.00
107.96
42.86
28.42
25.36
22.59
21.12
20.71
155.16
Chemical Shift (ppm)

HSQC NMR ($CDCl_3$) spectrum of compound **2h**

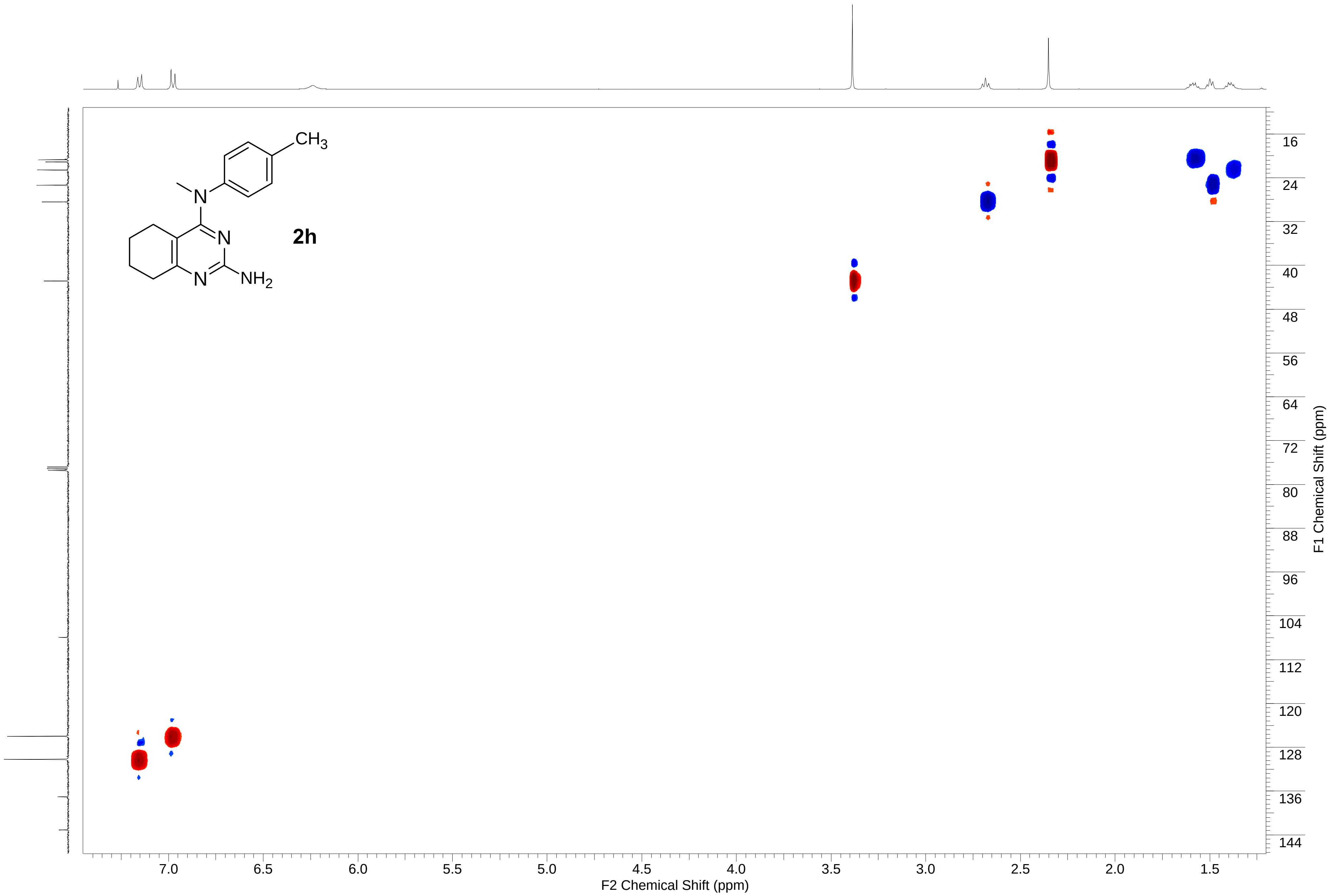

HMBC NMR ($CDCl_3$) spectrum of compound **2h**

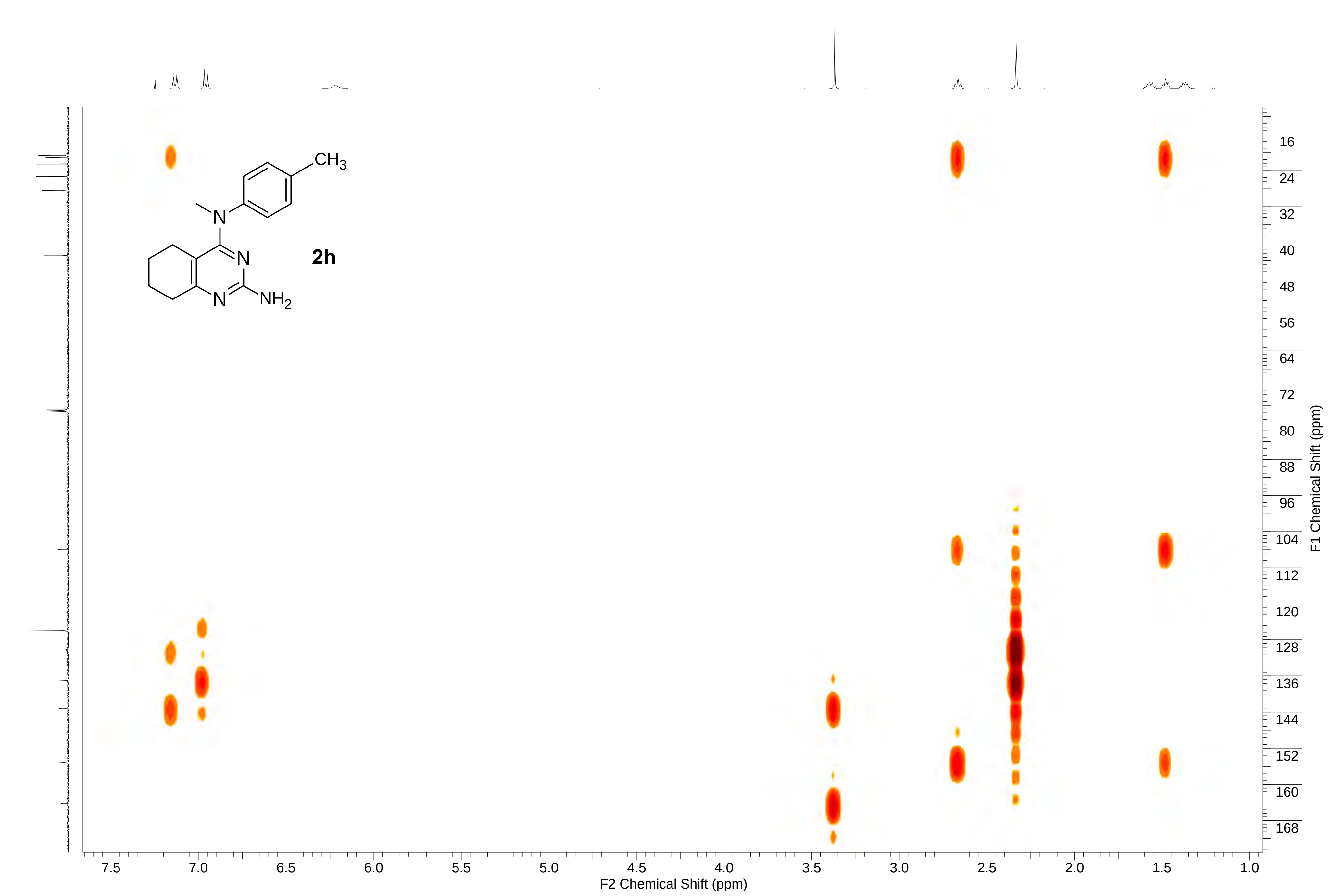

^{1}H NMR ($CDCl_3$) spectrum of compound **2i**

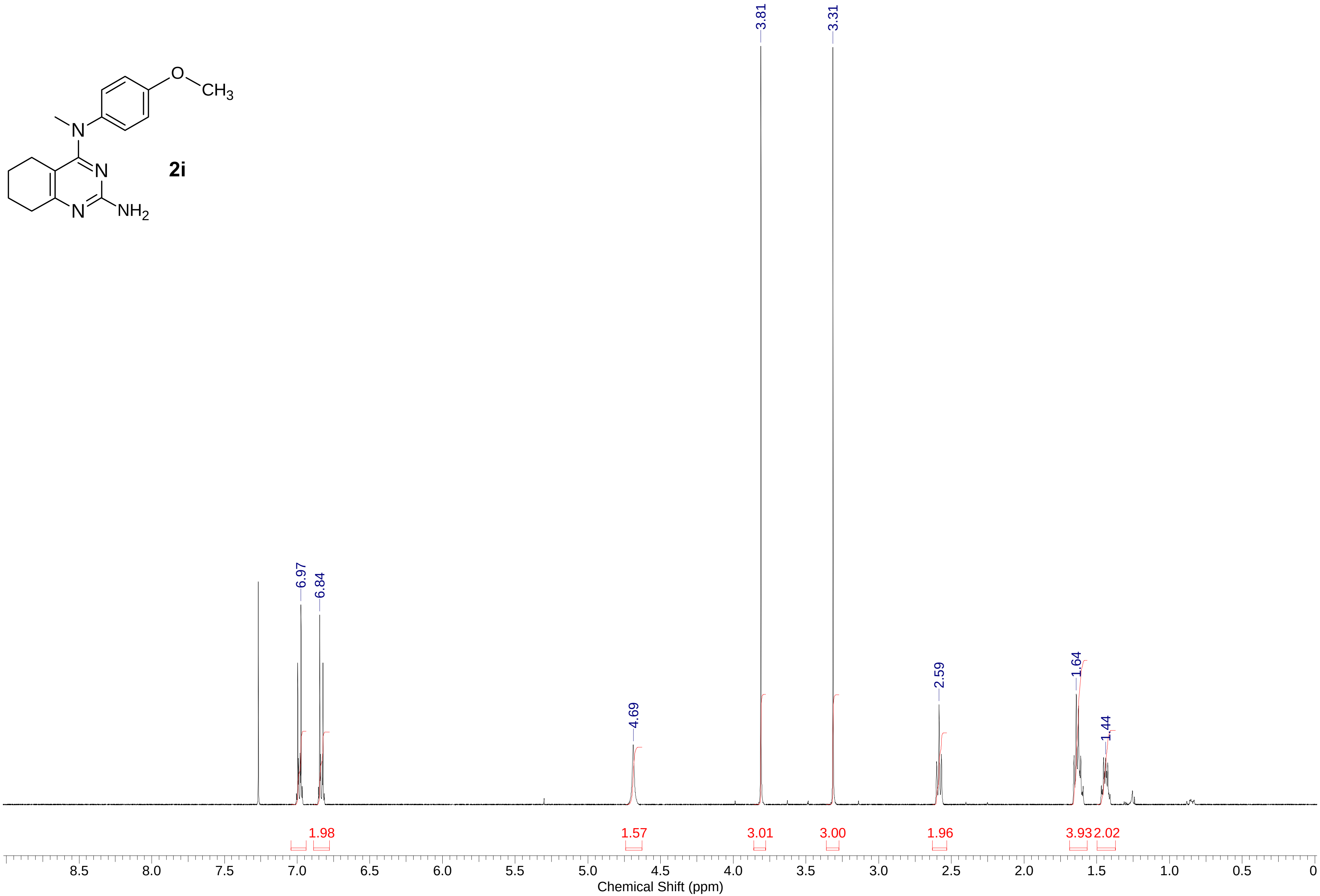

^{13}C NMR ($CDCl_3$) spectrum of compound **2i**

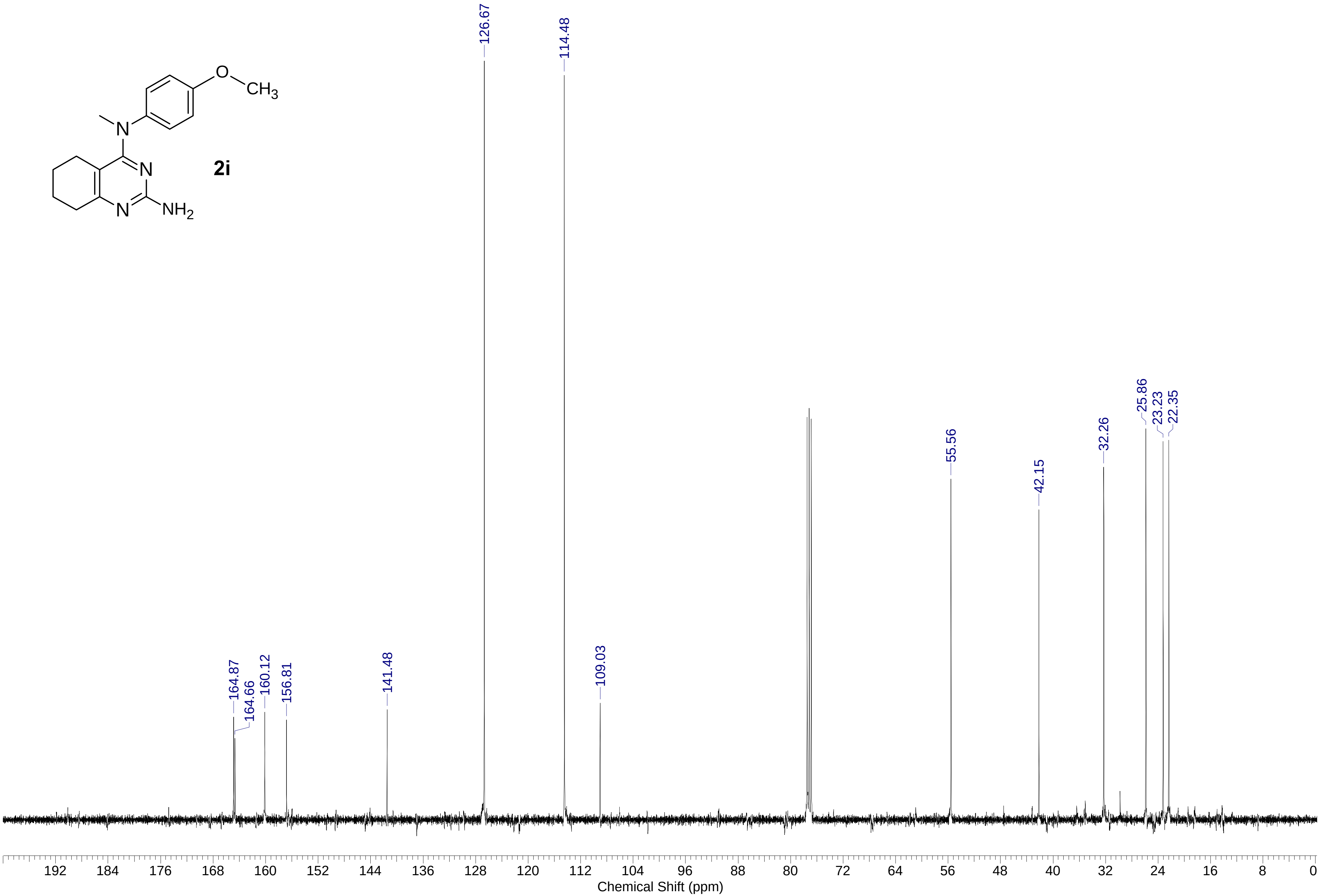

^1H NMR ($CDCl_3$) spectrum of compound **2j**

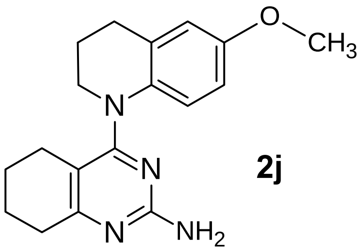

2j

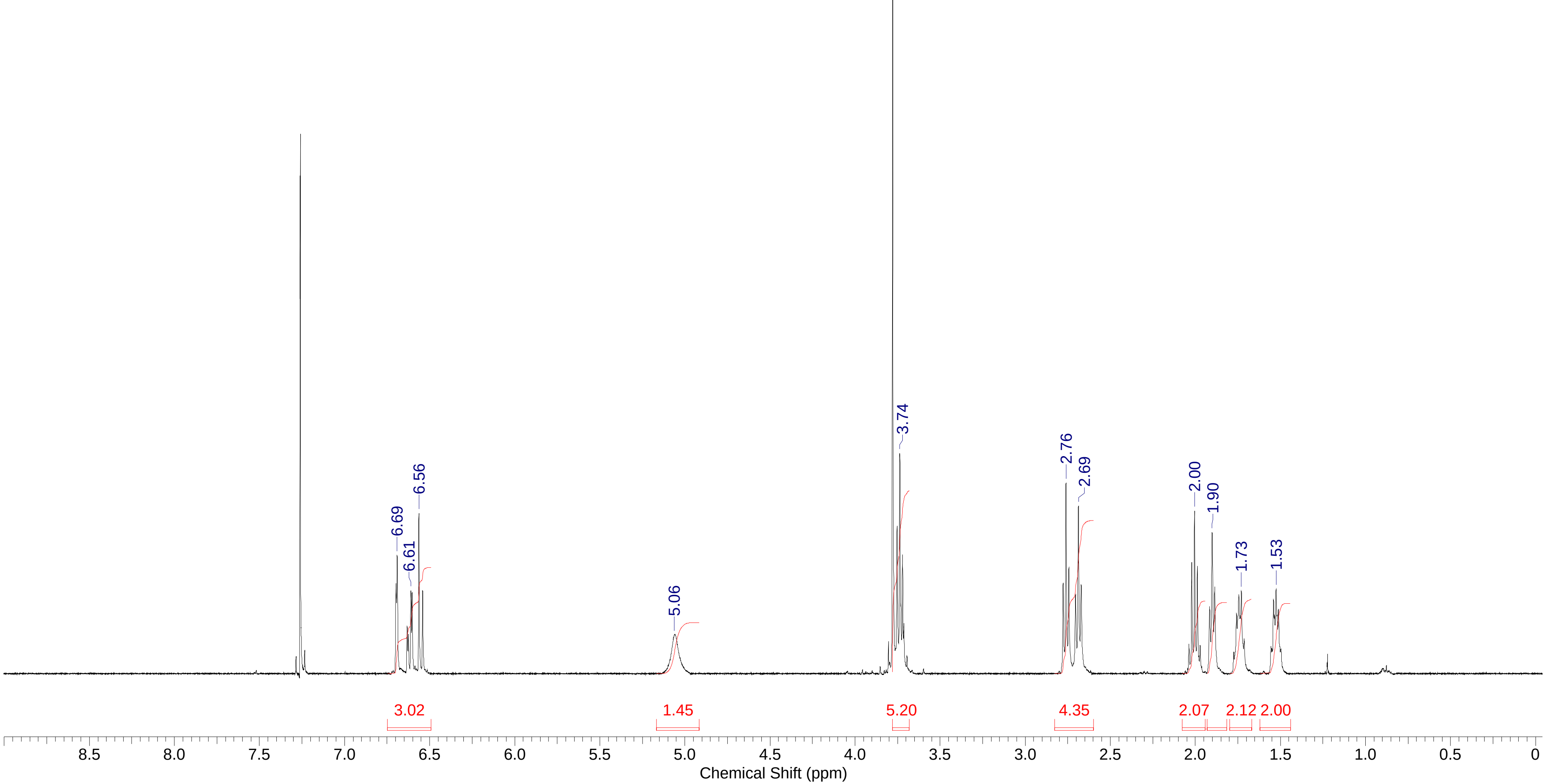

^{13}C NMR ($CDCl_3$) spectrum of compound **2j**

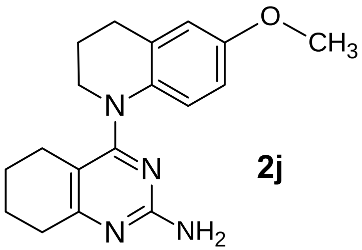

2j

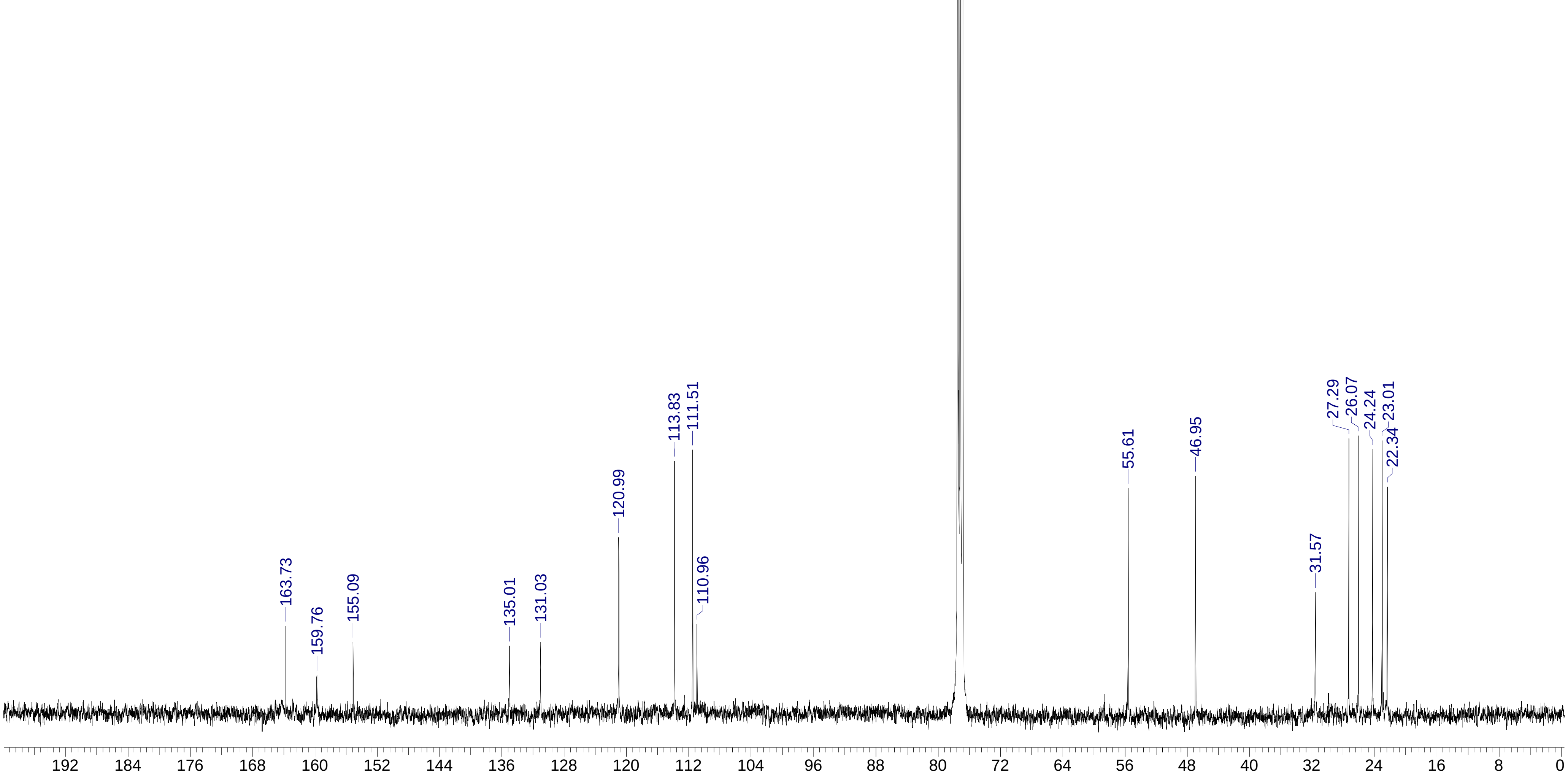

^1H NMR ($CDCl_3$) spectrum of compound **2k**

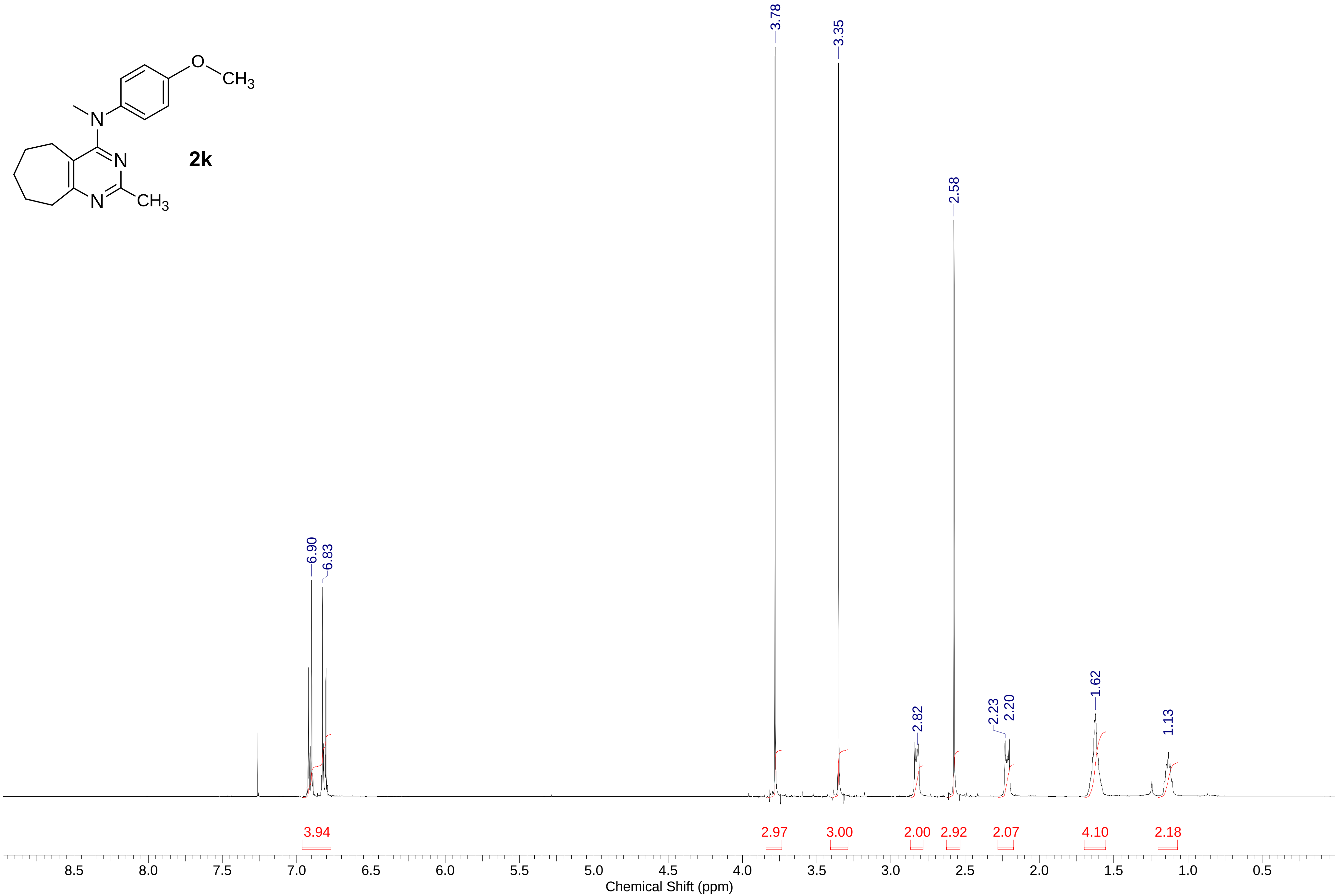

^{13}C NMR ($CDCl_3$) spectrum of compound **2k**

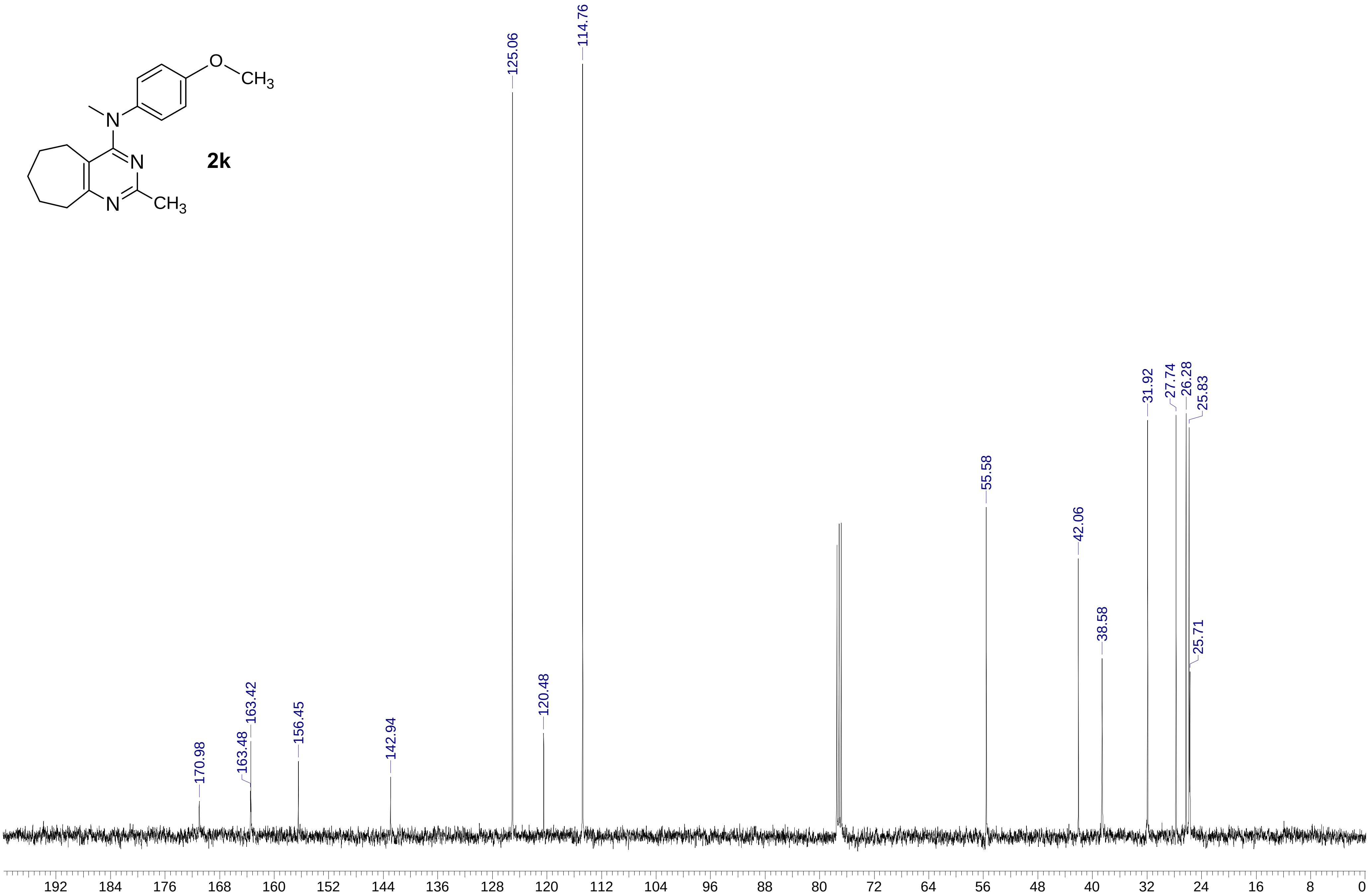

HSQC NMR ($CDCl_3$) spectrum of compound **2k**

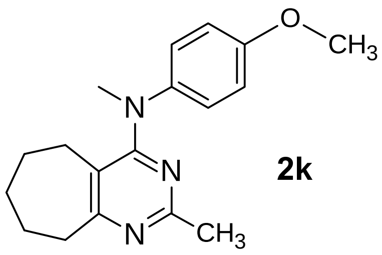

2k

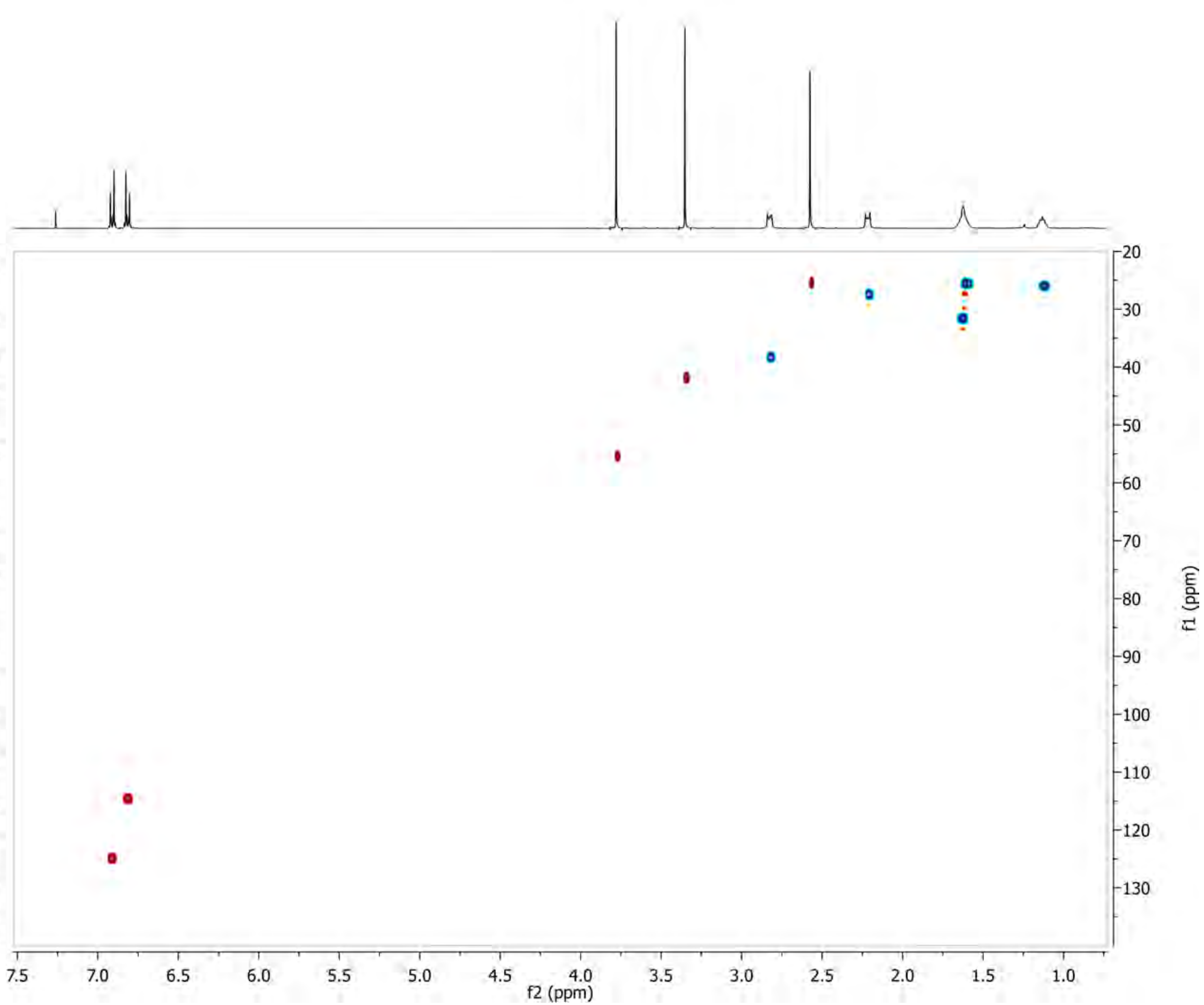

HMBC NMR ($CDCl_3$) spectrum of compound **2k**

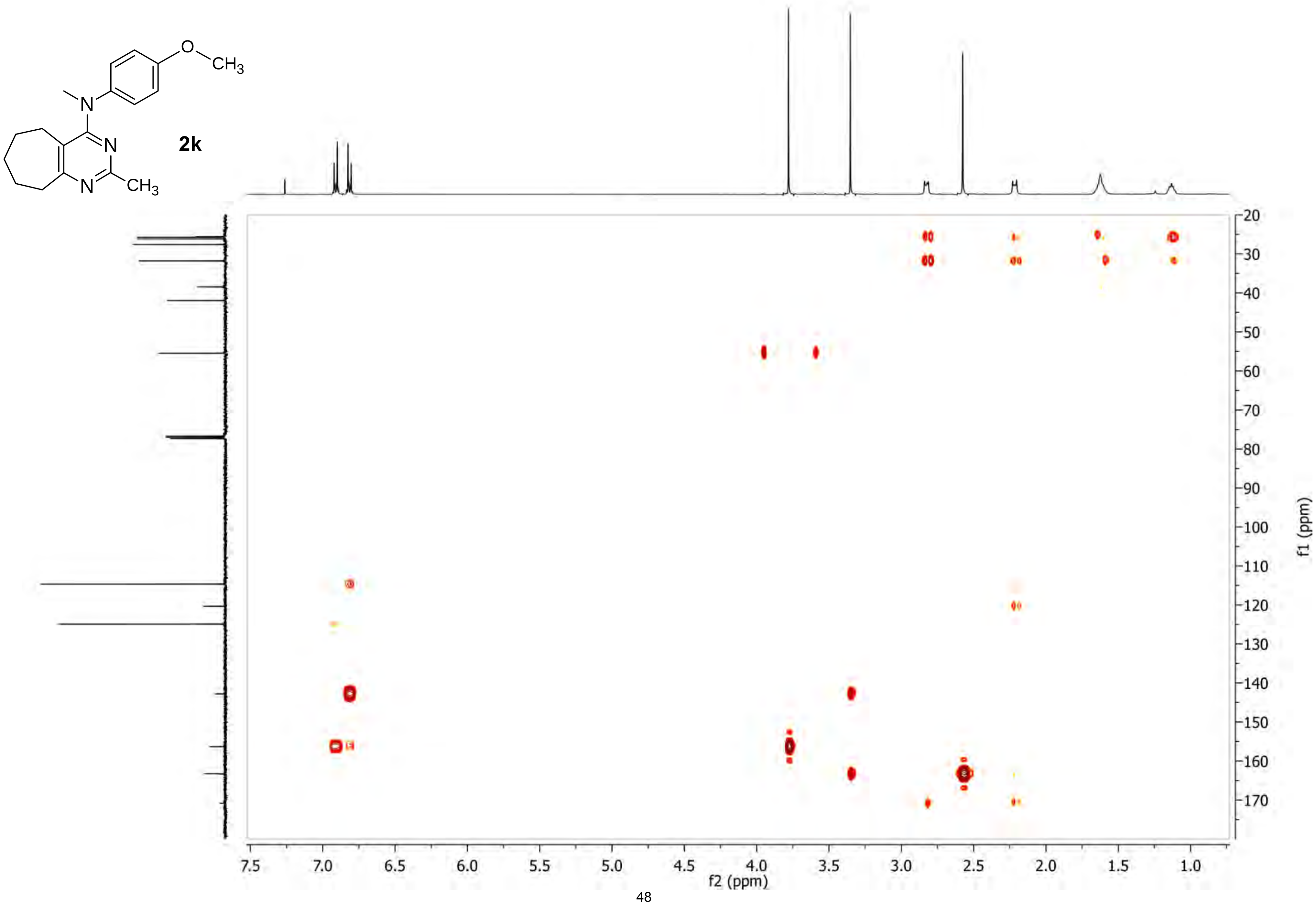

^{1}H NMR ($CDCl_3$) spectrum of compound **2l**

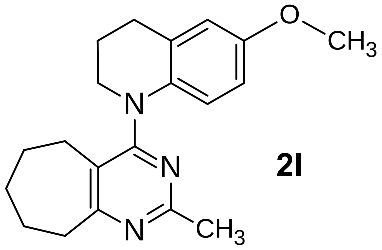

2l

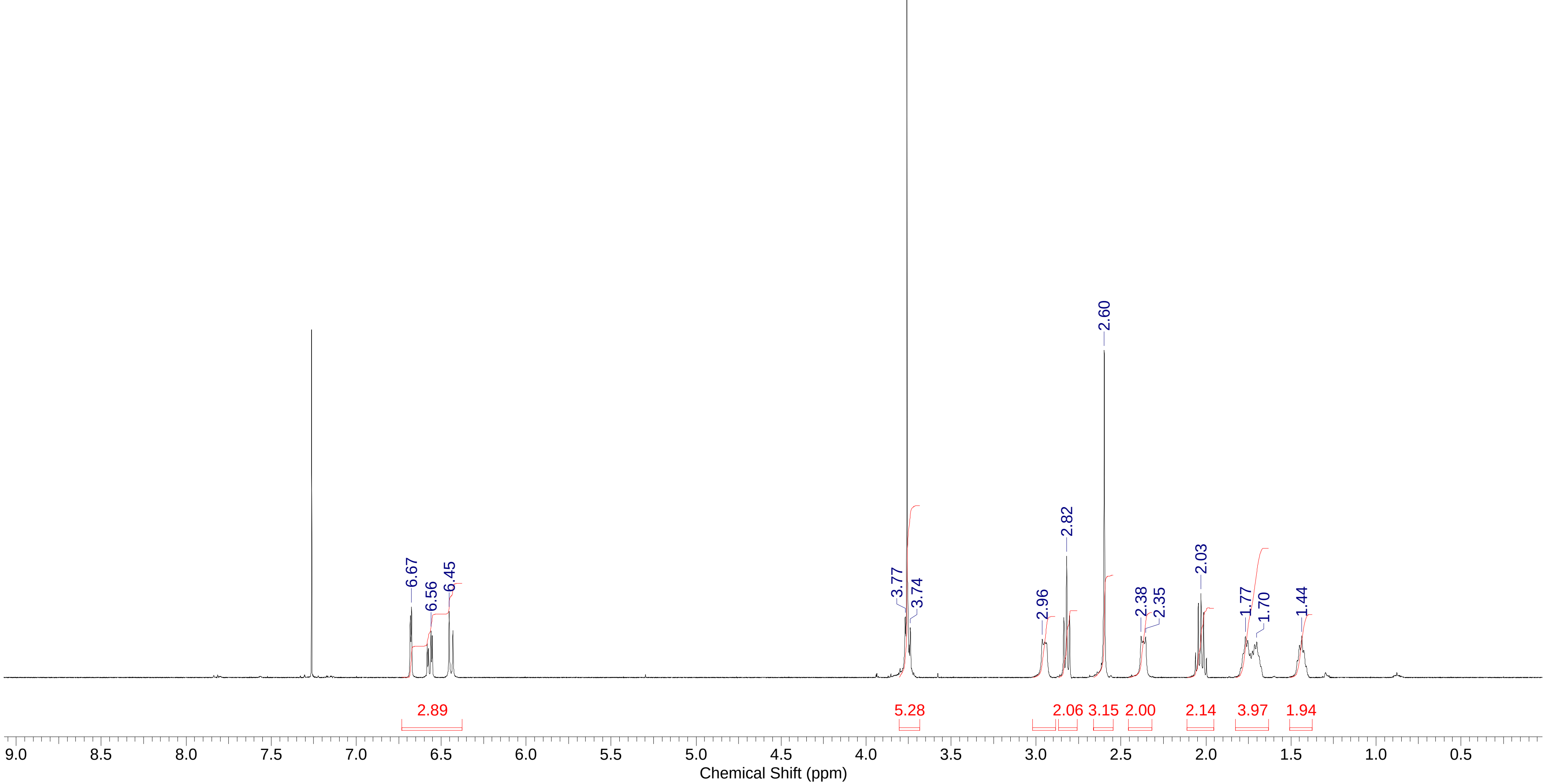

^{13}C NMR ($CDCl_3$) spectrum of compound **2l**

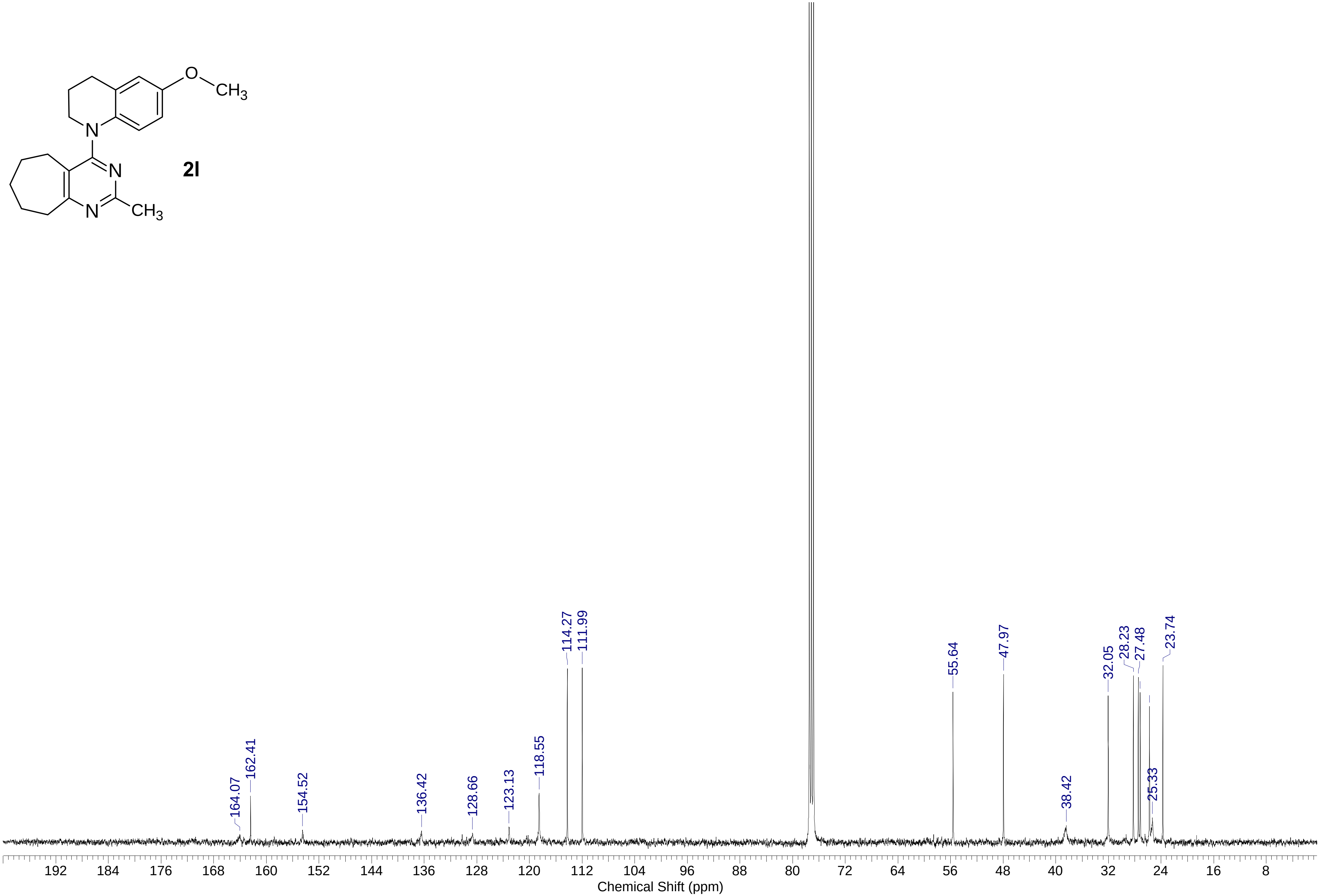

2l

^{1}H NMR ($CDCl_3$) spectrum of compound **2m**

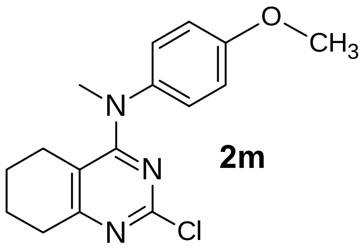

2m

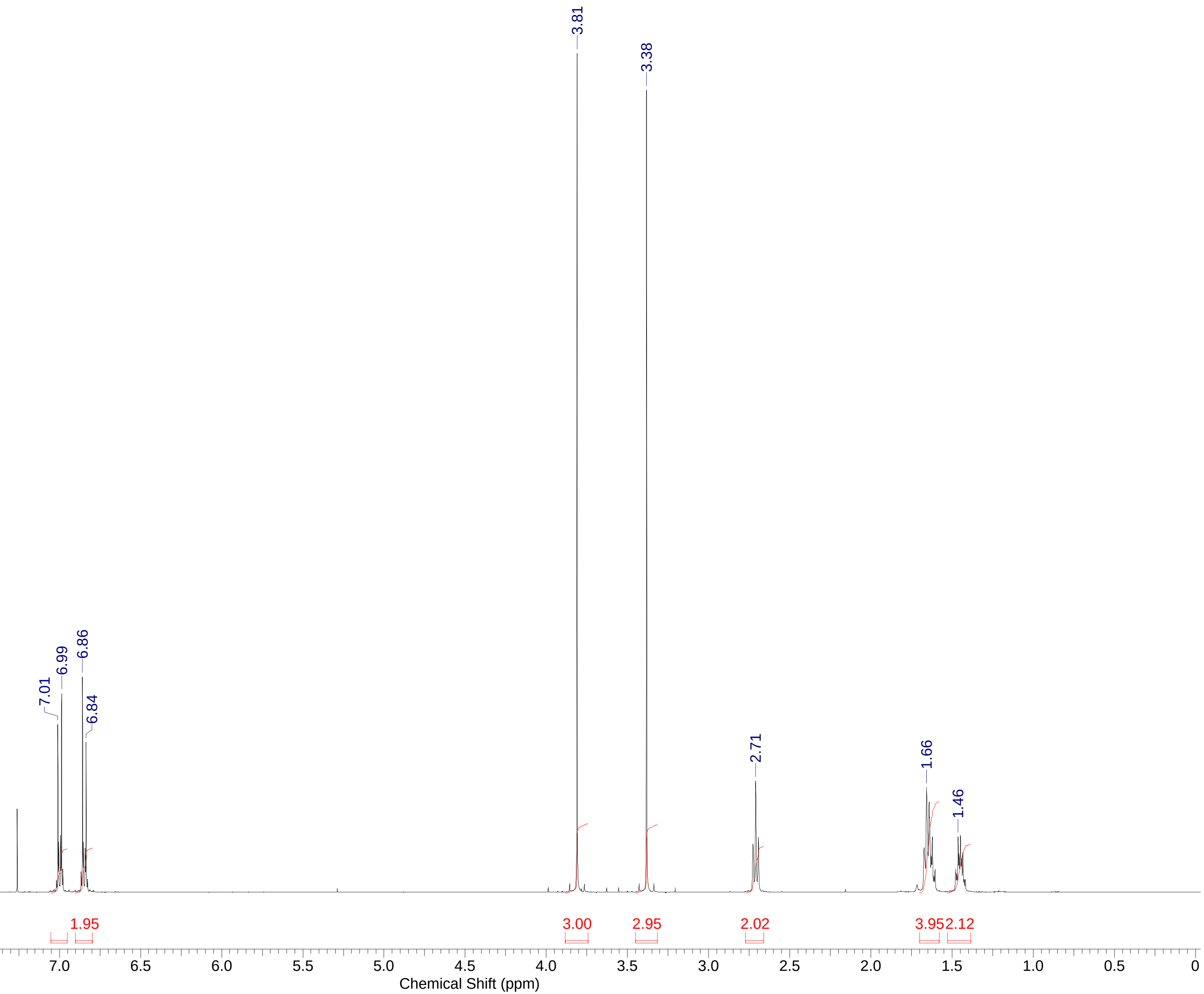

^{13}C NMR ($CDCl_3$) spectrum of compound **2m**

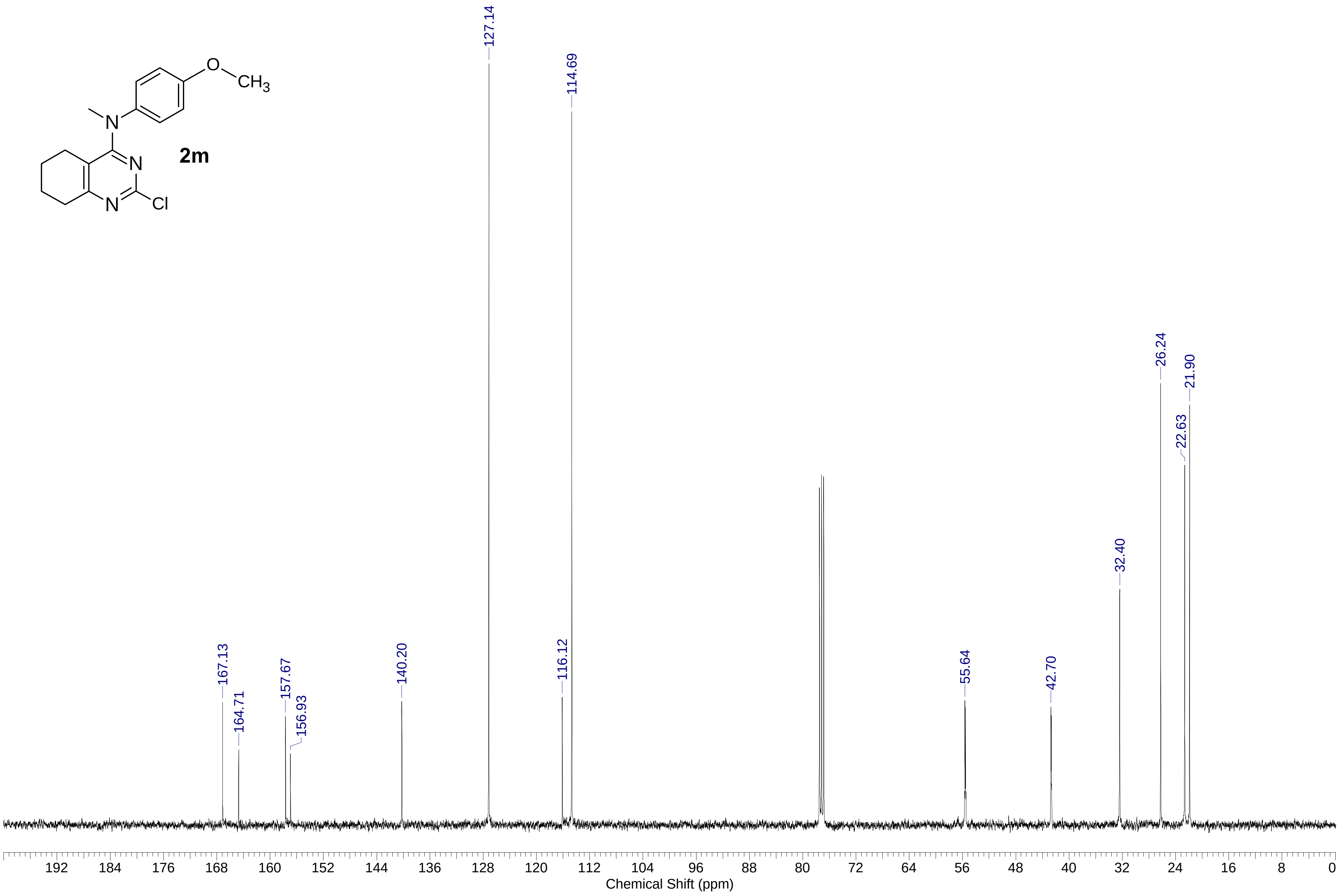

HSQC NMR ($CDCl_3$) spectrum of compound **2m**

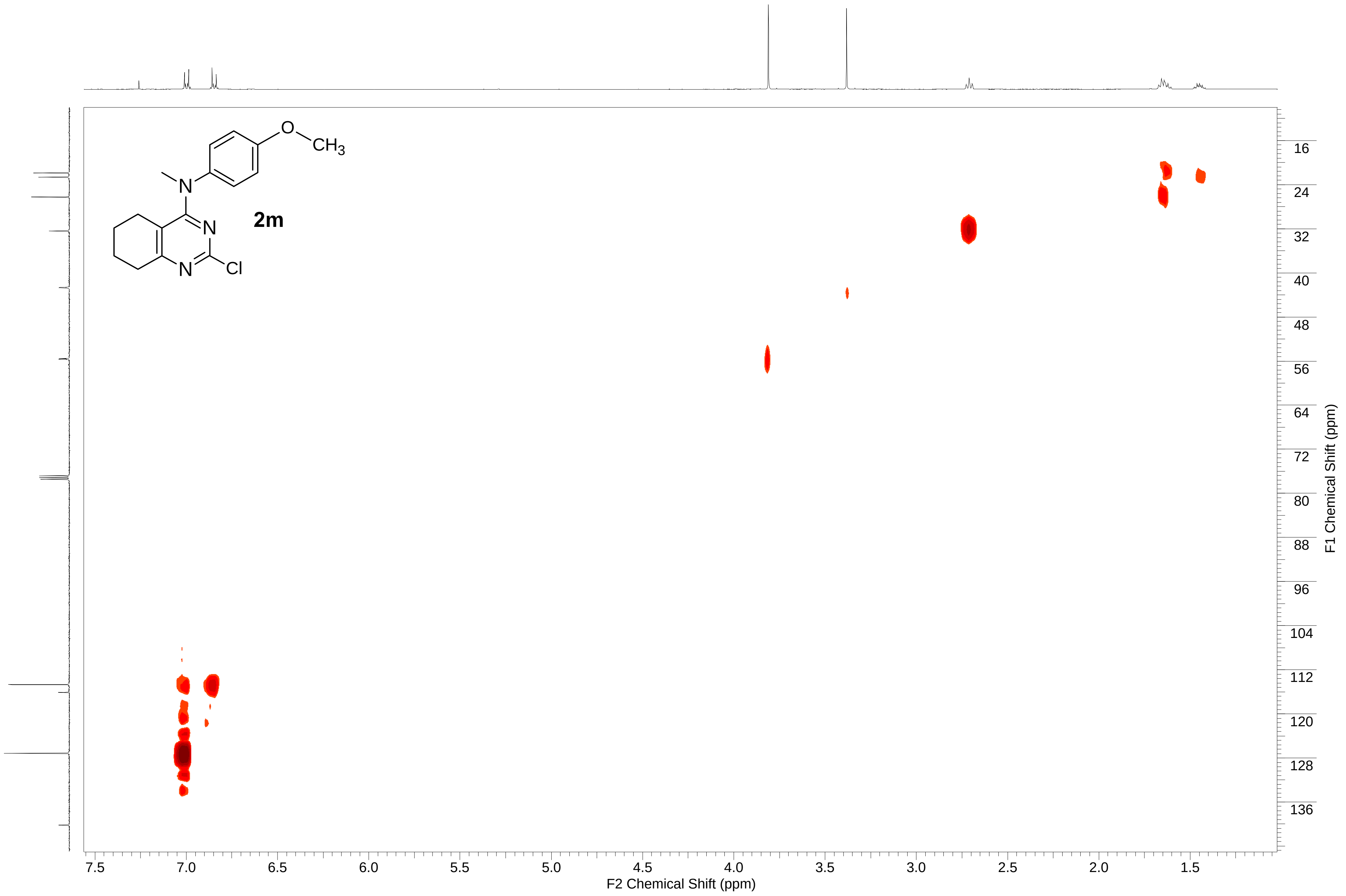

HMBC NMR ($CDCl_3$) spectrum of compound **2m**

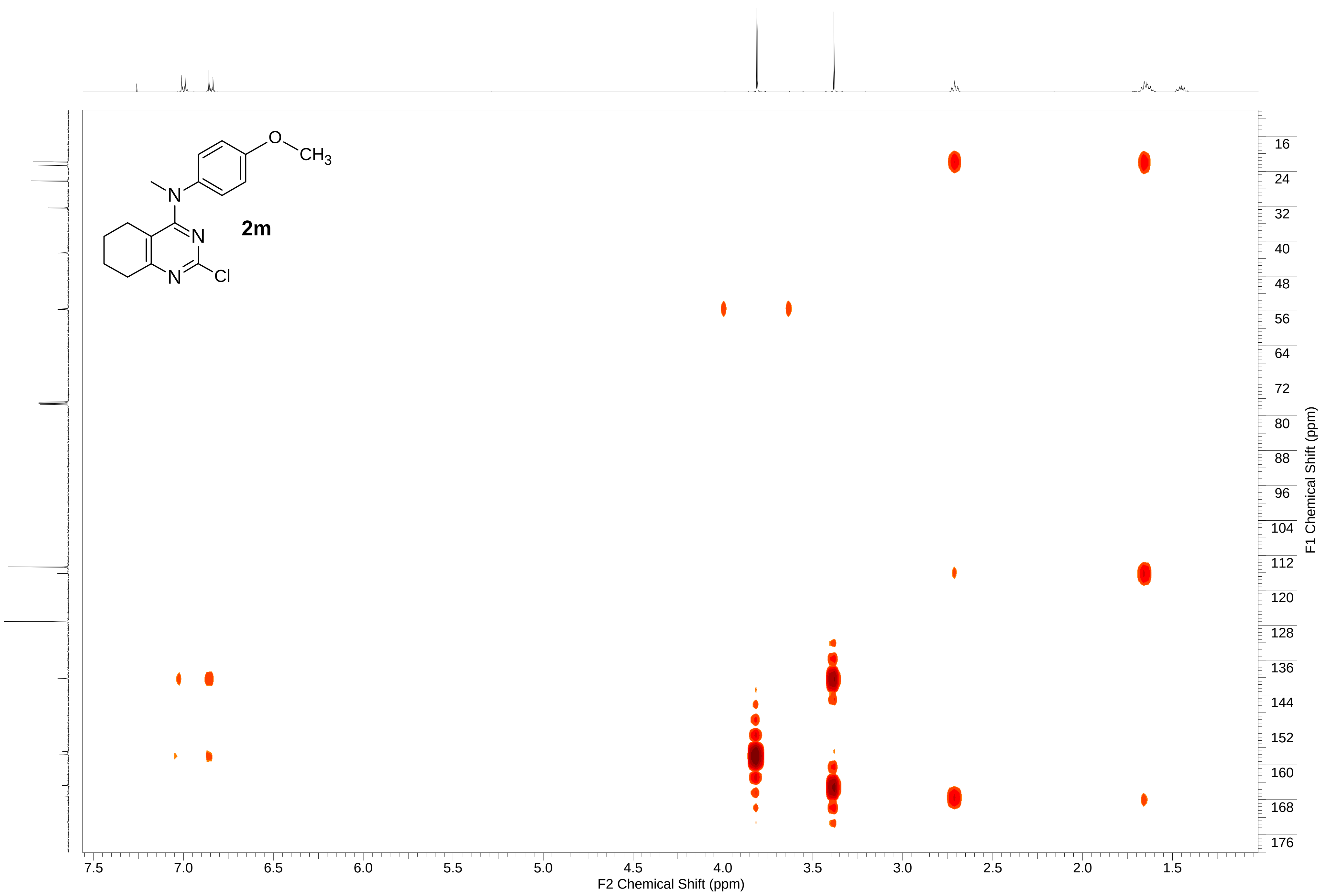

^{1}H NMR ($CDCl_3$) spectrum of compound **2n**

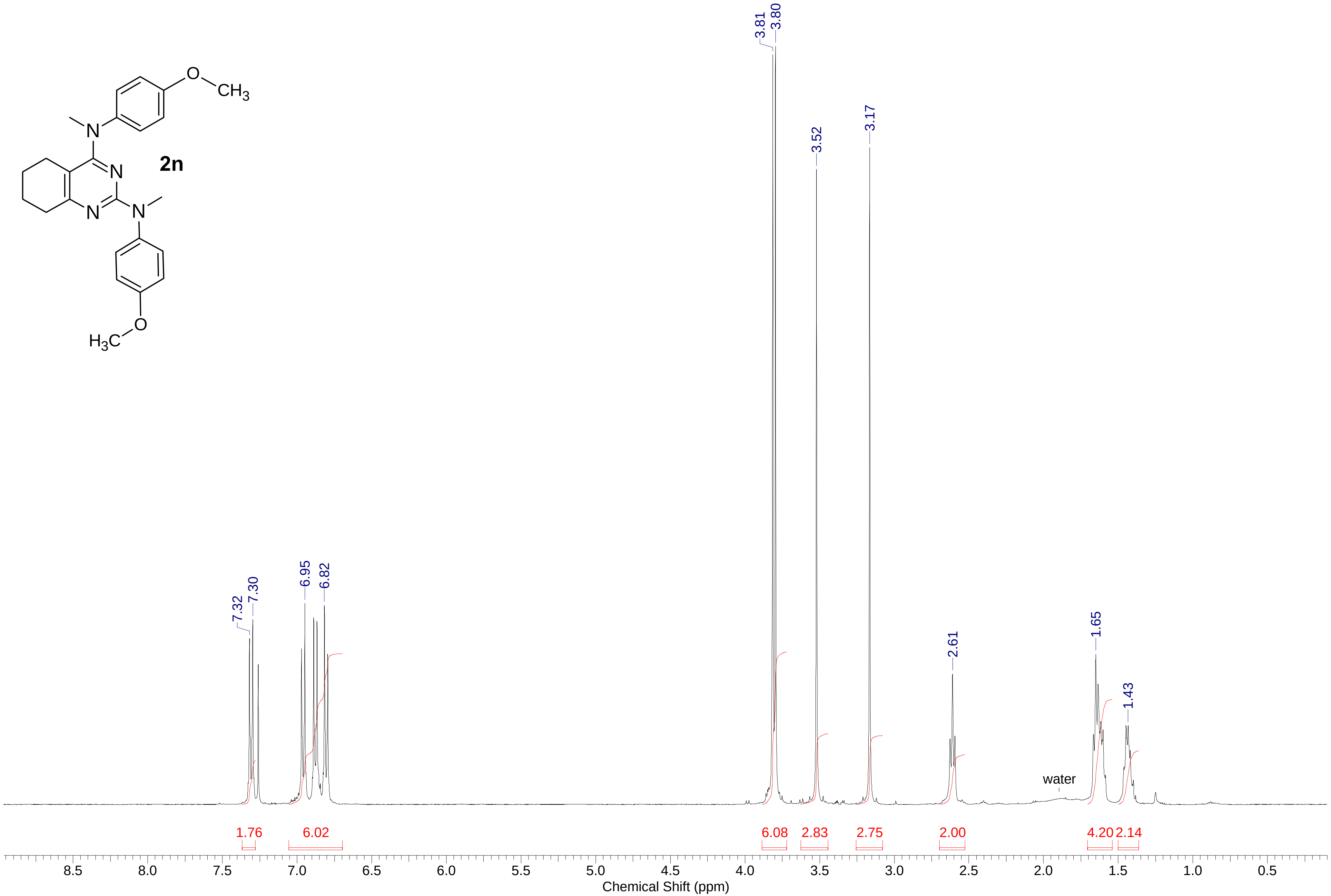

^{13}C NMR ($CDCl_3$) spectrum of compound **2n**

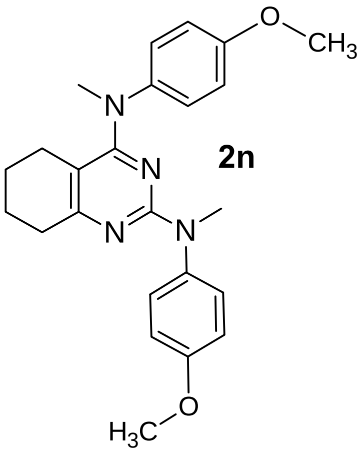

2n

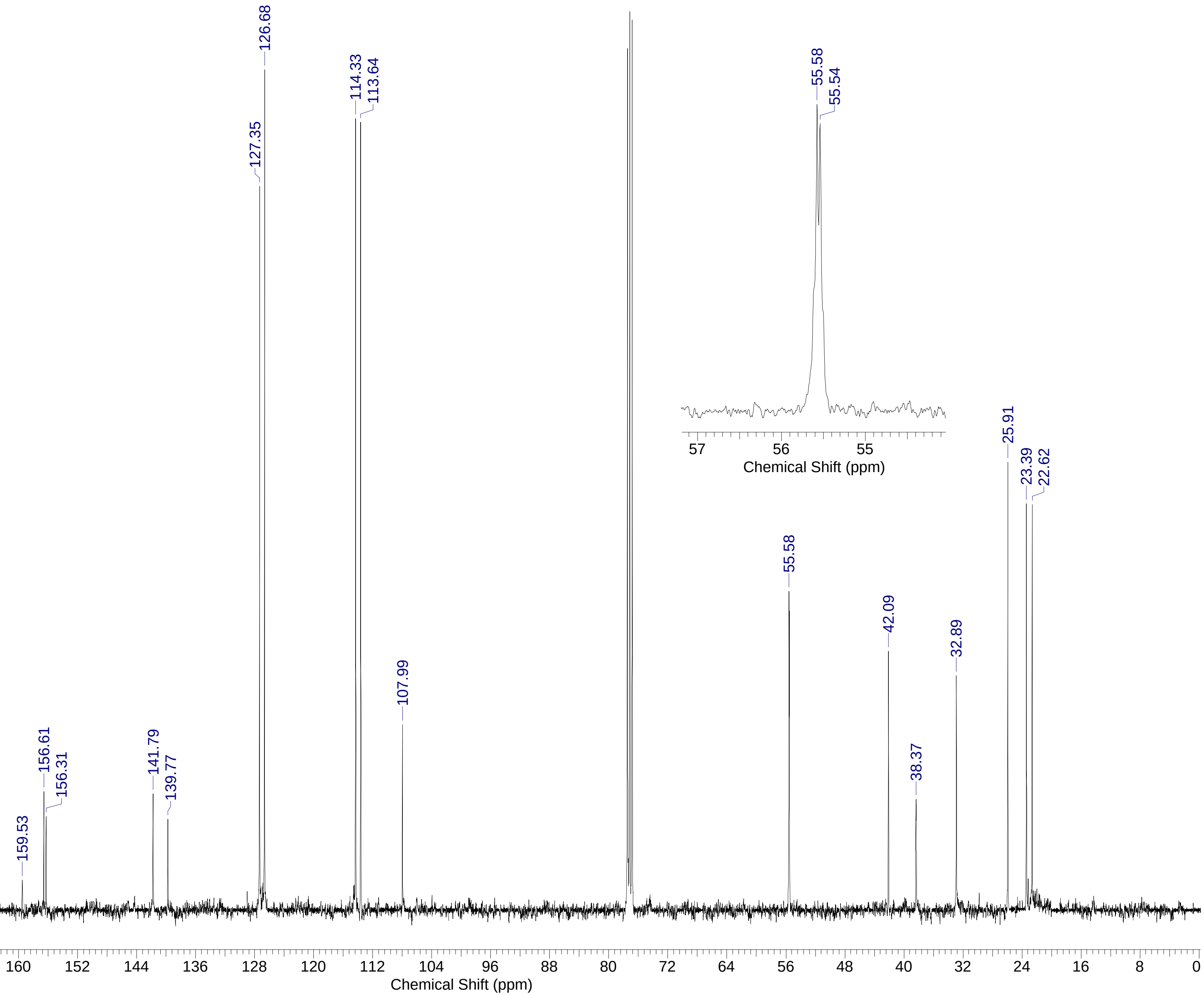

HSQC NMR ($CDCl_3$) spectrum of compound **2n**

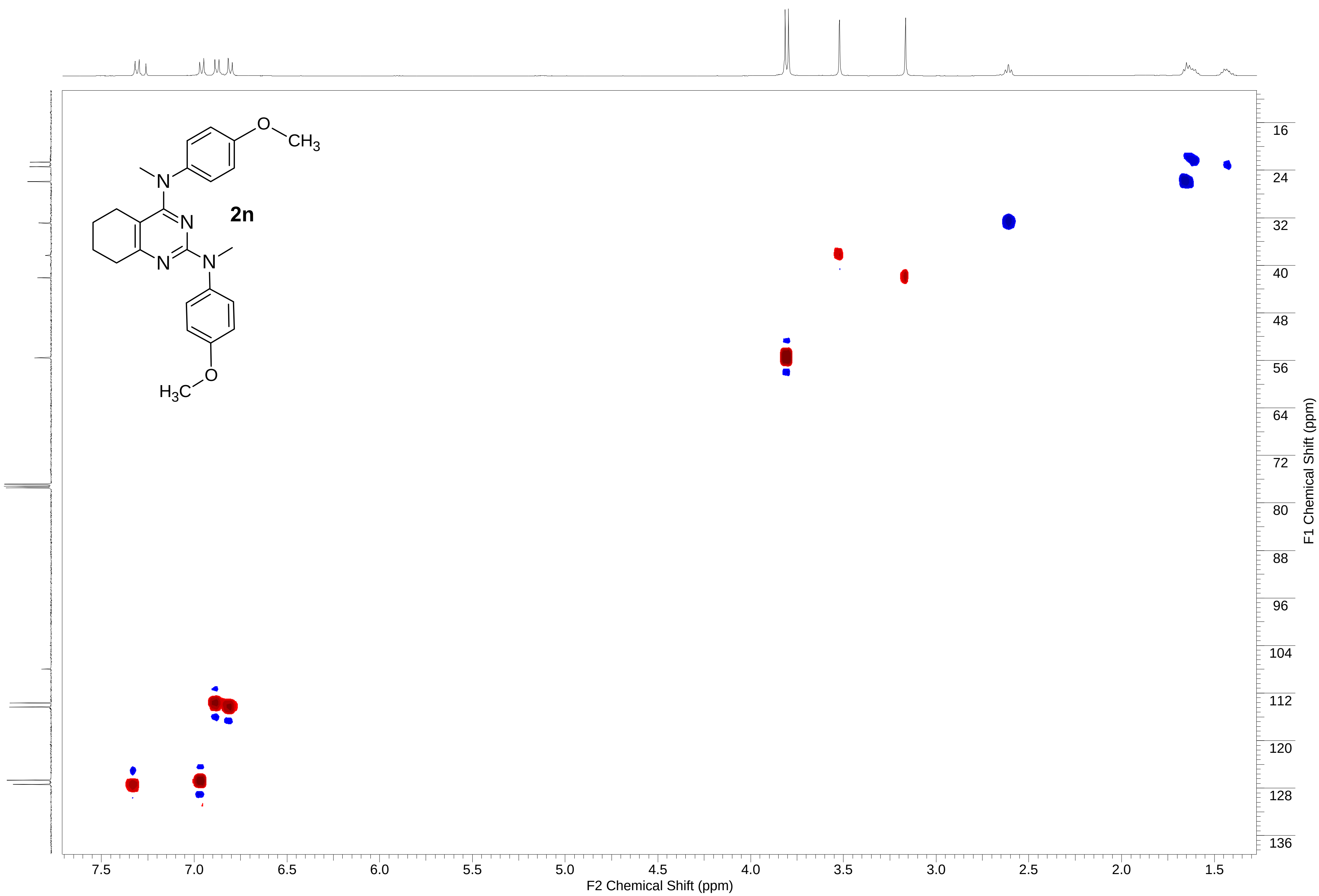

HMBC NMR ($CDCl_3$) spectrum of compound **2n**

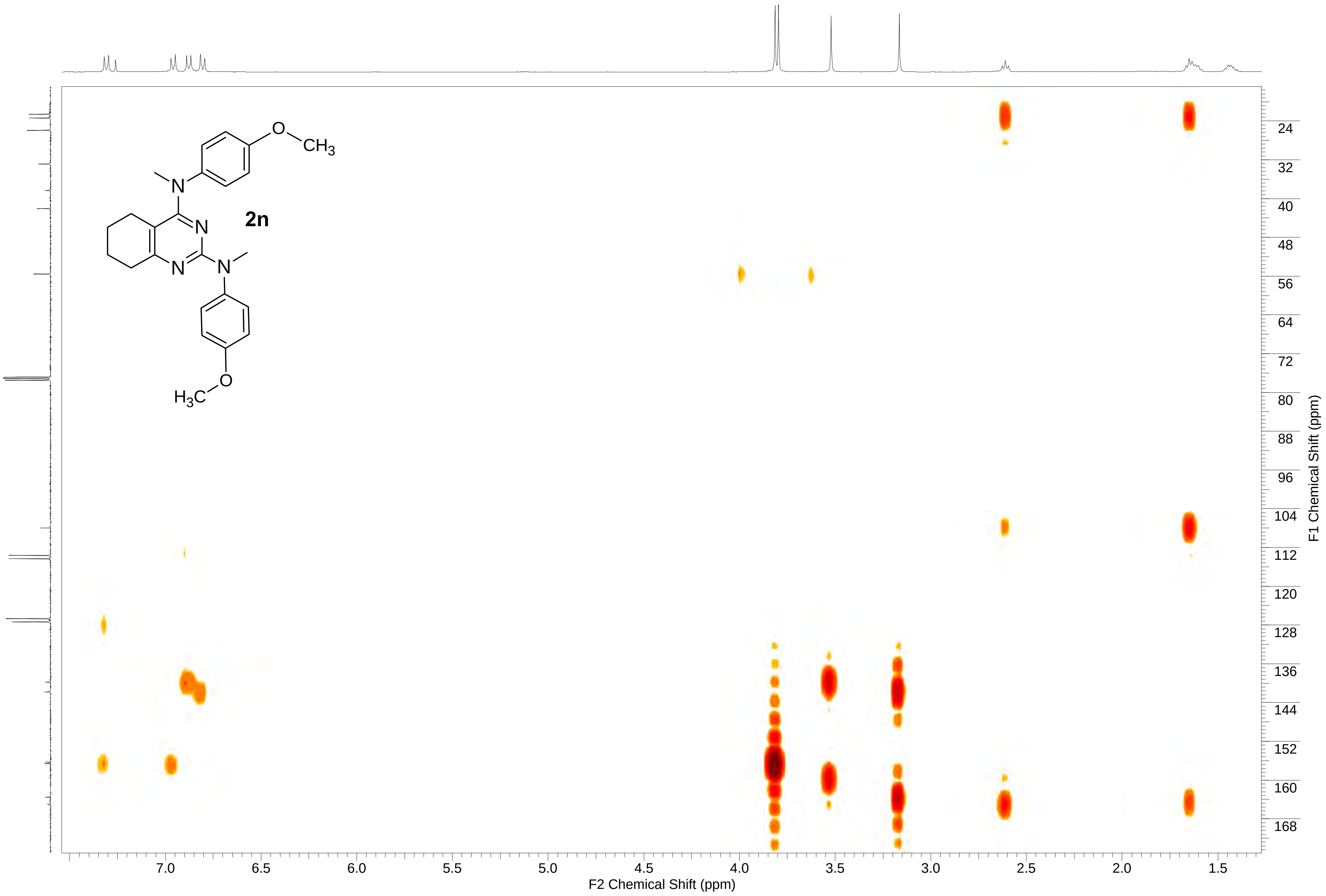

^1H NMR ($CDCl_3$) spectrum of compound **2o**

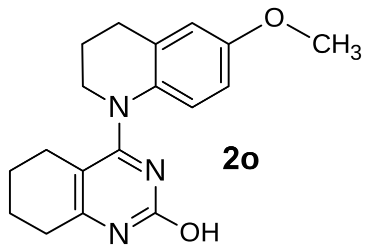

2o

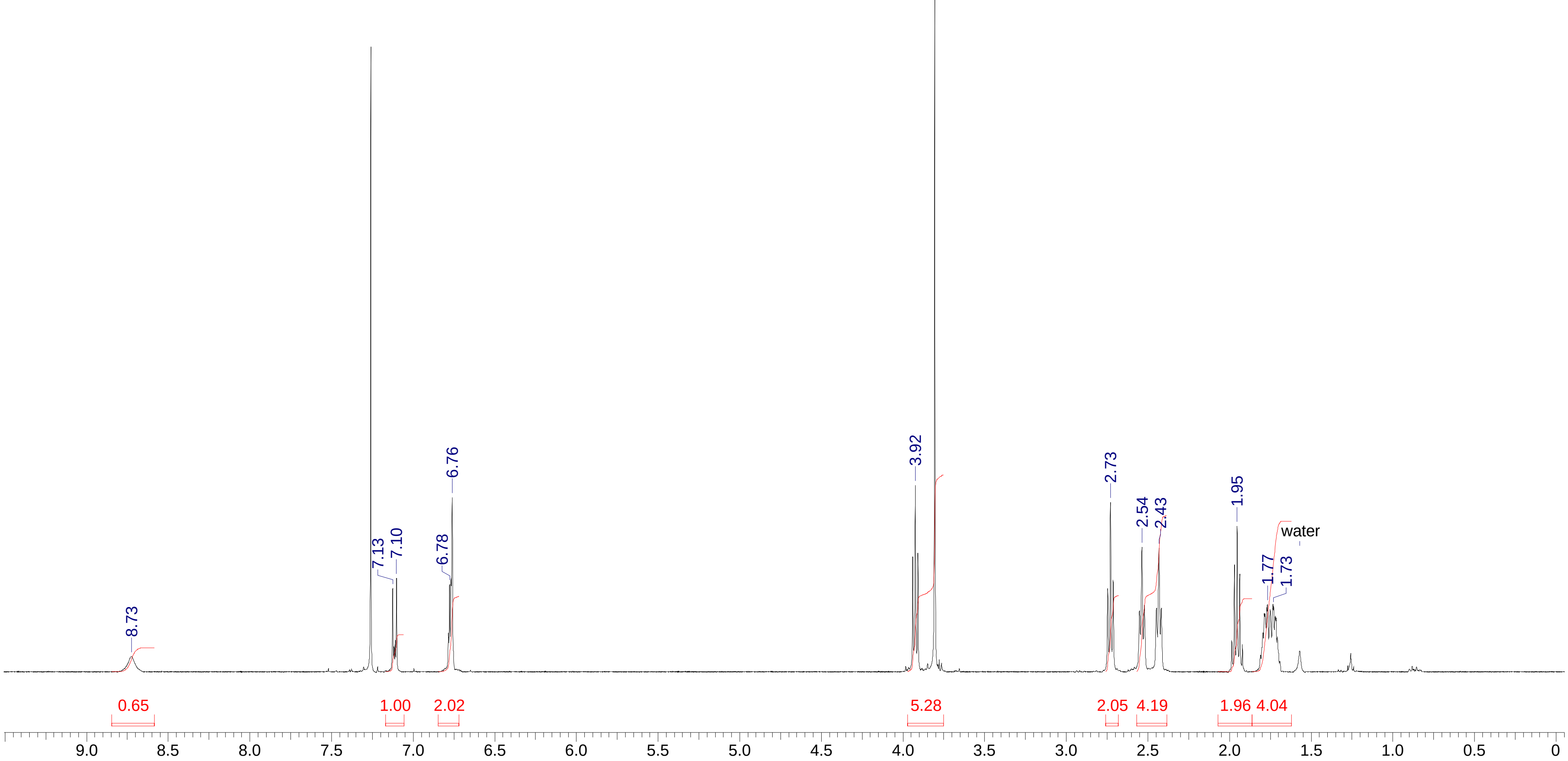

^{13}C NMR ($CDCl_3$) spectrum of compound **2o**

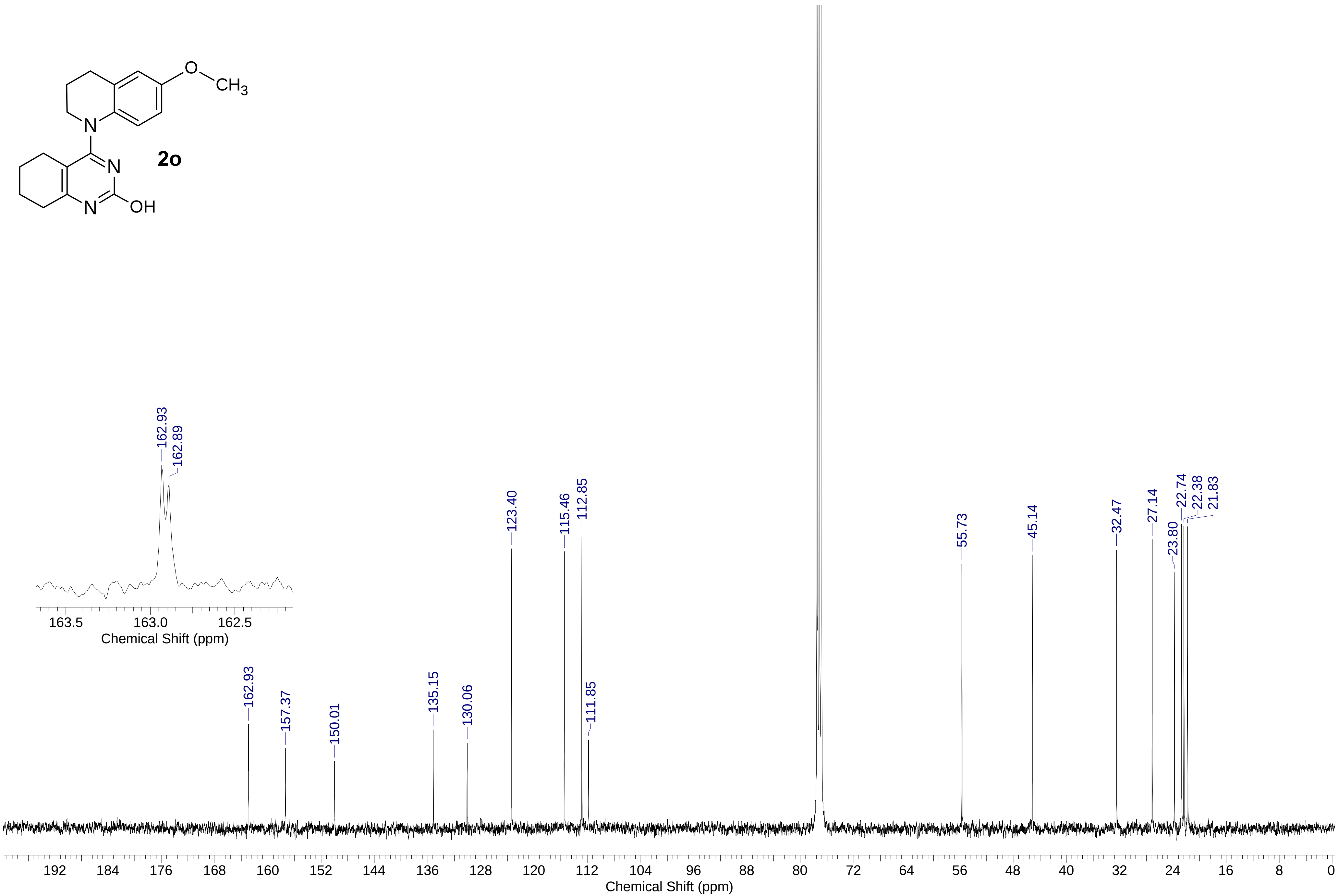

HMBC NMR ($CDCl_3$) spectrum of compound **2o**

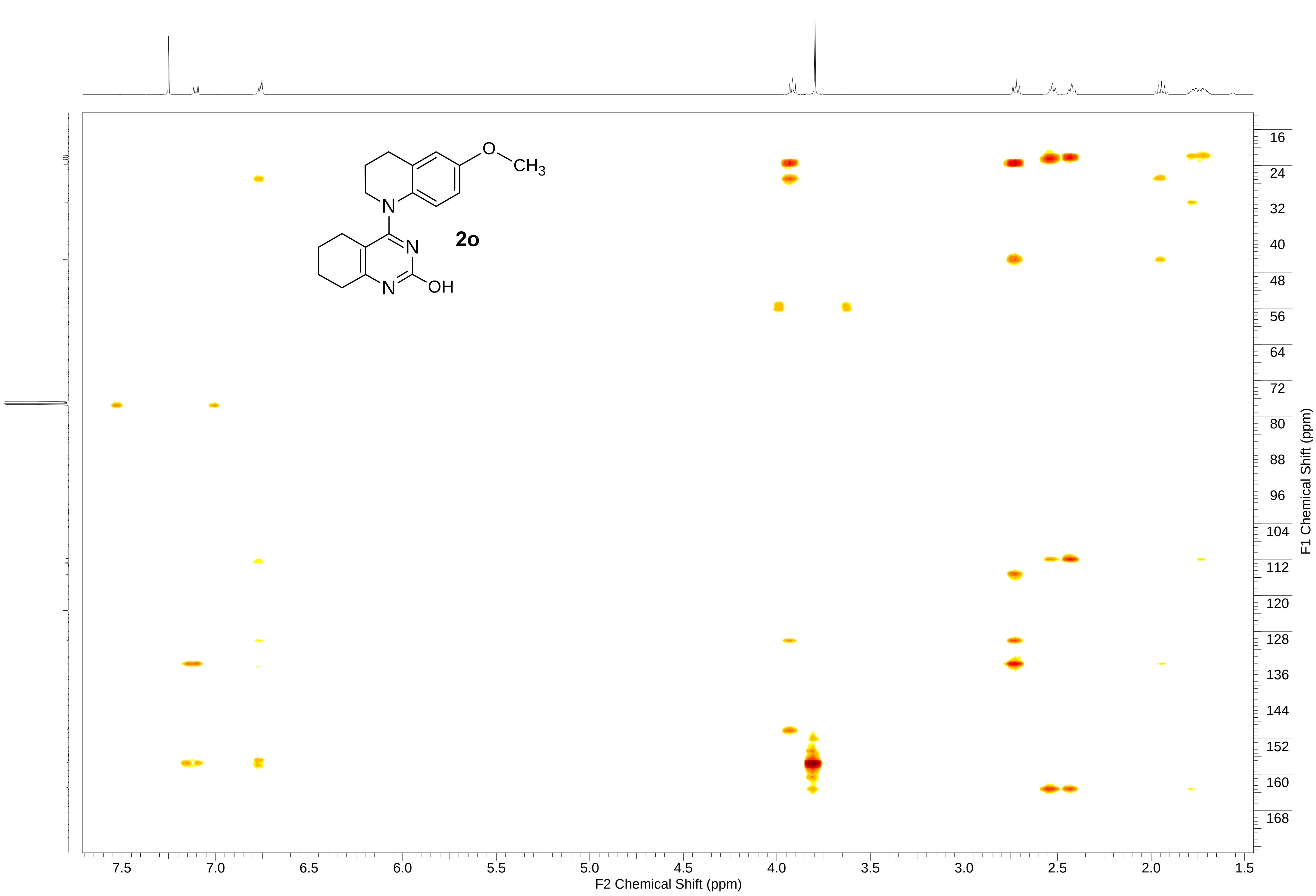

^{1}H NMR ($CDCl_3$) spectrum of compound **2p**

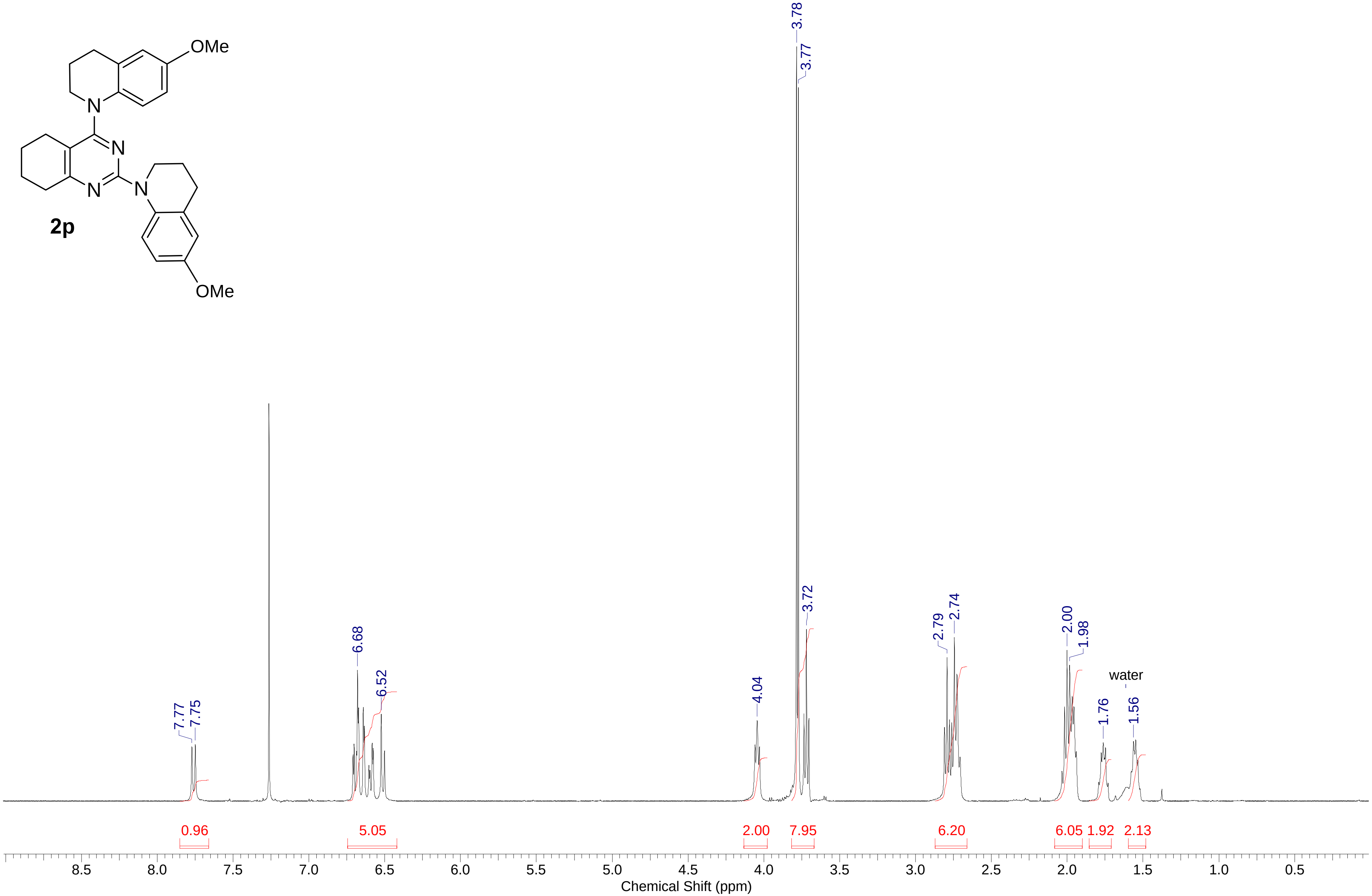

^{13}C NMR ($CDCl_3$) spectrum of compound **2p**

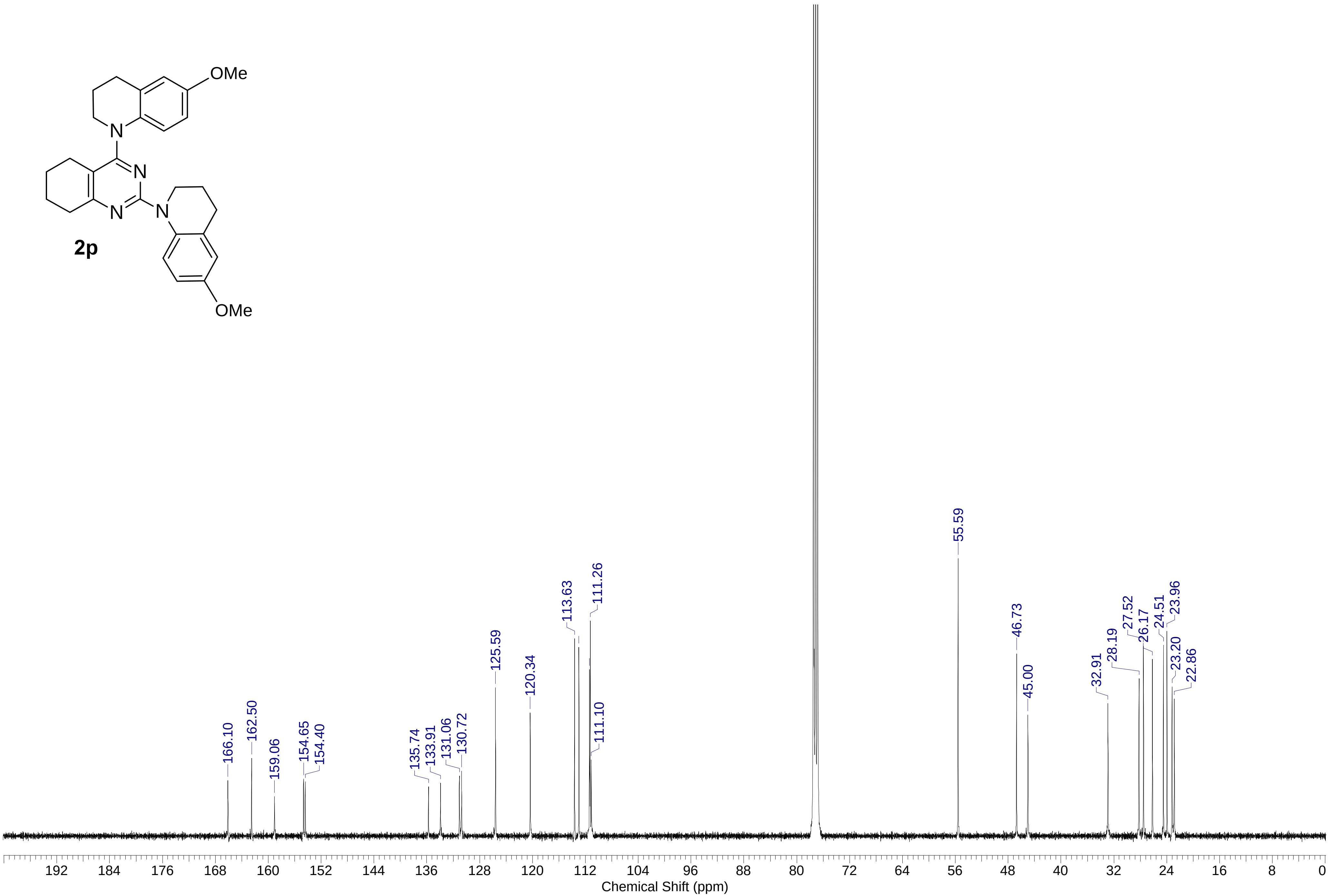

HSQC NMR ($CDCl_3$) spectrum of compound **2p**

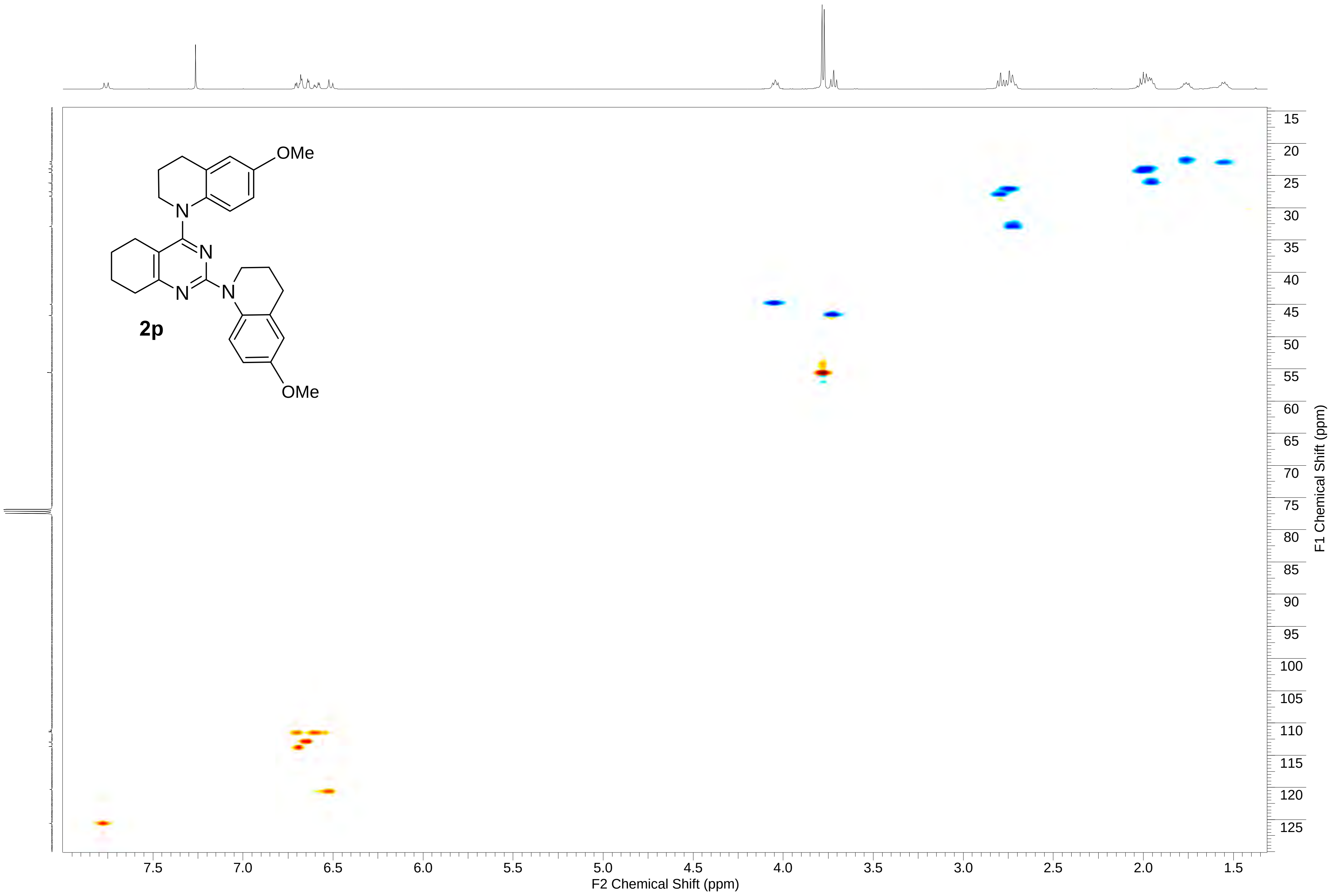

HMBC NMR ($CDCl_3$) spectrum (low field) of compound **2p**

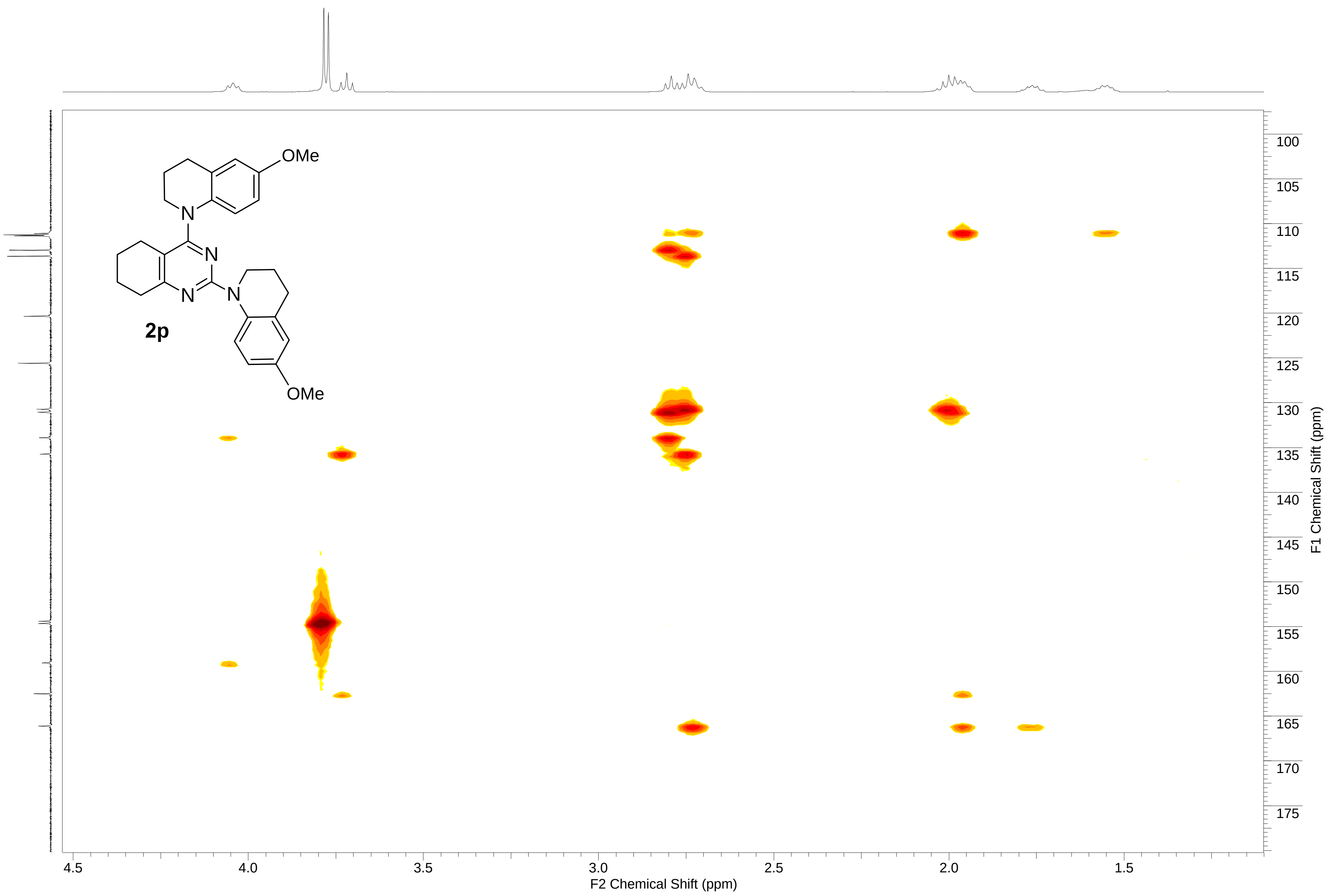

^{1}H NMR ($CDCl_3$) spectrum of compound **2q**

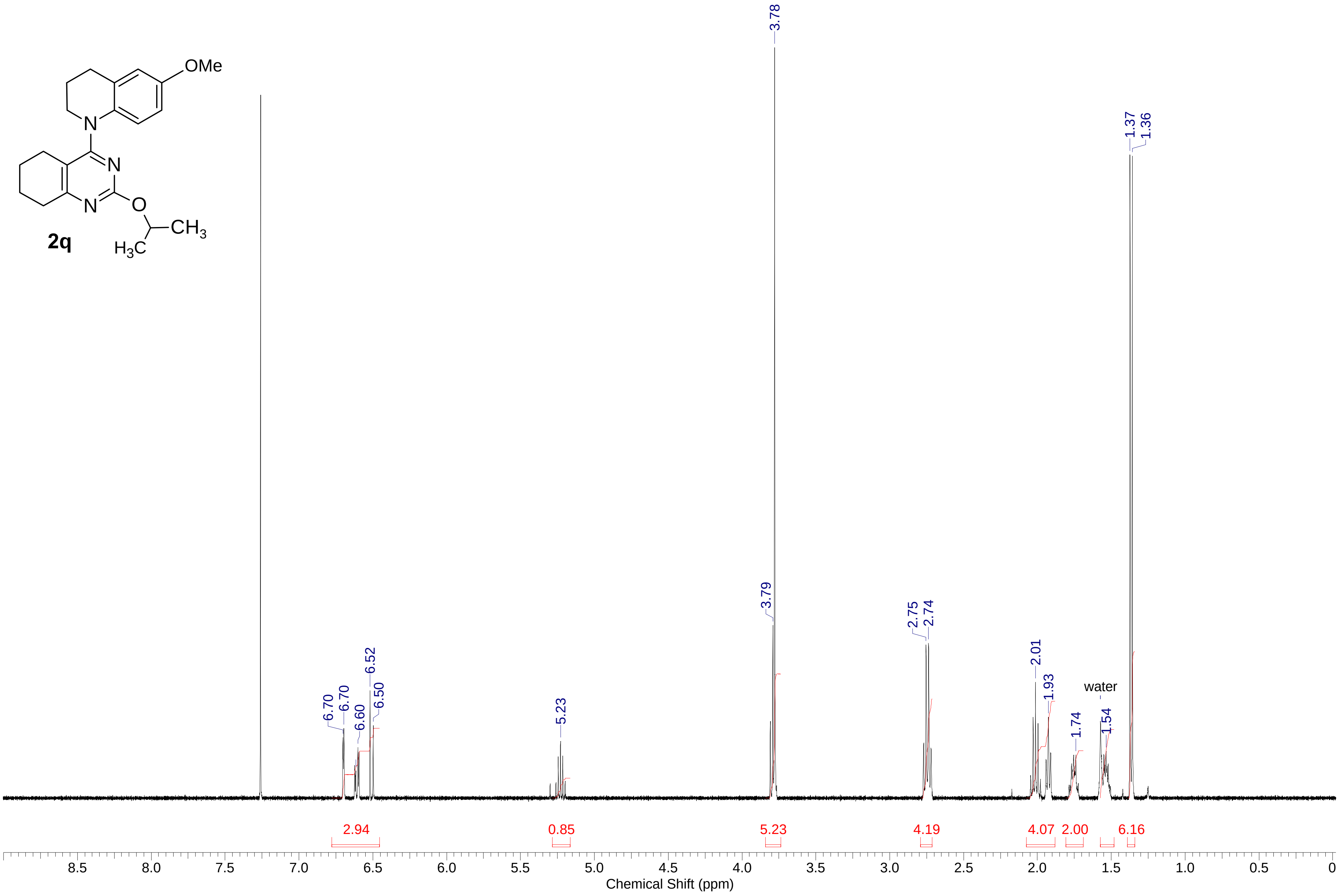

^{13}C NMR ($CDCl_3$) spectrum of compound **2q**

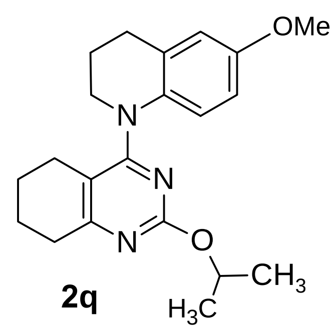

2q

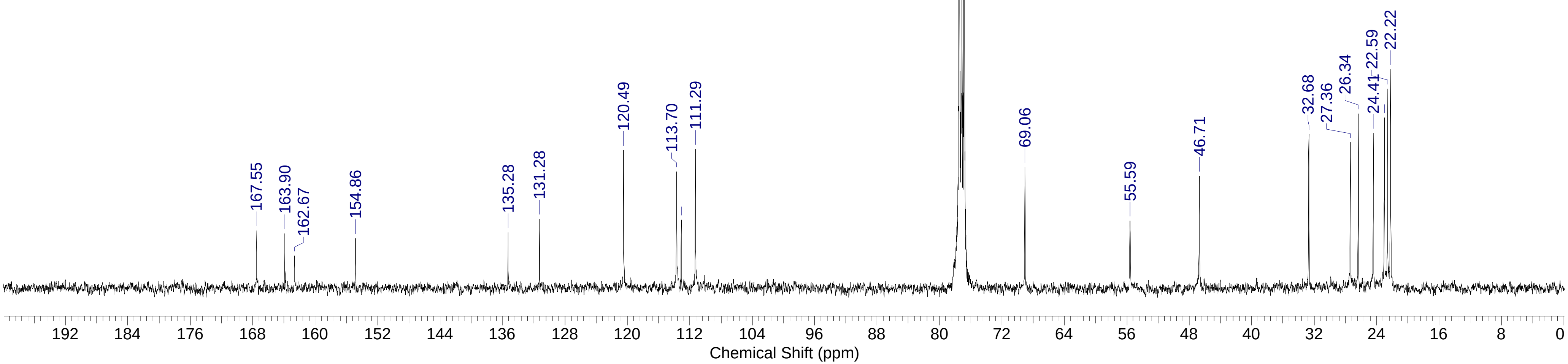

^{1}H NMR ($CDCl_3$) spectrum of compound **2r**

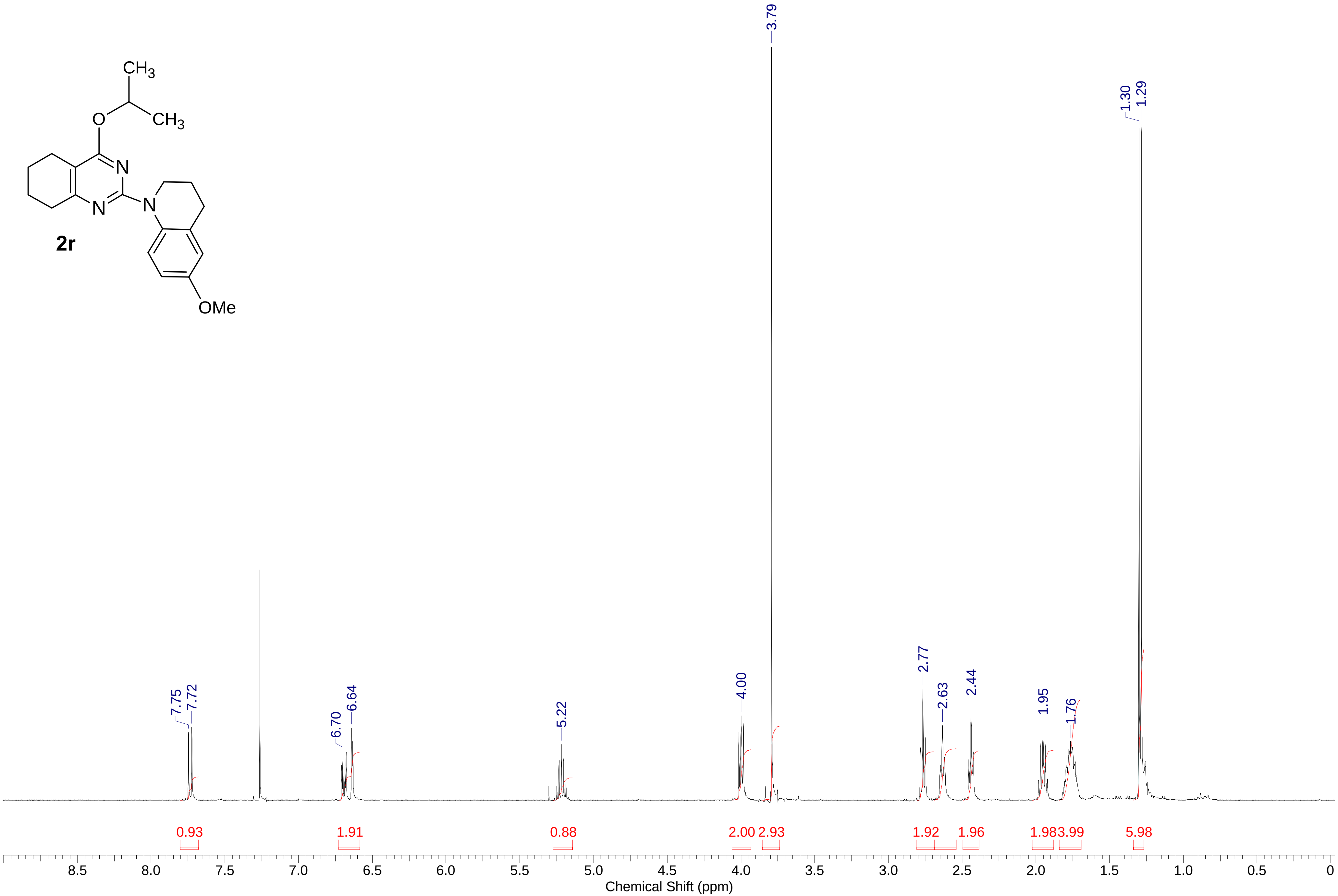

^{13}C NMR ($CDCl_3$) spectrum of compound **2r**

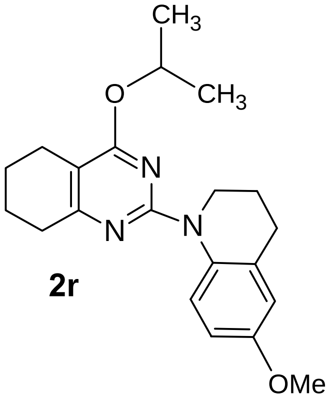

2r

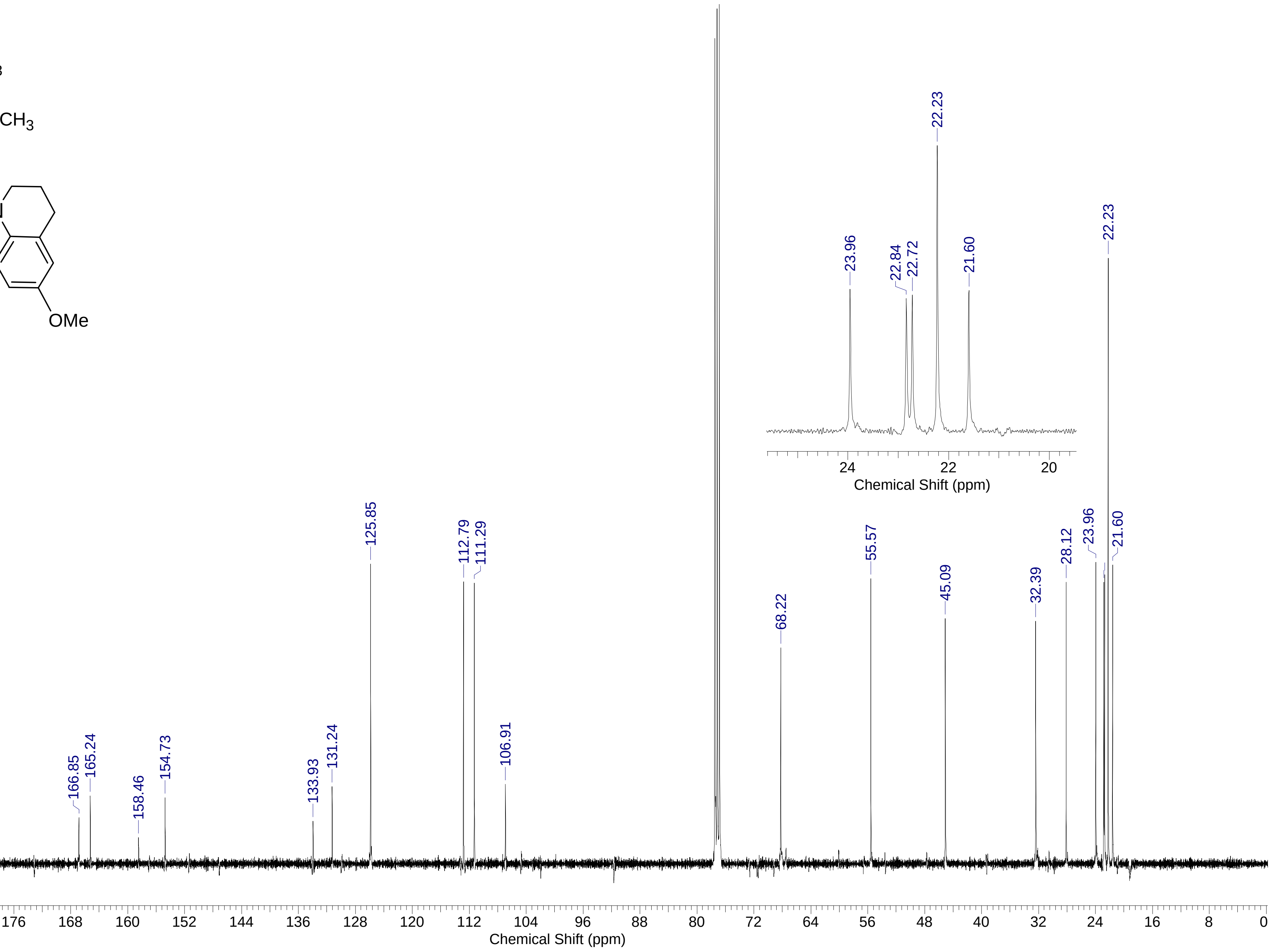

HSQC NMR ($CDCl_3$) spectrum of compound **2r**

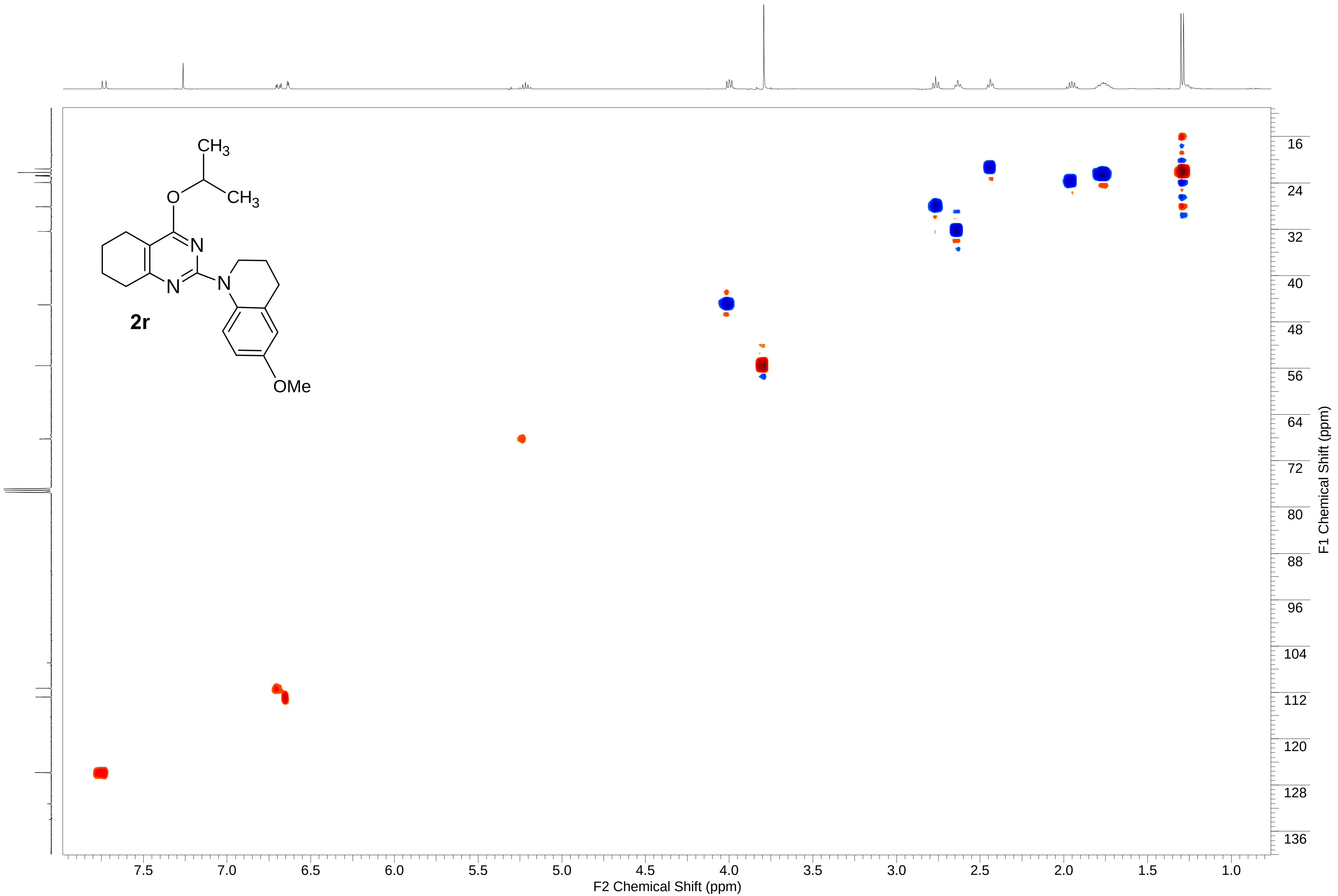

HMBC NMR ($CDCl_3$) spectrum (low field) of compound **2r**

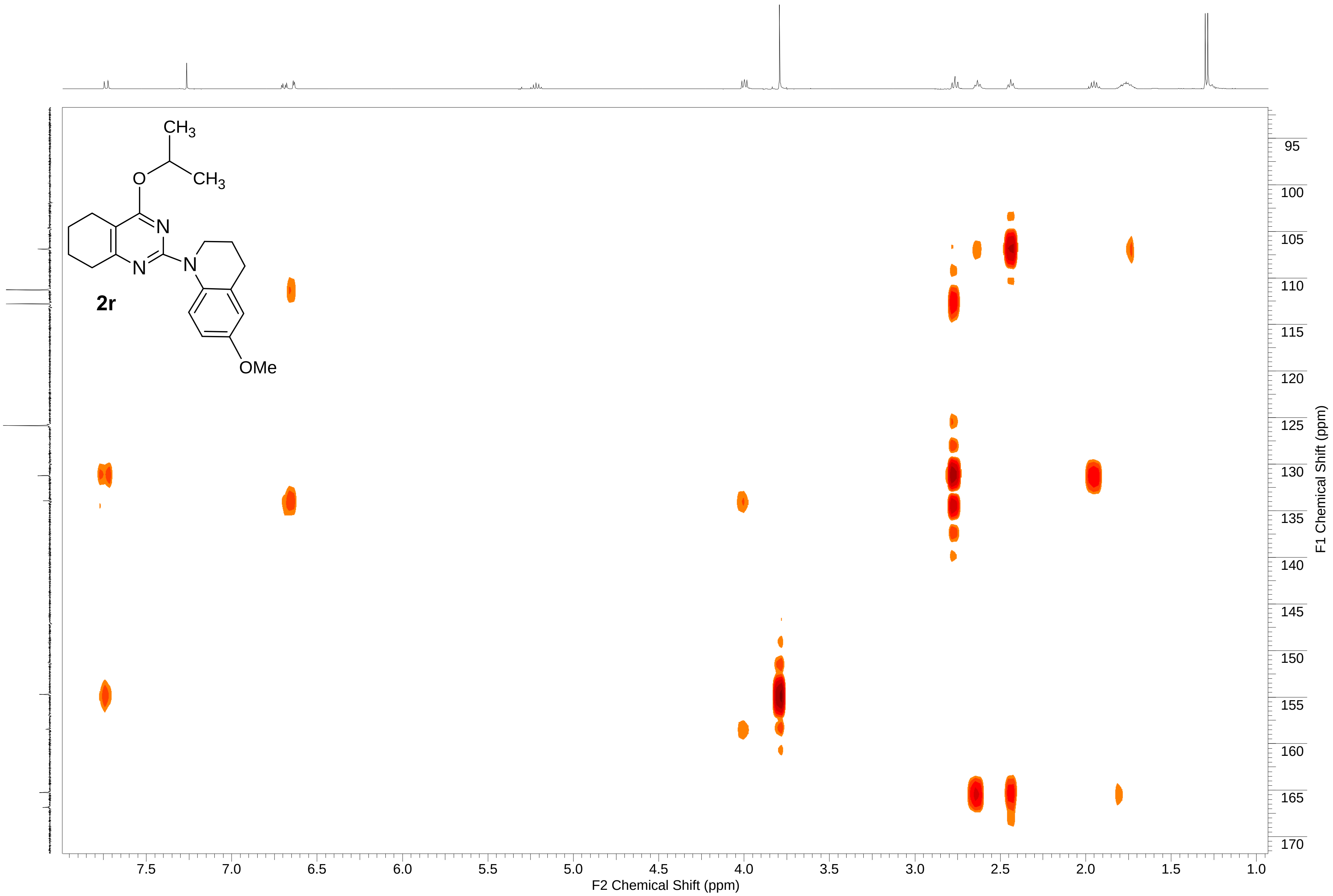

Supplement: Supplementary file 1 [file pharmaceuticals-16-01499-s001.zip › pharmaceuticals-2638694-supplementary.pdf]
